# IRF1-mediated sensing of oxidized mitochondrial DNA drives macrophage PANoptosis in lung ischemia–reperfusion injury

**DOI:** 10.1007/s10495-026-02401-3

**Published:** 2026-07-25

**Authors:** Nan Zhang, Zhiyuan Zhang, Jing Yu, Yu Fu, Jiameng Gao, Xuemei Jiang, Yang Jin, Chang Chen, Zongmei Wen

**Affiliations:** 1 Department of Anesthesiology, Shanghai Pulmonary Hospital, School of Medicine, Tongji University, Shanghai, China; 2 Department of Anesthesiology and Critical Care, Shanghai First Maternity and Infant Hospital, School of Medicine, Tongji University, Shanghai, China; 3 Department of Anesthesiology, Shanxi Bethune Hospital, Shanxi Academy of Medical Sciences, Third Hospital of Shanxi Medical University, Tongji Shanxi Hospital, Taiyuan, China; 4 Department of Thoracic Surgery, Shanghai Pulmonary Hospital, School of Medicine, Tongji University, Shanghai, China

**Keywords:** Lung ischemia–reperfusion injury, Oxidized mitochondrial DNA, IRF1, NLRC5, Alveolar macrophages, PANoptosis

## Abstract

Lung ischemia–reperfusion injury (IRI), a significant factor contributing to early mortality following lung transplantation (LTx), is driven by molecular mechanisms that are not yet fully understood. In this study, we elucidate a crucial signaling pathway initiated by extracellular histones (ex-His), which connects mitochondrial damage to inflammatory cell death. Through the use of a murine model of lung IRI and *in vitro* experiments with alveolar macrophages, we demonstrate that ex-His facilitate Drp1-dependent mitochondrial fission, resulting in the release of oxidized mitochondrial DNA (ox-mtDNA) into the cytosol. Our mechanistic analysis reveals that cytosolic ox-mtDNA is detected by the transcription factor IRF1, which subsequently upregulates NLRC5 at the transcriptional level. Additionally, we identify NLRC5 as a non-canonical scaffold that is essential for the assembly of the PANoptosome, a multi-protein complex that mediates PANoptosis, an inflammatory cell death pathway that aggravates tissue damage. The clinical relevance of this pathway was then examined in lung transplant recipients. An analysis of their peripheral blood mononuclear cells (PBMCs) revealed that patients who developed primary graft dysfunction (PGD) exhibited markedly elevated levels of circulating histones and cytosolic ox-mtDNA. This was associated with hyperactivation of the core signaling axis, characterized by increased Drp1 phosphorylation and elevated expression of IRF1 and NLRC5. Collectively, our study elucidates a comprehensive pathogenic cascade from an extracellular danger signal to a specific cell death program, identifying the ex-His-mitochondria-IRF1-NLRC5 axis as a critical driver of macrophage PANoptosis and a promising therapeutic target for alleviating lung IRI.

**Supplementary Information:**

The online version contains supplementary material available at 10.1007/s10495-026-02401-3.

## Introduction

Lung ischemia–reperfusion injury (IRI) is a significant clinical challenge that extends beyond solid organ transplantation to various pathological conditions, including pulmonary embolism, cardiopulmonary bypass surgery, and cardiac arrest [1]. Within the realm of lung transplantation (LTx), IRI is the principal driver of primary graft dysfunction (PGD), a severe form of acute lung injury that affects up to 30% of recipients and remains a predominant factor contributing to early postoperative morbidity and mortality [2, 3]. The pathophysiological mechanisms underlying lung IRI are delineated by a biphasic process, commencing with oxidative stress and cellular alterations during the ischemic phase, followed by a pronounced inflammatory response upon reperfusion. This sterile inflammation is precipitated by the release of damage-associated molecular patterns (DAMPs), which instigate the activation and recruitment of innate immune cells, complement activation, and the secretion of inflammatory mediators [4]. The ensuing dysregulated inflammatory cascade exacerbates cellular injury and death, perpetuating a deleterious cycle that culminates in substantial tissue damage. A deeper understanding of how these initial sterile insults are translated into specific, programmed cell death (PCD) pathways in resident immune cells is essential for developing effective therapies for PGD.

Extracellular histones (ex-His), key DAMPs, are implicated in acute sterile inflammation and are released from dying or activated cells during conditions such as sepsis, trauma, and acute organ injury [5]. Emerging evidence underscores the role of ex-His in mediating IRI and contributing to acute organ damage [6–8]. In the context of LTx, preliminary clinical studies have demonstrated that plasma levels of ex-His are elevated in patients with PGD compared to those without, with this elevation correlating with the severity of the inflammatory response [9]. However, the specific intracellular mechanisms by which they program cell death in resident immune cells remain poorly defined.

Mitochondrial dysfunction represents a central event in the progression of ischemic and hypoxic injury [10]. Under physiological conditions, the equilibrium between mitochondrial fission and fusion is crucial for the maintenance of a healthy mitochondrial network. In response to cellular stress, this equilibrium is disrupted, resulting in excessive mitochondrial fragmentation, which subsequently impairs energy production and compromises the stability of mitochondrial DNA (mtDNA). This process, often mediated by the fission protein Drp1, is also associated with the generation of reactive oxygen species (ROS) and a failure in mitophagy, the process for the clearance of damaged mitochondria [11]. Such deficiencies in mitochondrial quality control can lead to the release of DAMPs, including oxidized mtDNA (ox-mtDNA), into the cytoplasm, where they can activate the innate immune system.

Interferon Regulatory Factor 1 (IRF1) is a transcription factor that plays a central role in innate immune responses by regulating the expression of genes involved in inflammation and antiviral immunity. Its importance is well-established in organ transplantation and IRI [12]. It can be activated by various pathogen- and damage-associated molecular patterns (PAMPs and DAMPs), particularly cytosolic nucleic acids [13]. Recent studies underscore the pivotal role of IRF1 as a downstream effector in mtDNA sensing pathways. Activation of IRF1 by cytosolic mtDNA initiates the transcription of pro-inflammatory genes, thereby enhancing the immune response [14, 15]. Notably, IRF1 has been identified as a direct upstream regulator that transcriptionally upregulates NOD-like receptor family, CARD domain containing 5 (NLRC5) [16, 17]. While the individual roles of IRF1 in cell death and mitochondrial stress in organ injury are well-documented, a cohesive signaling pathway that connects these events to program a specific cell death modality like PANoptosis in lung IRI has not been established.

The NOD-like receptor (NLR) family is instrumental in detecting DAMPs and modulating innate immunity. Within this family, NLRC5 has emerged as a regulator of inflammation, cell death, and fibrosis [18–20]. Although its role has been documented in various organ IRI models [21–23], the specific involvement of NLRC5 in lung IRI and the upstream signals regulating its activity are largely unexplored. Simultaneously, emerging paradigms in cell death have introduced the concept of PANoptosis, a form of programmed inflammatory cell death that integrates key characteristics of pyroptosis, apoptosis, and necroptosis [24, 25]. PANoptosis, first proposed in 2016, represents an inflammatory form of PCD that is activated by pathogens, PAMPs, DAMPs, and various other stimuli. This process is orchestrated by the PANoptosome, a multiprotein complex that assembles intracellularly and functions as a molecular scaffold. Assembly of this platform is known to involve the sensor NLRC5, the adaptor ASC (Apoptosis-associated speck-like protein containing a CARD), and key effector molecules such as RIPK3 (Receptor-interacting serine/threonine-protein kinase 3) and CASP8 (Caspase-8). PANoptosis is distinguished by the hallmark features of pyroptosis, apoptosis, and/or necroptosis, yet it cannot be fully accounted for by any single one of these three PCD pathways [26]. Although PANoptosis has been associated with the progression of organ IRI and subsequent failure [27–29], its specific role and regulatory mechanisms in the pathogenesis of lung IRI remain to be clarified. Therefore, it remains unclear how danger signals originating from damaged mitochondria are specifically interpreted by the IRF1-NLRC5 axis to orchestrate this complex cell death in alveolar macrophages (AMs).

This study investigates the hypothesis that IRF1 acts as a crucial sensor of mitochondrial stress, converting the danger signal from extracellular histones into a programmed inflammatory cell death response that drives lung injury. Our work delineates an intricate signaling cascade initiated by ex-His, which induce Drp1-mediated mitochondrial damage and the subsequent release of ox-mtDNA. We demonstrate that this cytosolic ox-mtDNA is sensed by IRF1, which in turn drives NLRC5-dependent PANoptosome assembly and executes PANoptosis. By connecting an extracellular DAMP to a specific intracellular cell death program via a mitochondria-to-nucleus signaling axis, our work defines a key pathogenic mechanism in lung injury and identifies this pathway as a promising therapeutic target.

## Materials and methods

### Study participants and sample collection

This investigation was conducted in accordance with the Declaration of Helsinki and received approval from the Institutional Ethics Committee of Shanghai Pulmonary Hospital (Ethical Approval No. K22-267Z). Prior to enrollment, written informed consent was obtained from all participants or their legal guardians. In accordance with national regulations, all donor lungs were procured by certified Organ Procurement Organizations (OPOs) and allocated through the mandatory China Organ Transplant Response System (COTRS). Standardized procurement protocols were followed to minimize donor “down time” and ensure organ viability. All organs were from donors after brain death (DBD) or donation after circulatory death (DCD). We explicitly declare that no organs were obtained from prisoners. All organ donations were voluntary and conducted in strict accordance with the ethical guidelines of the Declaration of Istanbul. A cohort of 30 patients scheduled for LTx at our institution between June 2023 and June 2024 was prospectively recruited for this study. Their baseline characteristics, including detailed donor and perioperative data, are presented in Supplementary Table S1. To ensure data security and protect patient privacy, all clinical information was de-identified and assigned unique alphanumeric codes. These data were managed in a secure, restricted-access database in compliance with institutional data protection policies. Primary graft dysfunction (PGD) was graded within the first 72 h post-transplantation according to the International Society for Heart and Lung Transplantation (ISHLT) consensus criteria, which are based on the PaO₂/FiO₂ ratio and the presence of lung infiltrates on chest radiographs. Notably, all LTx recipients received a standardized perioperative management protocol, including induction therapy with basiliximab and a triple-drug maintenance immunosuppressive regimen (tacrolimus, mycophenolate mofetil, and glucocorticoids), supplemented by standard prophylactic anti-infectives. Eligibility criteria included adult patients aged 18 to 80 years with a comprehensive clinical history. A control group comprising 20 healthy volunteers, matched for age and sex, was also enrolled. In the patient cohort, serial venous blood samples (10 mL) were collected via a central venous catheter at four time points corresponding to key phases of IRI and PGD development: immediately prior to surgery (preoperative baseline), and at 24, 48, and 72 h postoperatively. For the healthy control group, a single 10 mL sample of peripheral venous blood was obtained via venipuncture. We processed all blood samples within two hours of collection for subsequent analysis. Patients from whom a complete set of postoperative samples could not be obtained were excluded from the final data analysis.

### Animal model and experimental procedures

Male C57BL/6J mice, aged 6–7 weeks and weighing 18–22 g, were procured from Shanghai SLAC Laboratory Animal Co., Ltd. Young male mice were exclusively utilized in this study to eliminate the confounding immunomodulatory and protective effects of the female estrous cycle and to avoid baseline mitochondrial alterations frequently associated with aging. Animals were maintained in a specific-pathogen-free facility under a 12-h light/dark cycle with ad libitum access to standard chow and water. All animal experiments received approval from the Ethics Committee of Shanghai Pulmonary Hospital (Approval No. K23-199) and were conducted in accordance with institutional guidelines. A left lung IRI model was established as previously described [30]. In brief, following thoracotomy, the left pulmonary hilum was occluded using an atraumatic microvascular clamp (sufficient to halt blood flow without inducing mechanical crush injury) for 2 h, followed by 4 h of reperfusion. The completeness of ischemia was consistently confirmed by immediate visual blanching of the lung tissue. This 2 h/4 h time course is a well-established model that robustly induces acute inflammatory injury. For the control group (referred to as “Control” throughout), mice underwent the same surgical procedure but without occlusion of the pulmonary hilum. Sample sizes were statistically determined prior to the experiments based on a power analysis using G*Power software (version 3.1.9.7). Assuming an anticipated large effect size (Cohen’s d = 1.8) for critical primary outcomes (e.g., PaO₂/FiO₂ ratio changes), an alpha (α) of 0.05, and a statistical power (1 − β) of 0.80, a minimum cohort size of 6 mice per group (n = 6) was calculated and applied to ensure robust statistical significance while adhering to ethical rules of animal reduction.

Mice were randomly assigned to five groups: (1) Control, (2) IRI with Vehicle, (3) IRI with N-acetylheparin (Heparin), (4) IRI with Negative Control siRNA (si-NC), and (5) IRI with si-IRF1. Following randomization, a multi-group co-housing strategy was employed to mitigate potential cage effects and environmental confounders. Specifically, mice from different experimental groups were co-housed within the same cages (strictly limited to a maximum of 5 mice per cage in compliance with institutional ethical guidelines). This design ensured that any baseline environmental variances were equally distributed across all treatment groups. For *in vivo* gene silencing, small interfering RNA (siRNA) complexes were administered 48 h prior to IRI surgery to ensure maximal target gene knockdown at the time of injury. A pre-validated set of three Stealth™ RNAi siRNAs targeting mouse Irf1 (Cat. MSS275123, MSS275124, and MSS275125) and a Stealth™ RNAi siRNA Negative Control (Cat. 12,935,300) were purchased from Thermo Fisher Scientific (USA). The siRNA delivery complexes were prepared by mixing siRNA (0.5 mg/kg body weight) with in vivo-jetPEI® (Polyplus-transfection, Illkirch, France) in a 10% glucose solution, adhering to the manufacturer’s protocol. Following a 15-min incubation at room temperature to allow for complex formation, the mixture was administered. For local lung delivery, mice were anesthetized, and a 50 μL volume of the siRNA complex was instilled via a non-surgical endotracheal catheter, followed immediately by a 150 μL gentle air-puff to facilitate distribution to the lower airways. For pharmacological treatment, N-acetylheparin (250 U/kg; Sigma-Aldrich), a chemically modified non-anticoagulant heparin derivative used for histone neutralization, was administered via intravenous injection 30 min prior to the induction of ischemia. At the conclusion of the reperfusion period, mice were euthanized, and samples of the left lung tissue, whole blood, and bronchoalveolar lavage fluid (BALF) were collected for subsequent analysis.

### Cell culture and treatments

The murine alveolar macrophage cell line, MH-S (ATCC, USA), was cultured in RPMI 1640 medium (HyClone, USA) supplemented with 10% fetal bovine serum (FBS; Gibco, USA) and 1% penicillin–streptomycin (Gibco, USA). To maintain consistency, all experiments were conducted with cells between passages 5 and 15, and a single lot of FBS was used throughout the study. The cells were maintained in a humidified incubator at 37 °C with 5% CO₂. For experimental procedures, cells were seeded into 6-, 24-, or 96-well plates, and cultured until they reached approximately 80% confluence.

Histone Stimulation and Inhibition. For histone stimulation assays, cells were treated with 50 μg/mL of calf thymus histone (Sigma-Aldrich, USA) for a duration of 3 h, a concentration and duration determined by preliminary dose–response experiments and consistent with previous studies. In inhibitor studies, cells underwent a 2-h pretreatment with either the mitochondrial ROS scavenger Mito-TEMPO (100 μM; Sigma-Aldrich, USA) or the mitochondrial fission inhibitor Mdivi-1 (10 μM; Selleck, USA) [31]. Both inhibitors were dissolved in dimethyl sulfoxide (DMSO). Following pretreatment, cells were co-stimulated with histones (50 μg/mL) for an additional 3 h. The vehicle control group was administered equivalent volumes of DMSO in PBS.

Gene Knockdown by siRNA. For gene knockdown experiments, MH-S cells were transfected with siRNAs (GenePharma, Shanghai, China) targeting Irf1 or Nlrc5, or with a non-targeting negative control siRNA (si-NC), using Lipofectamine 2000 (Invitrogen, USA) in accordance with the manufacturer's protocol. All subsequent experiments were conducted 48 h post-transfection to allow for effective target protein depletion. The siRNA sequences employed were as follows: si-Irf1 (Sense, 5’-CCAGCUCUCUUCUGUCUAUTT-3’; Antisense, 5’-AUAGACAGAAGAGAGCUGGTT-3’) and si-Nlrc5 (Sense, 5’-UCAAGUUAAAUUUCCUGAGUUCUGGTT-3’; Antisense, 5’-CCAGAACUCAGGAAAUUUAACUUGATT-3’).

### Cell viability assay

Macrophage viability was evaluated using the Cell Counting Kit-8 (CCK-8) Plus (Cat. No. K2268, APExBIO, USA) according to the manufacturer’s instructions. Briefly, MH-S cells were seeded into 96-well culture plates at a volume of 100 μL per well and allowed to adhere. To assess dose- and time-dependent cytotoxicity, the cells were treated with calf thymus histone at indicated concentrations (0, 25, 50, and 100 μg/mL) for various durations (0, 3, 6, 12, and 24 h). Following the respective treatments, 10 μL of CCK-8 Plus reagent was added to each well, carefully avoiding the introduction of bubbles. The plates were then incubated for 1 h at 37 °C in a humidified atmosphere containing 5% CO₂. The optical density (OD) was measured at a wavelength of 450 nm using a multimode microplate reader (Thermo Fisher Scientific, Waltham, MA, USA). Five replicate wells (n = 5) were established for each experimental group. Relative cell viability was calculated as a percentage of the absorbance of the treated cells compared to that of the untreated control cells.

### Live/dead cell staining

Morphological visualization of macrophage viability was performed utilizing a Calcein-AM/PI Double Stain Kit (Cat. No. 40747ES76, Yeasen Biotechnology, Shanghai, China). Following exposure to calf thymus histone (50 μg/mL) for 3 h, MH-S cells were thoroughly rinsed with PBS to eliminate extracellular esterases prior to labeling. The cells were then co-incubated with the Calcein-AM/PI dual-dye mixture for 15 min at 37 °C, protected from light. Fluorescent microphotographs were promptly acquired using an inverted fluorescence microscope (Olympus, Tokyo, Japan). Through this dual-labeling strategy, enzymatically active living cells are characterized by intense green fluorescence resulting from Calcein-AM cleavage, whereas non-viable cells with compromised membrane integrity exhibit red fluorescence due to PI intercalation with nuclear DNA.

### Isolation of peripheral blood mononuclear cells (PBMCs)

Peripheral blood samples were collected from human subjects into EDTA-containing tubes. Plasma was first separated from whole blood by centrifugation (1500 × g, 10 min, room temperature). After collecting and storing the plasma supernatant at −80 °C, the remaining cellular fraction was diluted 1:1 with sterile PBS for isolation of PBMCs via density gradient centrifugation. This diluted blood was carefully layered over an equal volume of Ficoll-Paque™ PLUS (1.077 g/mL; Cytiva). Centrifugation at 800 × g for 20 min, with the brake disengaged to preserve the interface, effectively separated the mononuclear cells. The distinct mononuclear cell layer, visible as a buffy coat, was then carefully aspirated, transferred to a new tube, and washed with PBS (300 × g, 10 min). To remove any contaminating erythrocytes, the cell pellet was briefly incubated (2–3 min) in 1 mL of 1 × RBC Lysis Buffer (BD Biosciences, USA). The lysis was quenched by the addition of excess PBS, after which the cells were pelleted one final time (300 × g, 10 min). The final, clean PBMC pellet was then ready for subsequent analyses.

### Blood gas analysis and lung wet-to-dry (W/D) ratio

Pulmonary gas exchange and edema, two key metrics of lung injury, were assessed immediately post-euthanasia. The procedure began with the time-sensitive collection of arterial blood (~0.5 mL) via cardiac puncture into a pre-heparinized syringe. This sample was instantly analyzed for its partial pressure of oxygen (PaO₂) with a portable blood gas analyzer (Shengchang Medical Technology Co., Ltd., Shanghai, China). Immediately following blood collection, the entire left lung was meticulously excised, briefly rinsed in cold PBS, and blotted to remove excess surface moisture before its initial mass was recorded as the wet weight (W). The tissue was then desiccated in an 80 °C oven for 48 h to achieve a constant mass, which provided the dry weight (D). The final lung W/D ratio, a direct index of pulmonary edema, was calculated from these two weights.

### Histological analysis

The structural integrity of the lung tissue was examined through histological staining. Immediately upon excision, left lung lobes were submerged in 4% paraformaldehyde for at least 24 h, a process that preserved their architecture for downstream analysis. These fixed tissues were then prepared for sectioning through a sequential process of dehydration in graded ethanol, clearing in xylene, and infiltration with molten paraffin before being embedded. From the resulting paraffin blocks, thin 5 μm sections were cut and carefully mounted onto glass slides. To visualize cellular detail, the sections first underwent deparaffinization and rehydration, followed by standard hematoxylin and eosin (H&E) staining. This classic staining combination revealed the morphological landscape of the lung, which was then systematically examined under a light microscope (Olympus, Tokyo, Japan) to document pathological changes, with representative fields being captured for analysis.

### Immunofluorescence (IF) staining

The spatial localization of key proteins was visualized by immunofluorescence in both cultured cells and lung tissue sections. Sample preparation, however, differed by type. For MH-S cells and PBMCs cultured on coverslips, the process began with a 15-min fixation in 4% paraformaldehyde (PFA), followed by permeabilization with 0.1% Triton X-100 in PBS to allow antibody access to intracellular targets. Paraffin-embedded lung tissue sections, in contrast, first required deparaffinization and rehydration. Critically, these sections then underwent heat-induced antigen retrieval in a citrate buffer (10 mM, pH 6.0) to unmask epitopes, followed by a permeabilization step using a buffer containing 0.3% Triton X-100.

Once prepared, both sample types were incubated in a blocking solution of 5% bovine serum albumin for one hour to minimize nonspecific antibody binding. They were then incubated overnight at 4 °C with primary antibodies targeting key components of the PANoptosome, mitochondrial markers, and indicators of cellular stress (a detailed list of primary antibodies and their working dilutions is provided in Supplementary Table S2). After thorough washing, the bound primary antibodies were detected using corresponding Alexa Fluor-conjugated secondary antibodies during a 1-h incubation in the dark. Finally, nuclei were counterstained with DAPI (C1002, Beyotime).

Imaging was performed using two complementary approaches. High-resolution confocal microscopy (Nikon, Japan) was used to capture detailed images of subcellular structures like PANoptosomes and mitochondria from at least five random fields per condition. For large-scale, comprehensive views, whole slides were digitized with a Pannoramic 250 scanner (3D Histech, Hungary). To move beyond qualitative observation, mitochondrial network morphology from Tom20-stained cell images was quantified using the MiNA plugin in ImageJ, where parameters such as mean branch length and network branches served as metrics for mitochondrial fission/fusion status.

### Western blot analysis

Western blot analysis was employed to quantify changes in protein abundance and activation state. Total protein was extracted from cultured cells and lung tissue homogenates using a RIPA lysis buffer supplemented with protease inhibitors. After clarifying the lysates by centrifugation (12,000 × g, 15 min, 4 °C), the protein concentration of the resulting supernatant was determined with a BCA assay. For each sample, equal amounts of protein were denatured, resolved by SDS-PAGE, and subsequently transferred onto PVDF membranes.

Following a 1-h blocking step in 5% non-fat milk, the membranes were incubated overnight at 4 °C with a suite of primary antibodies selected to probe the core machinery of PANoptosis, inflammation, and mitochondrial dynamics (a detailed list of primary antibodies and their dilutions is provided in Supplementary Table S3). After thorough washing, membranes were treated with the appropriate HRP-conjugated secondary antibodies (#AS014, #AS003; ABclonal, USA) for one hour. Protein bands were then visualized using an ECL substrate (Biosharp, China), and chemiluminescent signals were captured with a ChemiScope 6000 imaging system (CLiNX, China).

The captured band intensities were quantified using ImageJ software. To ensure accurate comparison, the expression level of each target protein was normalized to its corresponding β-actin signal, which served as the internal loading control.

### Measurement of ex-His and inflammatory cytokines

The concentrations of ex-His and a panel of inflammatory cytokines—namely interleukin-6 (IL-6), interleukin-1β (IL-1β), and tumor necrosis factor-alpha (TNF-α)—were determined in mouse bronchoalveolar lavage fluid (BALF) and plasma. All analytes were quantified using commercial enzyme-linked immunosorbent assay (ELISA) kits, strictly following the manufacturers’ protocols. Specifically, ex-His levels were measured using the Cell Death Detection ELISA PLUS Kit (Roche Diagnostics GmbH, Germany), whereas the concentrations of IL-6, IL-1β, and TNF-α were determined using their respective QuickCyto® ELISA kits (NeoBioscience, China).

### Measurement of mitochondrial reactive oxygen species (mtROS) and membrane potential (ΔΨm)

Mitochondrial function was evaluated by assessing mitochondrial ROS and ΔΨm through fluorescence microscopy. Mitochondrial ROS levels were determined by staining cells with 2 µM of the mtROS-specific probe MitoSOX™ Red (S0061S, Beyotime, China) for 15 min at 37 °C in the dark. Following incubation, cells were washed twice with warm PBS to remove excess probe prior to imaging. Changes in ΔΨm were assessed using a JC-1 Assay Kit (C2006, Beyotime, China). Following the manufacturer's protocol, cells were incubated with the JC-1 staining working solution for 20 min at 37 °C. Subsequently, the cells underwent two washes with the provided JC-1 buffer. A reduction in mitochondrial ΔΨm is evidenced by a fluorescence shift from red, indicating J-aggregates in healthy mitochondria, to green, representing J-monomers in depolarized mitochondria. Fluorescence images were captured using a Zeiss LSM 880 confocal laser scanning microscope (Zeiss, Germany).

### Ultrastructural analysis by transmission electron microscopy (TEM)

For ultrastructural analysis, MH-S cells were subjected to a multi-step preparation protocol for transmission electron microscopy. The process began with chemical fixation, where specimens were initially preserved for two hours at 4 °C in 2.5% glutaraldehyde (0.1 M phosphate buffer, pH 7.4), and subsequently post-fixed for one hour in 1% osmium tetroxide to ensure lipid stabilization. Following fixation, the samples underwent systematic dehydration in a graded ethanol series before being infiltrated with and embedded in Spurr’s resin. The resulting resin blocks were sectioned into 60–80 nm ultrathin slices using an ultramicrotome. These sections, carefully mounted on copper grids, were then double-stained with uranyl acetate and lead citrate to generate sufficient electron contrast. The prepared grids were examined on a Hitachi HT7700 transmission electron microscope (Japan) operating at 80 kV, and high-resolution digital images of the cellular and mitochondrial architecture were acquired with an integrated CCD camera.

### Isolation of cytosolic mtDNA and qPCR analysis

The isolation of cytosolic mtDNA was achieved using a digitonin-based selective permeabilization protocol. Following two washes in ice-cold PBS, harvested PBMCs or MH-S cells were resuspended in a hypotonic buffer containing a low concentration of digitonin (0.025%; 150 mM NaCl, 50 mM HEPES, with protease inhibitors) and incubated for 10 min on ice. This treatment selectively lyses the plasma membrane while preserving mitochondrial integrity. A subsequent high-speed centrifugation (10,000 × g, 10 min, 4 °C) pelleted these intact organelles and nuclei, thereby isolating the cytosolic fraction in the supernatant. DNA was then extracted from this purified supernatant with the QIAamp DNA Micro Kit (Qiagen, Germany), a process that included an RNase A treatment to eliminate RNA contamination. The final DNA concentration was determined via NanoDrop spectrophotometry.

The extracted DNA served as the template for quantifying cytosolic mtDNA levels by quantitative real-time PCR (qPCR). This analysis targeted three distinct mitochondrial genes (D-loop, Cytb, and mt-ND1), using the nuclear Tert gene as a negative control to rule out genomic DNA contamination. All primer sequences are detailed in Supplementary Table S4. Reactions were prepared using SYBR® Premix Ex Taq™ II (Takara, Japan) and performed on a QuantStudio 5 Real-Time PCR System (Applied Biosystems, USA). The thermal profile consisted of an initial 30-s denaturation at 95 °C, followed by 40 cycles of 95 °C for 5 s and 60 °C for 30 s. The relative abundance of cytosolic mtDNA was calculated via the 2^^−ΔCt^ method, normalized to the total DNA input for each sample. Each sample was analyzed in triplicate, and all experiments were independently performed at least three times.

### Molecular docking and interaction analysis

The structural basis for the interactions among ASC, CASP8, NLRC5, and RIPK3 was investigated through *in silico* protein–protein molecular docking. This analysis utilized atomic coordinates for the mouse proteins sourced from the UniProt database (IDs: Q9EPB4 for ASC, O89110 for CASP8, C3VPR64 for NLRC5, and Q9QZL0 for RIPK3). All docking simulations were performed on the HDOCK server, which employs a hybrid algorithm of template-based modeling and *ab initio* free docking. A sequential, hierarchical strategy was adopted to assemble the final quaternary complex. The process commenced by docking RIPK3 (as the ligand) onto NLRC5 (as the receptor) to form the initial heterodimer. The resulting top-scoring NLRC5-RIPK3 model then served as a composite receptor for the subsequent docking of CASP8. In the final step, this newly formed trimeric complex (NLRC5-RIPK3-CASP8) acted as the receptor for the docking of ASC.

### Bulk RNA-seq analysis of lung IRI

Transcriptomic alterations in a murine model of lung IRI (60 min of ischemia, 120 min of reperfusion) were investigated through re-analysis of the publicly accessible dataset GSE203238 from the Gene Expression Omnibus (GEO). Following processing of the raw count matrix with the DESeq2 package in R (v4.4.3), differentially expressed genes (DEGs) between the lung IRI and control groups were identified based on thresholds of an adjusted *p*-value < 0.05 and a |log₂(Fold Change)| > 0.5. A multi-faceted analytical strategy was subsequently employed to interpret the biological significance of these transcriptomic changes. Functional enrichment of the DEG list against Gene Ontology (GO) and Kyoto Encyclopedia of Genes and Genomes (KEGG) databases was conducted using the clusterProfiler package, with significance determined at an adjusted *p*-value < 0.05. Complementing this approach, Gene Set Enrichment Analysis (GSEA) was performed on a gene list pre-ranked by log₂(Fold Change) with the fgsea package, interrogating the Hallmark and Reactome gene sets (MSigDB, v2025.1). Finally, Gene Set Variation Analysis (GSVA) was employed to assess pathway-level alterations on a per-sample basis, with a particular focus on those associated with mitochondrial function.

### Single-cell RNA-seq analysis of human lung transplant tissues

Cellular heterogeneity and transcriptomic responses within the human lung transplant microenvironment were interrogated using the publicly accessible single-cell RNA-sequencing dataset GSE220797. This dataset comprises lung tissue samples from six recipients, captured at the critical time points of cold ischemia and subsequent reperfusion. The detailed clinical and demographic metadata for these six cases are summarized in Supplementary Table S5. The raw sequencing data were processed through a pipeline in R (v4.4.3) utilizing the Seurat package (v5). A key feature of this workflow was a customized quality control protocol, intentionally designed to accommodate the anticipated cellular stress of IRI; cells were retained if they expressed over 200 unique genes and possessed a mitochondrial gene fraction below a lenient threshold of 35%, a strategy chosen to preserve cells undergoing metabolic stress or early-stage cell death. Following log-normalization and dimensionality reduction via PCA and UMAP, cell clusters were identified using unsupervised graph-based clustering (Louvain algorithm). Specifically, the broad lung macrophage population was annotated and confirmed based on the robust expression of canonical pan-macrophage markers (e.g., CD68, MRC1, and AIF1). This macroscopic macrophage cluster was evaluated as a whole to capture the dynamic transcriptomic landscape of the heterogeneous in vivo immune environment during IRI. To delineate the specific transcriptional shift induced by the injury, DEGs within this macrophage cluster between the reperfusion and cold ischemia (CIT) groups were identified using a threshold of an adjusted *p*-value < 0.05 and |log₂(Fold Change)| > 0.25.

Subsequent, more granular analysis converged on the macrophage population within the reperfusion samples to dissect its functional heterogeneity. A dual-metric stratification strategy was devised, first categorizing macrophages by their normalized IRF1 expression levels (using 30th and 70th percentiles as low/high thresholds). Concurrently, an “immune activity score” was computed for each cell by averaging the expression of a curated panel of immune genes (e.g., CD3D, TNF, IFNG, CASP3, NLRP3, IL1B). The integration of these two metrics delineated four distinct functional subsets: Synergy_High (high IRF1, high immune score), IRF1_SoloHigh (high IRF1, low immune score), Immune_SoloHigh (low IRF1, high immune score), and Synergy_Low (low IRF1, low immune score). To resolve the dynamic activation of PANoptosis within this population, pseudotime trajectory analysis was performed using the Monocle3 package. This approach computationally ordered macrophages along a continuous progression of cellular states, enabling the mapping of expression kinetics for key genes in PANoptosis and mitochondrial function pathways. This mapping thereby elucidated the temporal sequence of gene activation driving the inflammatory response in reperfused lung tissue. The entire bioinformatic workflow and all visualizations were executed in R, leveraging packages such as Seurat, Monocle3, ggplot2, and ComplexHeatmap.

### Statistical analysis

All statistical analyses were executed using GraphPad Prism (v10.1.2) and R software (v4.4.3). Continuous data are presented as mean ± standard deviation (SD) unless otherwise specified. The sample size (n) represents independent biological replicates or individual animals, as detailed in the figure legends. The statistical approach was tailored to the specific characteristics of each dataset. Prior to hypothesis testing, data were evaluated for normality and homogeneity of variances using the Shapiro–Wilk and Levene’s tests, respectively. For comparisons between two independent groups, an unpaired, two-tailed Student’s *t*-test was employed for normally distributed data, whereas the non-parametric Mann–Whitney *U* test was applied otherwise. For comparisons among three or more groups within a single factor, normally distributed data were analyzed using a one-way analysis of variance (ANOVA), followed by either Tukey’s post hoc test for all pairwise comparisons or Dunnett’s post hoc test for comparisons against a control group. Non-parametric datasets were evaluated using the Kruskal–Wallis test with Dunn’s post hoc correction. For experiments evaluating two independent factors simultaneously, a two-way ANOVA was performed, followed by Tukey’s post hoc test for multiple comparisons. Experiments with repeated measurements were analyzed using a repeated-measures ANOVA. The relationships between continuous variables were assessed using either Pearson’s correlation coefficient for linear associations or Spearman’s rank correlation for non-parametric data. Categorical variables, such as clinical characteristics, were evaluated with the chi-square test or Fisher’s exact test, as appropriate for the expected cell counts. Statistical significance was defined as a *P*-value < 0.05, with significance levels in figures denoted as *P < 0.05, **P < 0.01, ***P < 0.001, and ****P < 0.0001.

## Results

### Extracellular histones drive lung injury and PANoptosis during lung IRI

**Fig. 1:**
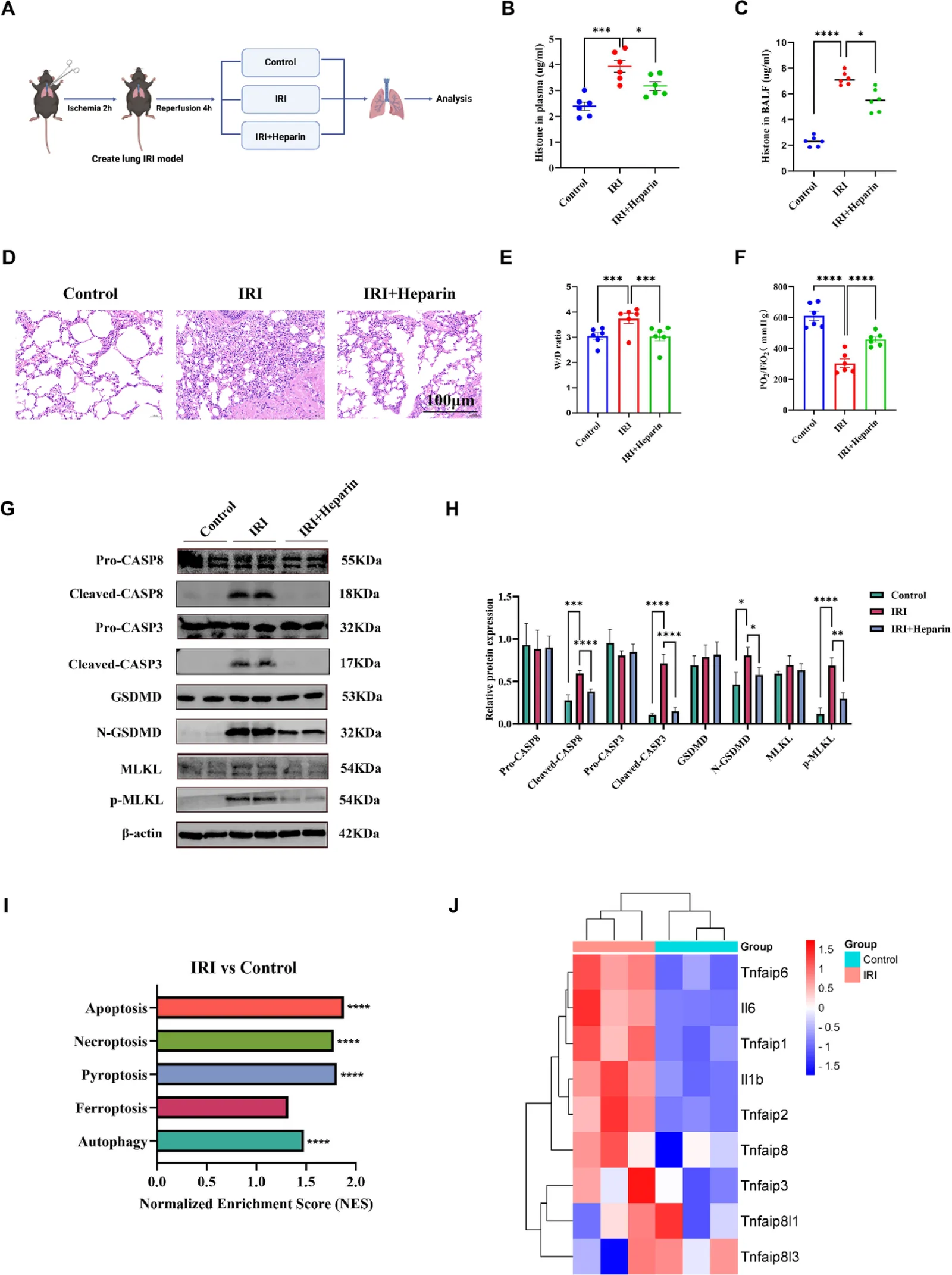
ex-His Drive Pathological Injury and PANoptosis in a Murine Model of Lung IRI. **A** Schematic of the murine lung IRI model induced by left pulmonary hilar clamping. **B**, **C** ELISA quantification of ex-His in plasma (**B**) and BALF (**C**) from mice subjected to IRI. Levels were significantly elevated in the IRI group and reduced by heparin pre-treatment. **D** Representative H&E-stained lung sections showing severe alveolar damage induced by IRI and its amelioration by heparin. Scale bar, 100 μm. **E**, **F** Severe pulmonary edema, determined by the lung wet-to-dry (W/D) ratio (**E**), and impaired gas exchange, assessed by the arterial oxygenation index (PaO₂/FiO₂) (**F**), were evident following lung IRI. Heparin treatment significantly improved both parameters. **G**, **H** Western blot analysis (**G**) and quantification (**H**) of IRI lung lysates showing robust activation of key PANoptosis effectors (Cleaved-CASP8, Cleaved-CASP3, N-GSDMD, and p-MLKL). This activation was markedly suppressed by heparin. **I** GSEA-derived Normalized Enrichment Scores (NES) illustrating the concurrent upregulation of major cell death pathways. **J** Heatmap of bulk RNA-seq data illustrating the upregulation of key proinflammatory cytokine genes (Il1b, Il6, Tnf) in IRI, which was reversed by heparin. Data in (**B**, **C**, **E**, **F**) are presented as mean ± SD (n = 6 per group). Western blots are representative of n = 3 independent experiments. Statistical significance was determined by one-way ANOVA with Tukey’s post-hoc test. *P < 0.05, **P < 0.01, ***P < 0.001, ****P < 0.0001

A murine model of lung IRI, established via left pulmonary hilar occlusion (Fig. 1A), consistently induced a cascade of severe pathologies. The injury profoundly elevated ex-His levels in both plasma and BALF (Fig. 1B, C). This systemic and local release of DAMPs was associated with extensive tissue damage, as demonstrated by histological findings including alveolar septal disruption, inflammatory cell infiltration, and perivascular edema (Fig. 1D). These structural insults corresponded with severe physiological dysfunction in the IRI group, manifested as significant pulmonary edema (increased lung W/D ratio) and impaired gas exchange (reduced PaO₂/FiO₂) (Fig. 1E, F). Notably, pre-treatment with heparin, a known ex-His neutralizing agent, substantially ameliorated this spectrum of pathologies, effectively mitigating the rise in ex-His, preserving lung architecture, and restoring physiological function.

Further investigation into the molecular drivers of this injury revealed the widespread activation of PANoptosis, a coordinated inflammatory cell death program. Transcriptomic re-analysis (GSE203238) from a comparable model showed significant enrichment of apoptosis, necroptosis, and pyroptosis gene sets in IRI lung tissue (Fig. 1I; Supplementary Fig. S1A–C). Protein level corroboration of this transcriptomic signature was obtained through Western blot analysis of lung lysates, confirming robust activation of key PANoptosis effectors, including cleaved CASP8 and CASP3 (apoptosis), the N-terminal fragment of GSDMD (pyroptosis), and phosphorylated MLKL (necroptosis) (Fig. 1G, H). This cell death program unfolded within a hyperinflammatory environment, as evidenced at both the transcriptomic level, indicated by the upregulation of proinflammatory cytokine genes (Fig. 1J) and at the protein level, characterized by elevated concentrations of IL-1β, IL-6, and TNF-α in the BALF (Supplementary Fig. S1D–F). Reinforcing the tight association between these processes, the protein levels of PANoptosis executors strongly and positively correlated with the concentrations of these inflammatory cytokines (Supplementary Fig. S1G–O). Significantly, heparin pre-treatment not only suppressed the activation of PANoptosis effectors but also reversed the inflammatory gene signature, thereby reinforcing the central role of ex-His in driving this inflammatory cell death cascade. Collectively, these data establish that ex-His released during lung IRI are pivotal mediators of pulmonary tissue damage, orchestrating a pathogenic synergy of hyperinflammation and PANoptosis.

### Extracellular histones activate an IRF1–NLRC5 axis to drive macrophage PANoptosis

**Fig. 2:**
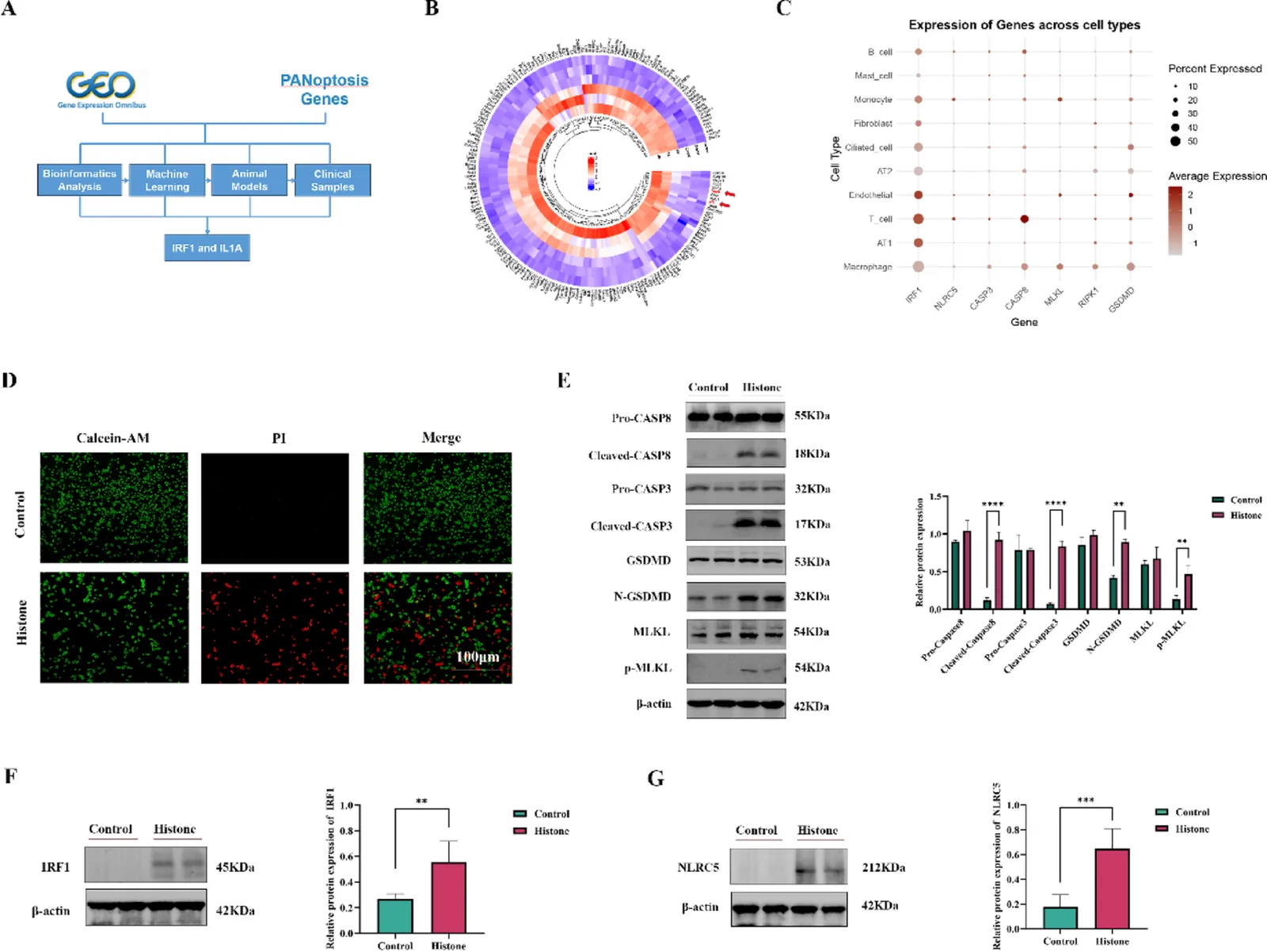
Multi-omics Analysis and In Vitro Validation Pinpoint the IRF1–NLRC5 Axis in Macrophage PANoptosis. **A** Schematic overview of the study design, building upon previously identified candidate genes. **B** Heatmap of PANoptosis-associated differentially expressed genes (DEGs) from bulk RNA-seq data (GSE203238). **C** Dot plot from scRNA-seq analysis (GSE220797) of human lung samples, showing that macrophages are the primary cell type co-expressing IRF1, NLRC5, and key PANoptosis genes. Dot size and color intensity represent the percentage of expressing cells and average expression level, respectively. **D** Ex-His-induced cell death in MH-S macrophages visualized by Calcein-AM (live cells, green) and propidium iodide (PI; dead cells, red) staining. Scale bar, 100 μm. **E** Western blot showing the activation of PANoptosis effectors (Cleaved-CASP8, Cleaved-CASP3, N-GSDMD, p-MLKL) in MH-S cells following ex-His stimulation. **F**, **G** Upregulation of IRF1 (**F**) and NLRC5 (**G**) protein expression in MH-S cells in response to ex-His, shown by Western blot. All in vitro experiments were performed on MH-S cells. Western blots are representative of n = 3 independent experiments. Statistical significance was determined by an unpaired Student’s *t*-test. *P < 0.05, **P < 0.01, ***P < 0.001, ****P < 0.0001

Based on our previously established multi-omics and machine learning workflow [32], IRF1 and IL1A emerged as the candidate genes associated with PANoptosis (Fig. 2A). Given that IRF1 functions as a central transcription factor—making it a more plausible upstream regulator for driving broad cell death and inflammatory networks—we prioritized it to further delineate its specific mechanistic contributions in this study. Analysis of bulk RNA-seq data (GSE203238) from IRI lungs revealed a pronounced PANoptosis-associated gene signature (Fig. 2B), which was significantly enriched in pathways for programmed cell death and innate immunity, such as NOD-like receptor signaling (Supplementary Fig. S2A, B). Pinpointing the cellular origin of this signature required interrogation of single-cell transcriptomic data from human lung transplant recipients (GSE220797). This analysis identified macrophages as a relevant cell type simultaneously co-expressing IRF1, NLRC5, and a suite of key PANoptosis-related genes within individual cells (Fig. 2C), thereby confirming that the observed bulk transcriptomic signature is not an artifact of tissue heterogeneity. The prominence of the IRF1-NLRC5 axis was further supported by *in silico* predictions identifying NLRC5 as a high-confidence downstream target of the transcription factor IRF1 (Supplementary Fig. S2F). We then validated these findings at the protein level *in vivo*. Consistent with our prior immunohistochemical confirmation of elevated IRF1 [32], Western blot analysis showed increased expression of both IRF1 and NLRC5 (Supplementary Fig. S2C, D), and we further confirmed a marked increase in NLRC5 expression via immunofluorescence in IRI lung tissues (Supplementary Fig. S2E).

The direct role of ex-His in activating this pathway within macrophages was then investigated *in vitro*. To establish an optimal cellular model, we first performed quantitative viability assays (CCK-8) to characterize the cytotoxic kinetics. These analyses revealed a progressive, dose- and time-dependent decline in MH-S macrophage survival upon ex-His challenge (Supplementary Fig. S2K, L). Based on this initial screening, a treatment condition of 50 μg/mL for 3 h was strategically selected. At this specific juncture, cell viability exhibited only a moderate decline (retaining ~86% viability), capturing the macrophages precisely within the early “execution phase” of cell death prior to massive terminal lysis. Exposure of the alveolar macrophage cell line MH-S to ex-His under this established condition successfully recapitulated the in vivo pathology, inducing significant membrane permeabilization and cell death (Fig. 2D) via PANoptosis, as confirmed by the activation of its canonical effectors (Fig. 2E). This cell death response was accompanied by the robust upregulation of both IRF1 and NLRC5 protein expression (Fig. 2F, G). This specific *in vitro* validation in murine macrophages, aligned with the human scRNA-seq data, suggests that the protein elevations observed in the whole mouse lung partially originate from this cell population. These findings converge to identify an IRF1-NLRC5 signaling axis in macrophages, activated by extracellular histones, as a critical driver of PANoptosis in the context of lung IRI.

### IRF1 transcriptionally activates NLRC5 to drive PANoptosis

**Fig. 3:**
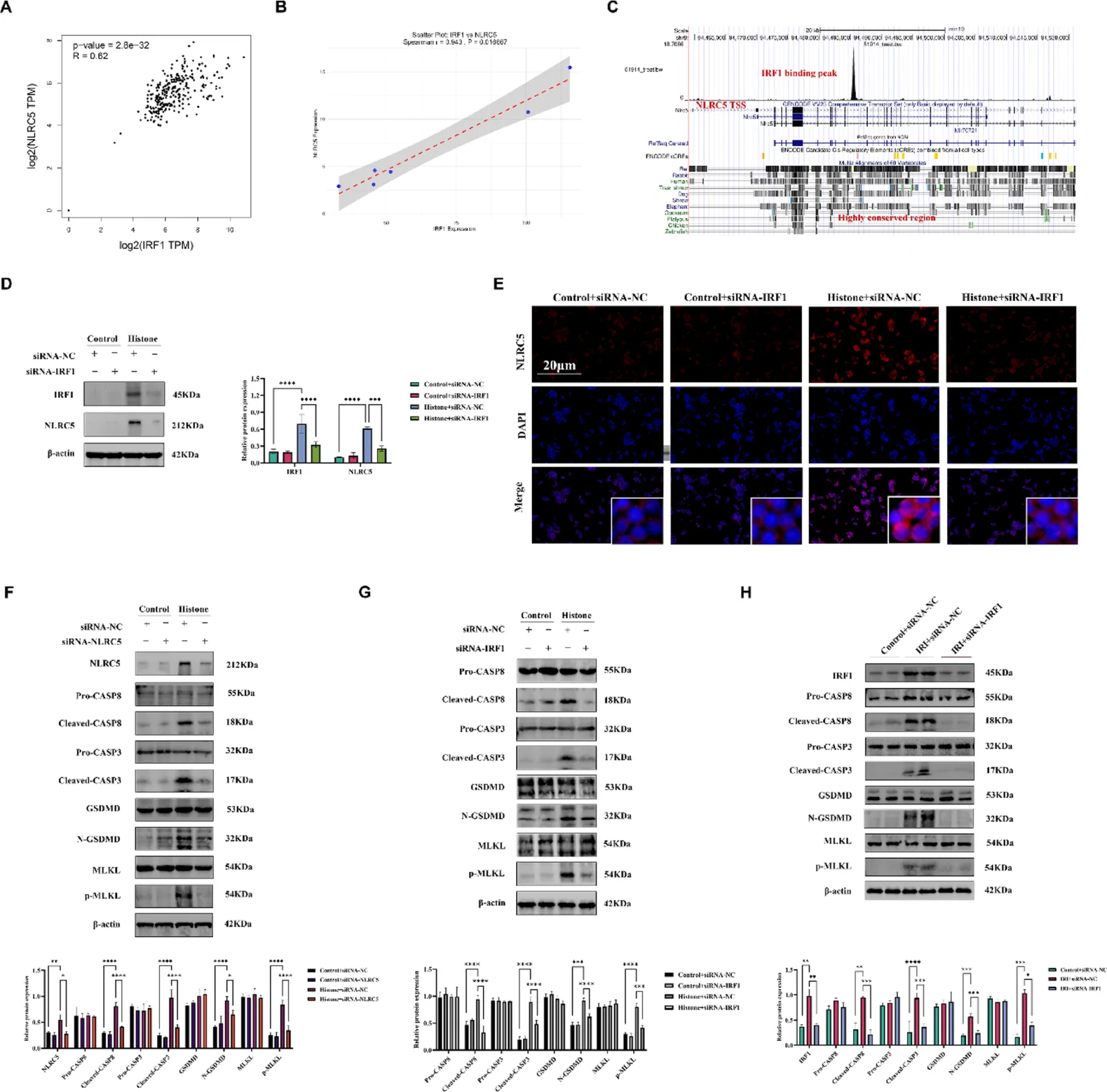
IRF1 Directly Activates NLRC5 Transcription to Drive PANoptosis. **A**, **B** Correlation analysis of IRF1 and NLRC5 mRNA expression in normal human lung tissues from the GEPIA database (**A**) and in murine lung samples from the GSE203238 dataset (**B**). **C** UCSC Genome Browser view of the mouse NLRC5 locus, showing an IRF1 ChIP-seq binding peak located within a highly conserved candidate cis-regulatory element (cCRE) upstream of the transcription start site (TSS). **D**, **E** Validation in MH-S macrophages. Western blot (**D**) and immunofluorescence (**E**) analyses show that siRNA-mediated knockdown of IRF1 abrogates the histone-induced upregulation of NLRC5 protein. In (**E**), NLRC5 is red and nuclei are stained with DAPI (blue). Scale bars, 20 μm and 5 μm (insets). **F**, **G** Western blot analyses demonstrating that knockdown of either NLRC5 (**F**) or IRF1 (**G**) in MH-S cells is sufficient to block the histone-induced activation of PANoptosis effectors (cleavage of CASP8, CASP3, GSDMD, and phosphorylation of MLKL). **H** In vivo validation. Western blot showing that administration of IRF1 siRNA to mice with lung IRI attenuates the activation of downstream PANoptosis markers in lung tissue. Data are presented as mean ± SD (n = 3 per group). Correlation analyses in (**A**) and (**B**) were performed using Pearson correlation. Statistical significance was determined by two-way ANOVA for (**D**–**G**) and one-way ANOVA for (**H**), both followed by Tukey’s multiple comparisons test. *P < 0.05, **P < 0.01, ***P < 0.001, ****P < 0.0001

We investigated the hypothesis that IRF1 functions as a direct transcriptional regulator of NLRC5. Evidence for this relationship first emerged from transcriptomic data, which revealed a significant positive correlation between IRF1 and NLRC5 mRNA expression in both normal human lung tissue (GEPIA database; Fig. 3A) and our murine lung IRI dataset (GSE203238; Fig. 3B). This strong correlative link suggested a direct regulatory relationship, a notion substantiated by analysis of public ChIP-seq data. A prominent IRF1 binding peak was identified directly upstream of the NLRC5 transcription start site (TSS), situated within a candidate cis-regulatory element that is highly conserved across vertebrate species (Fig. 3C).

We then functionally validated this predicted regulatory axis in MH-S macrophages. As anticipated, stimulation with ex-His robustly induced NLRC5 protein expression; however, this effect was significantly attenuated by siRNA-mediated knockdown of IRF1, a finding confirmed by both Western blot and immunofluorescence (Fig. 3D, E). The functional necessity of the complete axis for cell death execution was then examined. Disruption of the axis at either node, achieved by silencing either the downstream effector NLRC5 or the upstream activator IRF1, yielded the same outcome, resulting in a potent suppression of PANoptosis. Specifically, targeted knockdown of NLRC5 or IRF1 was sufficient to block the histone-induced cleavage of CASP8, CASP3, and GSDMD, as well as the phosphorylation of MLKL (Fig. 3F, G). Finally, the physiological relevance of this pathway was confirmed *in vivo*. Administration of IRF1-targeting siRNA to mice subjected to lung IRI significantly attenuated the activation of these PANoptosis markers in the injured lung tissue (Fig. 3H). Crucially, this molecular blockade translated into significant macroscopic protection, as *in vivo* silencing of IRF1 significantly alleviated pulmonary edema (evidenced by a reduced lung W/D ratio) and restored respiratory gas exchange (reflected by an improved PaO₂/FiO₂ ratio) (Supplementary Fig. S2I, J). These findings collectively confirm the role of the IRF1-NLRC5 axis as a critical hierarchical pathway driving PANoptosis and structural lung damage in lung IRI.

### NLRC5 orchestrates PANoptosome assembly to execute PANoptosis

**Fig. 4:**
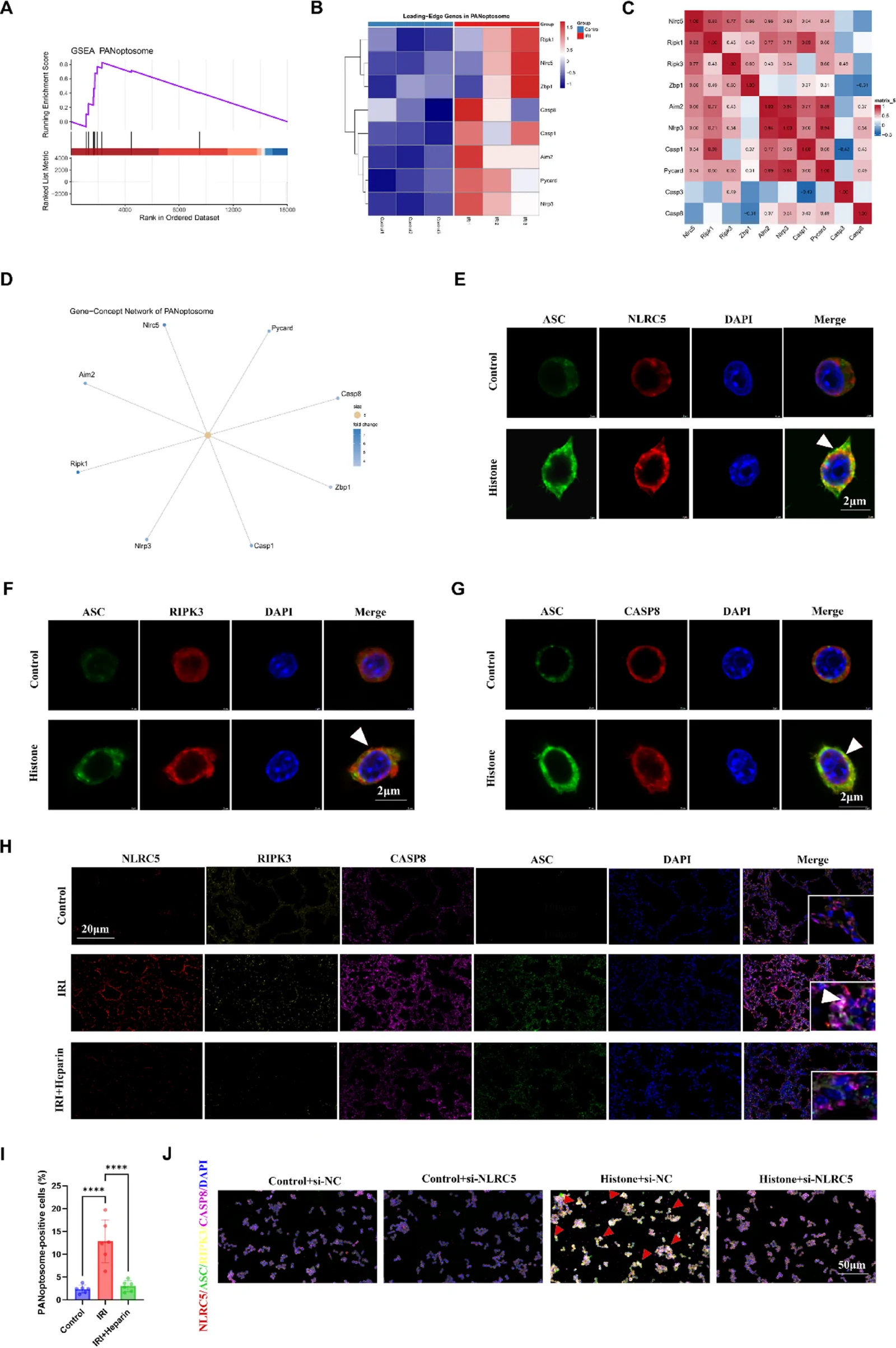
NLRC5 Orchestrates PANoptosome Assembly and PANoptosis Activation. **A** Gene Set Enrichment Analysis (GSEA) plot showing significant enrichment of the PANoptosome gene signature in the lung IRI dataset (GSE203238). **B** Heatmap of leading-edge genes from GSEA, illustrating the coordinated upregulation of core PANoptosome components in IRI. **C** Pairwise correlation matrix of PANoptosome gene expression, indicating their strong co-regulation. **D** Gene-concept network (Cnetplot) visualizing the functional interconnections within the PANoptosome gene set. **E**–**G** Confocal microscopy images of MH-S cells showing histone-induced co-localization of ASC with NLRC5 (E), RIPK3 (F), and CASP8 (G), respectively. Nuclei were counterstained with DAPI. Scale bars, 2 μm. **H** Co-localization of PANoptosome components (NLRC5, RIPK3, ASC, and CASP8) in lung tissue sections under control, IRI, and IRI + heparin conditions, shown by immunofluorescence. Nuclei were counterstained with DAPI. Scale bar, 20 μm. **I** Quantification of the percentage of PANoptosome-positive cells based on the immunofluorescence images in (H). Data are presented as mean ± SD (n = 6 independent fields of view per group). Statistical significance was determined using ordinary one-way ANOVA followed by Tukey’s multiple comparisons test. ****P < 0.0001. **J** Prevention of histone-induced PANoptosome assembly in MH-S cells by siRNA-mediated knockdown of NLRC5, shown by immunofluorescence. Scale bar, 50 μm. All images are representative of at least three independent experiments

The execution of PANoptosis converges on the assembly of the PANoptosome, a multi-protein signaling hub. Gene set enrichment analysis of our IRI dataset (GSE203238) confirmed a substantial upregulation of a curated PANoptosome gene signature (Fig. 4A), with hierarchical clustering revealing a coordinated increase across all core components (Fig. 4B). The strong, positive correlation among these genes at the tissue level was further demonstrated by pairwise correlation analysis and a gene-concept network (Fig. 4C, D), underscoring the global activation of a highly integrated cell death program. Molecular docking simulations predicted a central role for NLRC5 in this architecture, revealing its extensive interaction interfaces with other critical PANoptosome members such as RIPK3, ASC, and CASP8 (Supplementary Fig. S2G, H).

This predicted assembly was then visualized in its cellular context. In response to ex-His, core PANoptosome components coalesced in MH-S macrophages *in vitro*, as demonstrated by the distinct co-localization of the adaptor protein ASC with NLRC5, RIPK3, and CASP8 (Fig. 4E–G). This assembly was robustly recapitulated in vivo. Immunofluorescence imaging of lung tissue from IRI mice revealed co-localization of NLRC5, RIPK3, ASC, and CASP8 into distinct perinuclear specks, a hallmark of PANoptosome formation. In sharp contrast, these proteins remained diffusely distributed in control tissues, and their co-localization was markedly diminished by heparin treatment (Fig. 4H). Quantitative analysis confirmed a robust increase in the percentage of PANoptosome-positive cells following IRI, which was effectively suppressed to near-baseline levels by heparin administration (Fig. 4I). The architectural role of NLRC5 was confirmed by genetic intervention, as siRNA-mediated silencing of NLRC5 was sufficient to prevent the histone-induced assembly of the PANoptosome (Fig. 4J). Collectively, these findings establish NLRC5 as a key organizer of PANoptosome assembly, a process critical for executing the integrated cell death program in lung IRI.

### Mitochondrial oxidative stress and ox-mtDNA release mediate histone-induced IRF1-NLRC5 activation

Having established the IRF1-NLRC5 axis as a fundamental transcriptional pathway driving PANoptosis, we next investigated the upstream molecular mechanisms responsible for its activation. Emerging evidence indicates that mitochondrial dysfunction and oxidative stress are critical initiators of PCD, including PANoptosis, by facilitating the release of mitochondrial damage-associated molecular patterns (mtDAMPs), such as ox-mtDNA [15, 33–35]. Based on this, we hypothesized that ex-His induce mitochondrial damage in AMs, leading to oxidative stress and the release of ox-mtDNA, thereby activating this signaling cascade.

**Fig. 5:**
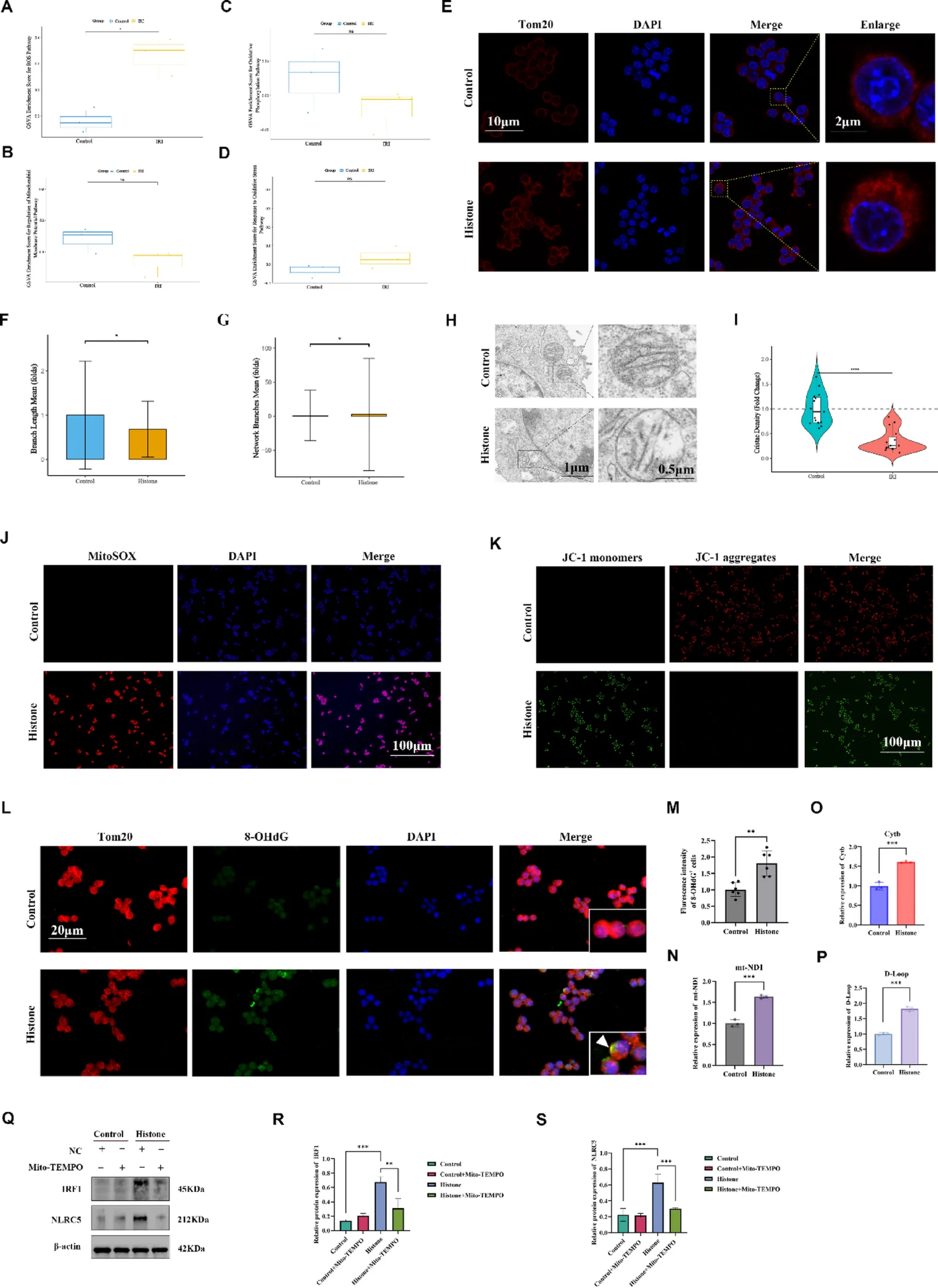
Extracellular histones induce mitochondrial oxidative stress and ox-mtDNA release in macrophages. **A**–**D** GSVA enrichment scores for pathways related to Reactive Oxygen Species (**A**), Mitochondrial Membrane Potential (**B**), Oxidative Phosphorylation (**C**), and Response to Oxidative Stress (**D**) from the GSE203238 dataset. **E**–**G** Mitochondrial network fragmentation in histone-treated MH-S cells. **E** Representative immunofluorescence images of mitochondria (Tom20, red). Scale bars, 10 μm and 2 μm (insets). Quantification of mean branch length (**F**) and network branches (**G**). **H**, **I** Damage to mitochondrial cristae ultrastructure in MH-S cells. **H** Representative transmission electron microscopy (TEM) images. Scale bars, 1 μm and 0.5 μm (insets). **I** Quantification of cristae density. **J** Mitochondrial ROS production in MH-S cells measured by MitoSOX Red staining. Scale bar, 100 μm. **K** Mitochondrial membrane potential in MH-S cells assessed by JC-1 staining. Scale bar, 100 μm. **L**–**P** Mitochondrial DNA damage and cytosolic release. **L**, **M** Immunofluorescence staining for the DNA oxidation marker 8-OHdG (**L**) and its quantification (**M**). Scale bars, 20 μm and 5 μm (insets). (N-P) qRT-PCR quantification of cytosolic mtDNA genes (mt-ND1, Cytb, D-Loop). **Q**–**S** Mito-TEMPO attenuates the histone-induced upregulation of IRF1 and NLRC5 protein expression. (**Q**) Western blot analysis. **R**, **S** Quantification of IRF1 and NLRC5 protein levels. Data are presented as mean ± SD. Statistical analysis was performed by unpaired Student’s *t*-test or two-way ANOVA followed by Tukey’s multiple comparisons test, as appropriate. *P < 0.05, **P < 0.01, ***P < 0.001, ****P < 0.0001

Initial support for this hypothesis came from Gene Set Variation Analysis (GSVA) of the lung IRI dataset (GSE203238). This analysis revealed a significantly enriched pathway signature for reactive oxygen species (ROS), along with impaired oxidative phosphorylation and dysregulated membrane potential (Fig. 5A–D). Collectively, these transcriptomic data suggested a phenotype of mitochondrial oxidative stress in IRI, prompting direct experimental validation.

Experimental validation in MH-S macrophages confirmed that treatment with ex-His induced significant mitochondrial alterations. Immunofluorescence analysis revealed pronounced fragmentation of the mitochondrial network (Fig. 5E–G), consistent with ultrastructural changes observed via transmission electron microscopy, which showed a loss of cristae integrity and mitochondrial swelling (Fig. 5H, I). Functionally, this structural damage was associated with increased mitochondrial ROS production and altered membrane potential (Fig. 5J, K). This oxidative environment consequently led to mitochondrial DNA damage, evidenced by a significant increase in 8-OHdG, a canonical marker for DNA oxidation (Fig. 5L, M). qRT-PCR of cytosolic fractions confirmed that this damaged mtDNA was released into the cytosol, as shown by a significant accumulation of mtDNA genes (mt-ND1, Cytb, D-Loop) (Fig. 5N–P).

The causal link between mitochondrial ROS and the IRF1-NLRC5 axis was then established using Mito-TEMPO, a mitochondria-targeted antioxidant. Pre-treatment of MH-S cells with Mito-TEMPO significantly attenuated the histone-induced upregulation of both IRF1 and NLRC5 protein levels (Fig. 5Q–S). These findings demonstrate that histone-induced mitochondrial oxidative stress and the subsequent release of ox-mtDNA are critical upstream events that activate the IRF1-NLRC5 signaling axis to promote PANoptosis.

### Targeting mitochondrial fission ameliorates histone-induced PANoptosis by inhibiting the IRF1-NLRC5 axis

**Fig. 6:**
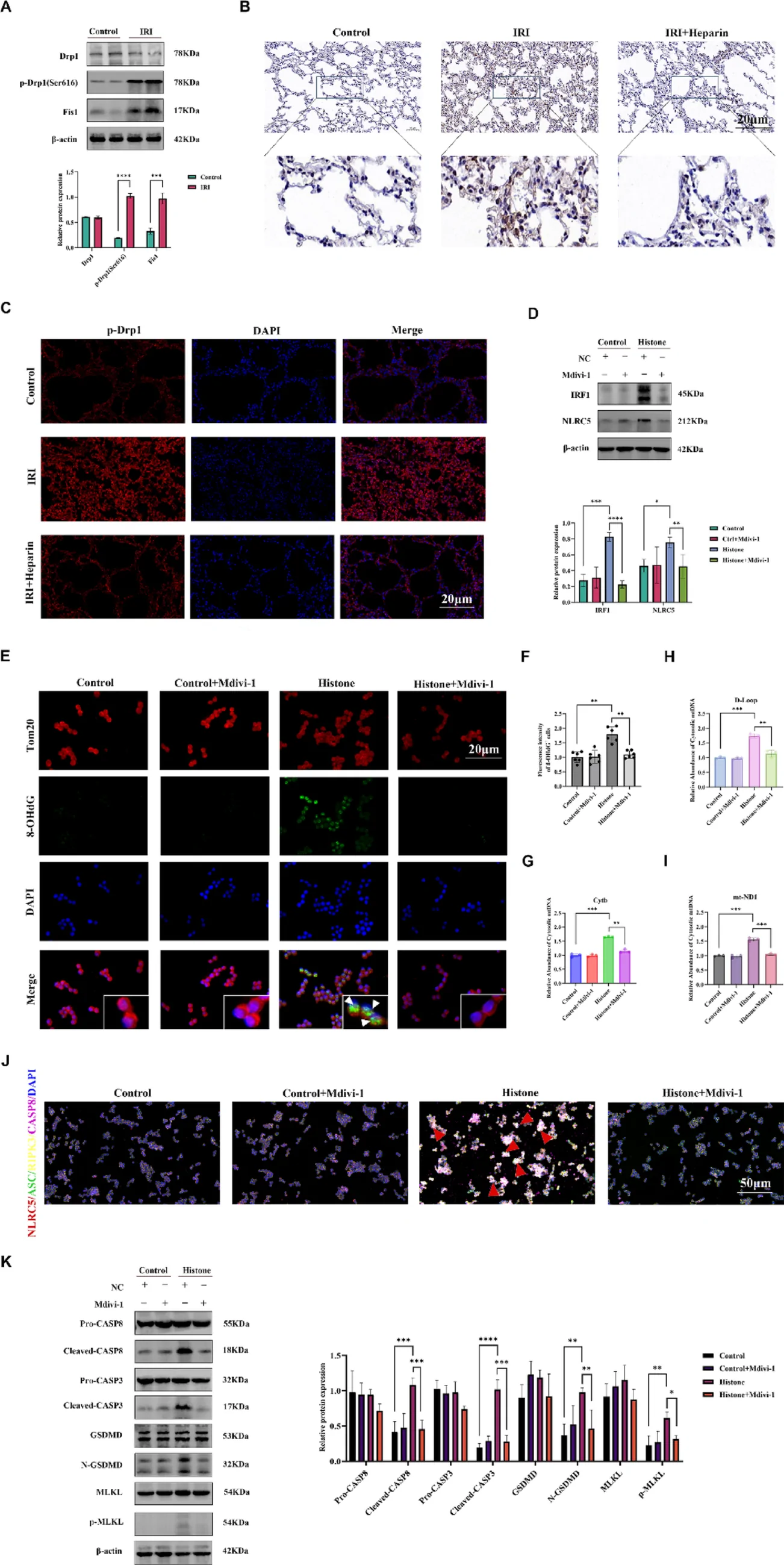
Inhibition of mitochondrial fission ameliorates histone-induced PANoptosis in AMs. **A** Western blot analysis showing increased levels of mitochondrial fission proteins Drp1, p-Drp1, and Fis1 in lung tissues from the IRI group. **B**, **C** Representative immunohistochemistry (**B**) and immunofluorescence (**C**) images confirming the upregulation and distribution of p-Drp1 in lung tissues from IRI mice and the ameliorative effect of heparin. Scale bars, 20 μm. **D** Attenuation of histone-induced IRF1 and NLRC5 upregulation by the Drp1 inhibitor Mdivi-1 in MH-S cells, shown by Western blot. **E**, **F** Mitigation of histone-induced oxidative DNA damage by Mdivi-1 in MH-S cells, shown by immunofluorescence for 8-OHdG (**E**) and its quantification (**F**). Scale bars, 20 μm and 5 μm (insets). **G**–**I** Reduced cytosolic mtDNA release (Cytb, D-Loop, and mt-ND1) in histone-stimulated MH-S cells following Mdivi-1 treatment, quantified by qRT-PCR. **J** Prevention of histone-induced PANoptosome assembly by Mdivi-1 in MH-S cells, shown by immunofluorescence. Scale bar, 50 μm. **K** Suppression of PANoptosis effector protein activation by Mdivi-1 in histone-treated MH-S cells, confirmed by Western blot. Data are presented as mean ± SD. Statistical analysis was performed using unpaired Student’s *t*-test or two-way ANOVA followed by Tukey’s multiple comparisons test, as appropriate. *P < 0.05, **P < 0.01, ***P < 0.001, ****P < 0.0001

The involvement of mitochondrial fission first emerged from our analysis of lung IRI. Western blot analysis of lung tissue revealed significantly elevated levels of the key fission proteins, phosphorylated Drp1 (p-Drp1) and Fis1, in the IRI group (Fig. 6A). This *in vivo* upregulation of p-Drp1 was further confirmed by both immunohistochemistry and immunofluorescence (with the specificity of the fluorescent signal rigorously validated via secondary antibody-only controls, Supplementary Fig. S4) and was partially reversed by heparin pre-treatment (Fig. 6B, C), directly implicating ex-His as inducers of mitochondrial fission.

Given this observation, we investigated whether inhibiting this process could disrupt the downstream pathology. Pharmacological inhibition of Drp1 with Mdivi-1 proved to be effective, significantly attenuating the histone-induced upregulation of both IRF1 and NLRC5 protein expression (Fig. 6D). Consistent with this upstream blockade, Mdivi-1 treatment also prevented histone-induced oxidative DNA damage, as shown by a substantial reduction in 8-OHdG immunofluorescence (Fig. 6E, F). This suppression of oxidative stress was accompanied by a marked reduction in the cytosolic release of mtDNA, with Mdivi-1 treatment significantly mitigating the accumulation of mt-ND1, Cytb, and D-Loop in the cytosol (Fig. 6G–I). These results demonstrate that Drp1-mediated mitochondrial fission is a driver for the release of ox-mtDNA.

This effective blockade of the upstream signaling cascade resulted in the suppression of PANoptosis execution. Mdivi-1 treatment prevented the histone-induced assembly of the PANoptosome, as evidenced by reduced co-localization of its core components (Fig. 6J), and markedly suppressed the activation of downstream PANoptosis effector proteins (Fig. 6K).

### Single-cell analysis delineates an IRF1-driven trajectory toward PANoptosis in human macrophages

**Fig. 7:**
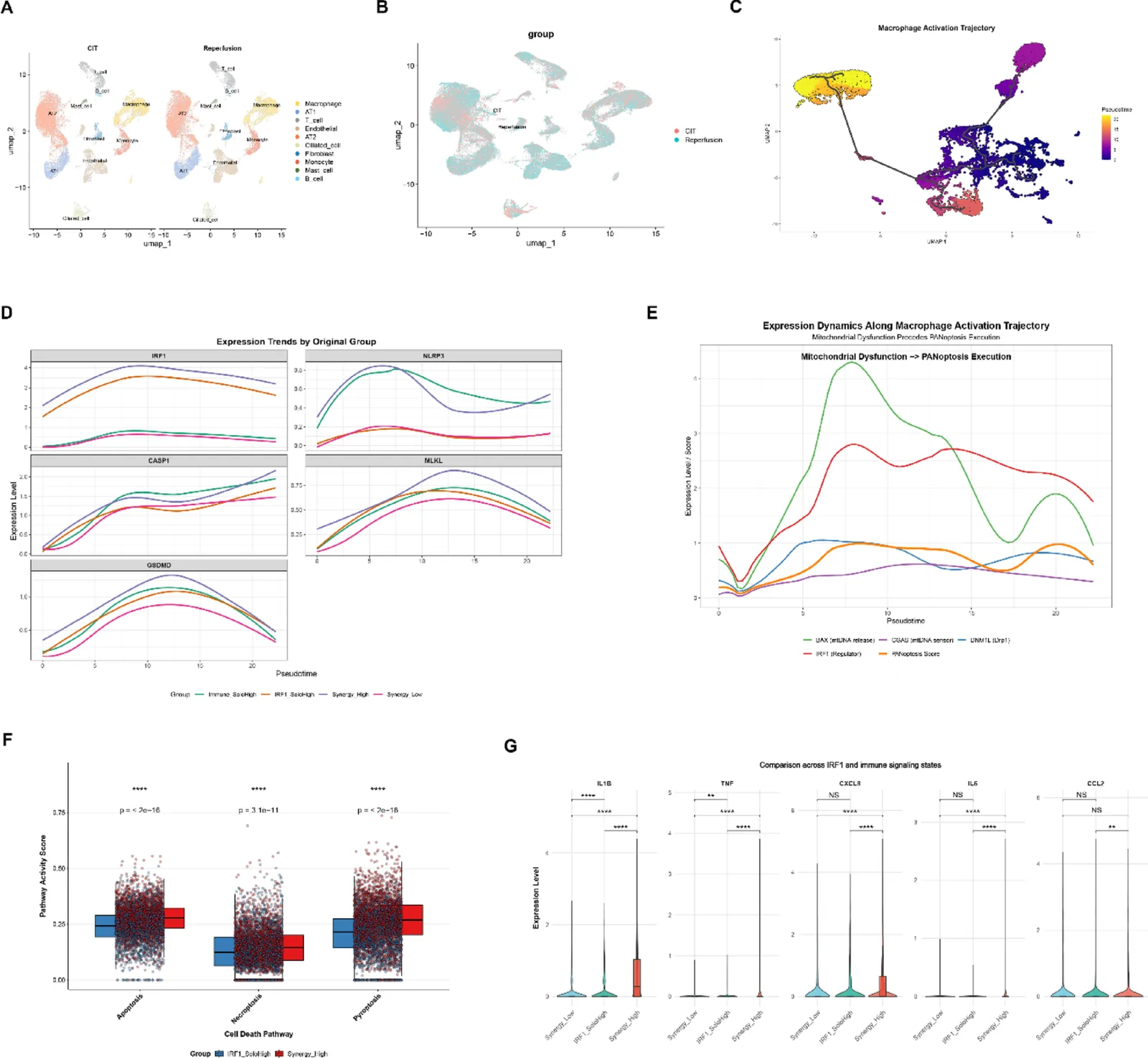
Single-cell analysis delineates an IRF1-Driven Pro-PANoptotic trajectory in human macrophages. **A**, **B** UMAP projections of integrated single-cell transcriptomes, with cells colored by annotated cell type (**A**) and experimental condition (CIT vs. Reperfusion) (**B**). **C** Pseudotime trajectory analysis using Monocle3 on the macrophage population specifically derived from reperfused lung tissues. The trajectory models the dynamic transition of macrophages within the injured environment, revealing a continuous progression from an unactivated baseline (“Resting” state) to a highly activated, pro-PANoptotic endpoint (“Executing” state). **D** Expression kinetics of key PANoptosis-related genes along the pseudotime trajectory. Distinct colored lines represent four functional macrophage subsets functionally stratified by a dual-metric approach (integrating normalized IRF1 expression and a computed immune activity score): Synergy_High (high IRF1, high immune score), IRF1_SoloHigh (high IRF1, low immune score), Immune_SoloHigh (low IRF1, high immune score), and Synergy_Low (low IRF1, low immune score). **E** Temporal ordering of mitochondrial and PANoptosis-related regulators. Line plots showing pseudotime-resolved expression of mitochondrial stress genes (DNM1L, BAX), IRF1, cGAS, and the computed PANoptosis score. **F** Increased composite PANoptosis activity score in IRF1-high macrophage subsets. **G** Violin plots demonstrating heightened expression of proinflammatory cytokines in IRF1-high macrophage subsets

We analyzed single-cell RNA-sequencing data from human lung transplant recipients to define the high-resolution transcriptional landscape of macrophages during lung IRI (Fig. 7A, B). Comprehensive quality control metrics and cell type composition analysis confirmed the robust and uniform sequencing quality of this cohort across all patient samples, ensuring sufficient statistical power for downstream analyses (Supplementary Fig. S5A–D). Notably, cell type composition analysis across experimental groups (Supplementary Fig. S5E) revealed a reduction in the overall proportion of macrophages post-reperfusion. Furthermore, detailed examination of this compartment demonstrated a specific relative depletion of alveolar macrophages compared to other macrophage subpopulations following reperfusion (Supplementary Fig. S3G), a population shift that biologically reflects the execution of this coordinated cell death program.

This physical depletion was accompanied by a distinct transcriptional divergence in the remaining macrophages from post-reperfusion samples compared to those from the cold ischemia time (CIT) group (Supplementary Fig. S3M). The divergence was driven by the significant upregulation of inflammatory and cell death-related programs, including apoptosis, necroptosis, and NOD-like receptor signaling, as confirmed by pathway enrichment analyses (Supplementary Fig. S3A–D, K, L).

To precisely dissect this functional heterogeneity within the post-reperfusion microenvironment, we utilized a dual-metric stratification strategy to categorize these reperfused macrophages into four distinct subsets based on their normalized IRF1 expression and a composite immune activity score (Synergy_Low, Immune_SoloHigh, IRF1_SoloHigh, and Synergy_High). Pseudotime trajectory analysis resolved the activation dynamics within this specific population into a continuous path (Fig. 7C). Notably, analysis of trajectory composition revealed that the computed origin (the “Resting” state) was overwhelmingly dominated by the transcriptionally quiet Synergy_Low subset, whereas the terminal “Executing” state was predominantly composed of the hyperinflammatory Synergy_High population (Supplementary Fig. S3H–J).

Gene expression kinetics along this trajectory revealed a highly coordinated co-regulation and simultaneous upregulation of the IRF1-NLRC5 axis alongside its downstream effectors, including GSDMD, CASP1, and MLKL, particularly culminating in the executing Synergy_High group (Fig. 7D). Deconstructing the temporal sequence of these events showed that mitochondrial stress genes (DNM1L, BAX) were induced early in pseudotime, preceding the robust upregulation of IRF1. In contrast, cGAS transcription remained stable, suggesting its regulation occurs post-translationally. This orchestrated molecular sequence resulted in a progressive increase in the composite PANoptosis score along the trajectory (Fig. 7E).

The central role of IRF1 in driving this pro-death trajectory was further supported by stratifying macrophages based on its expression (Supplementary Fig. S3E, F). IRF1-high macrophage subsets exhibited a significantly increased PANoptosis activity score (Fig. 7F) and displayed heightened expression of proinflammatory cytokines, linking the IRF1 axis to a hyperinflammatory phenotype (Fig. 7G), which indicates a strong positive correlation where highly pro-inflammatory macrophages concurrently over-express PANoptosis mediators.

### The histone-mitochondria-IRF1-NLRC5 axis is markedly activated in lung transplant recipients with PGD

**Fig. 8:**
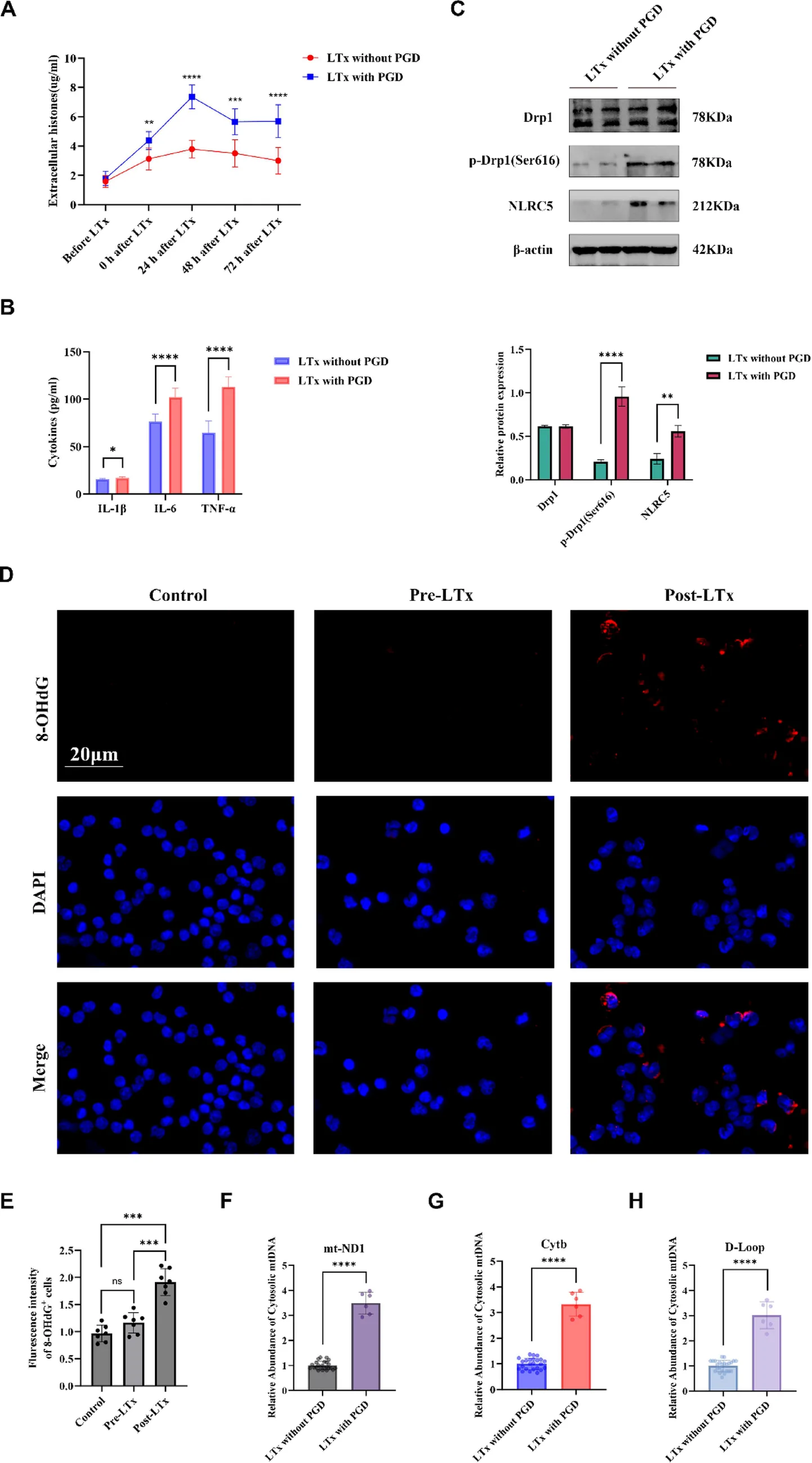
The histone-mitochondria-IRF1-NLRC5 axis is hyperactivated in lung transplant recipients with PGD. **A** Longitudinal measurement of circulating histone levels, showing elevation in PGD patients post-transplantation. **B** Comparison of plasma cytokine levels at 24 h post-transplantation, showing an elevated inflammatory profile in the PGD group. **C** Increased p-Drp1 and NLRC5 protein levels in PBMCs from patients with PGD, shown by Western blot. **D**, **E** Increased oxidized DNA marker 8-OHdG in PBMCs from post-LTx patients, shown by immunofluorescence staining (**D**) and quantification (**E**). Scale bar, 20 μm. **F**–**H** Elevated cytosolic mtDNA fragments (mt-Nd1, Cytb, and D-loop) in PBMCs from PGD patients, confirmed by qRT-PCR. Data are presented as mean ± SEM. Statistical analysis was performed using unpaired Student’s *t*-test or one-way ANOVA followed by Tukey’s multiple comparisons test, as appropriate. *P < 0.05, **P < 0.01, ***P < 0.001, ****P < 0.0001

Our mechanistic findings were validated in a clinical context through the analysis of PBMCs from lung transplant recipients who either developed PGD or did not (non-PGD). Patients with PGD exhibited significantly elevated circulating histone levels, particularly within the first 24 h post-reperfusion (Fig. 8A). This elevation in histones was associated with a hyperinflammatory state, characterized by significantly increased levels of key inflammatory cytokines (Fig. 8B).

This clinical phenotype was directly linked to the core mechanistic pathway we identified. Consistent with our previous report of elevated IRF1 in PGD patients [32], PBMCs from this cohort exhibited significantly higher protein levels of both p-Drp1 and NLRC5 (Fig. 8C), confirming the activation of the entire axis in clinical PGD. This pathway activation was accompanied by mitochondrial oxidative stress, evidenced by a substantial increase in the oxidized DNA marker 8-OHdG in PBMCs post-transplantation (Fig. 8D, E). The resulting oxidative damage was associated with the release of mitochondrial DNA into the cytosol, as qRT-PCR analysis confirmed significantly higher levels of mtDNA fragments (D-loop, Cytb, and mt-Nd1) in PGD patients compared to their non-PGD counterparts (Fig. 8F–H).

## Discussion

**Fig. 9:**
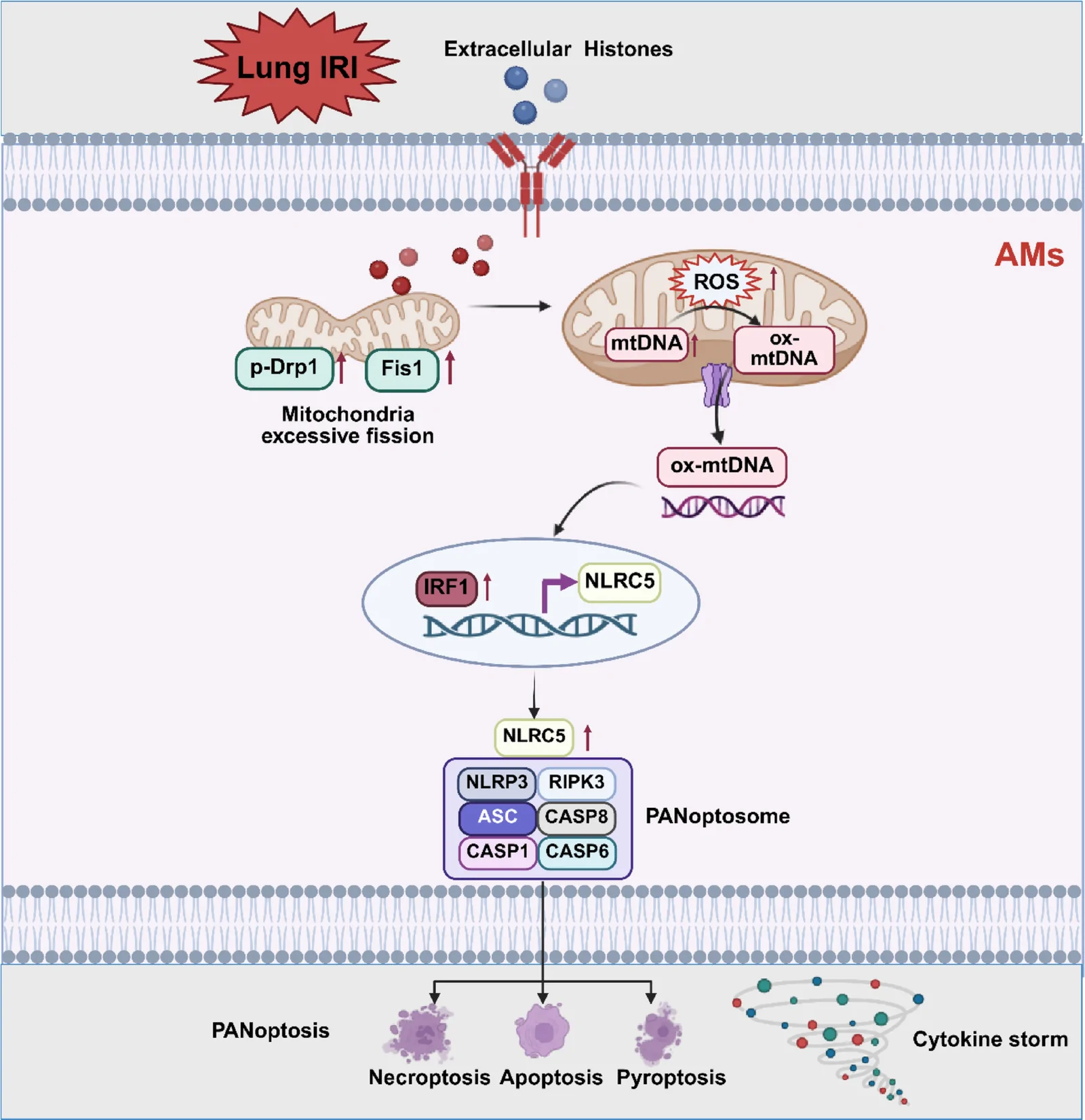
The histone-mitochondria-IRF1-NLRC5 axis drives PANoptosis in Lung IRI. Lung IRI unleashes a pathogenic cascade in AMs, initiated by the release of ex-His. These histones provoke Drp1-mediated mitochondrial fission, leading to excessive ROS production and the cytosolic release of ox-mtDNA. The liberation of this mitochondrial-derived DAMP serves as a critical trigger for the transcriptional upregulation of IRF1, which in turn drives the expression of NLRC5. This axis subsequently programs the cell for PANoptosis by orchestrating the assembly of the PANoptosome complex. This multi-protein platform integrates signals for pyroptosis, apoptosis, and necroptosis, ultimately executing an inflammatory cell death modality that acts as a key effector of tissue destruction in IRI and the progression to PGD

Primary graft dysfunction (PGD) continues to be a significant clinical challenge in LTx, primarily due to a lack of pharmacological interventions grounded in a thorough understanding of its underlying mechanisms. Building upon our prior research, which identified IRF1 as a principal transcriptional regulator of PANoptosis in lung IRI and PGD [32], the present study elucidates the essential upstream signals and downstream molecular frameworks that govern this cell death pathway. We demonstrate that extracellular histones (ex-His), released during initial tissue damage, act as important endogenous danger signals. These histones directly induce mitochondrial fission and oxidative stress, leading to the release of ox-mtDNA into the cytosol. This cytosolic ox-mtDNA in turn activates the IRF1-NLRC5 signaling axis (Fig. 9). Notably, we identify NLRC5 as a non-canonical scaffold for the PANoptosome, thereby assigning an architectural role to this NLR protein in inflammatory cell death that expands its known functions. Our extensive validation strategy, which incorporated pharmacological inhibition and genetic knockdown alongside multi-omics analyses, provides substantial support for this stepwise cascade.

The extracellular release of histones is a recognized event following injurious stimuli. As DAMPs, these ex-His activate the immune system and initiate a self-perpetuating inflammatory cascade through pathways like apoptosis and necroptosis. These histones primarily originate from necroptotic cells and neutrophil extracellular traps (NETs) [36–39]. Their pathogenic effects are well-documented across acute organ injuries such as sepsis and cerebral IRI, where histone neutralization reduces tissue damage [40, 41]. Beyond direct cytotoxicity, the detrimental effects of ex-His are also mediated through their engagement of pattern recognition receptors such as Toll-like receptors (TLRs) 2, 4, and 9 on immune cells, a process that stimulates pro-inflammatory cytokine production and activates downstream pathways like the NLRP3 inflammasome to exacerbate tissue damage [8, 42, 43].

The clinical significance of ex-His is particularly evident within the contexts of critical illness and organ transplantation. In patients with acute respiratory distress syndrome (ARDS), histone H4 has been identified in the BALF while being absent in healthy individuals [44]. Circulating histone levels correlate significantly with the severity and mortality of ARDS [45]. A similar pattern emerges in transplantation scenarios. Post-liver transplantation, histone levels increase and are significantly elevated in patients who develop PGD [46]. Our group has previously reported analogous findings in patients with PGD following LTx, reinforcing this association between ex-His and graft injury [9].

While this body of evidence establishes ex-His as mediators of tissue injury with substantial clinical relevance, the precise intracellular mechanisms by which they induce inflammatory cell death in lung macrophages remain largely undefined. Our study directly addresses this research gap by showing that ex-His serve as upstream drivers initiating Drp1-dependent mitochondrial fragmentation. This process results in increased ROS production and the subsequent release of ox-mtDNA, which then activates the IRF1- and NLRC5-mediated PANoptosis pathway. Systematic pharmacological and genetic inhibition at each step confirmed a cohesive and causally linked pathogenic cascade.

The release of ox-mtDNA from damaged mitochondria provides the critical link to IRF1, a principal transcriptional regulator in the interferon response and a central mediator of inflammatory cell death in organ IRI. Its pathogenic role is supported by evidence from various experimental models. In murine liver IRI, IRF1-deficient mice are protected from injury, exhibiting reduced expression of inflammatory mediators such as TNF-α and iNOS [47]. In the transplantation setting, this protective effect is linked to cell death pathways, as liver grafts from IRF1-deficient donors demonstrate significantly decreased expression of components of the death-receptor pathway and reduced caspase-8 activity, directly linking IRF1 to transplant-associated cell death [48].

IRF1 integrates upstream signaling from PAMPs/DAMPs through pattern recognition receptors (PRRs), leading to cytokine production, interferon induction, and the activation of PCD. PANoptosis is a distinct inflammatory form of PCD, mediated by caspases and receptor-interacting protein kinases and driven by multiprotein PANoptosome complexes that integrate pyroptotic, apoptotic, and necroptotic mechanisms [25]. The interferon signaling pathway, for which IRF1 is a principal transcriptional regulator, serves as a major upstream trigger of PANoptosis [49–51]. Consistent with this role, IRF1 facilitates PANoptosis by inducing the formation of PANoptosome complexes in response to infectious or sterile stimuli and modulates the inflammasome-dependent assembly of PANoptosomes involving caspase-8 and RIPK3.

Our findings extend the existing framework by providing evidence that IRF1 is required for PANoptosis in AMs during lung IRI and plays an important role in PANoptosome formation in PGD. While the role of IRF1 as a transcriptional activator of PANoptosis is well-established, its direct contribution to the physical assembly of the PANoptosome complex remains unclear. This ambiguity prompted our investigation into potential molecular partners. Given recent findings that NOD-like receptors (NLRs) may serve as protein scaffolds in cell death complexes, we hypothesized that NLRC5 could act as a molecular partner, collaborating with IRF1 to facilitate PANoptosome assembly and mediate cell death in PGD.

NLRC5, a member of the NLR family, is traditionally recognized as a transcriptional regulator of the MHC-I genes [19, 52]. However, emerging evidence indicates broader functions for NLRC5 in innate immunity and inflammatory cell death. Its expression is upregulated in lung tissues and AMs following LPS stimulation, suggesting its function as a stress- and inflammation-responsive regulator in the initial stages of acute lung injury (ALI) [53]. NLRC5 has also been identified as an important regulator of innate immune cell death pathways, particularly PANoptosis, in response to PAMPs and DAMPs such as heme or combined cytokine stimuli. NLRC5 interacts with NLRP12 and other components to form cell death complexes, while its expression and ROS production are regulated by TLR signaling and NAD+ metabolism. Underscoring its pathogenic importance, NLRC5 deficiency provides protection in murine models of hemolysis- and inflammation-induced injury, highlighting its potential as a therapeutic target [54].

The pathogenic role of NLRC5 specifically in the lung is further underscored by transcriptomic analysis of human lung grafts, which revealed an upregulation of PANoptosis-related genes during reperfusion, accompanied by the formation of the NLRC5-PANoptosome [55]. Building on these observations, our study provides direct evidence that ex-His, as prominent DAMPs in the transplantation context, drive the assembly of the NLRC5-PANoptosome in AMs. We further establish a direct regulatory link by demonstrating that IRF1 not only facilitates PANoptosis but also transcriptionally regulates NLRC5, thus integrating upstream interferon signaling with this cell death pathway.

The convergence of DAMP signaling on this axis promoted an investigation into mitochondria as a critical intermediary. Our results support this hypothesis, showing that histone exposure induces mitochondrial damage that precedes PANoptosome assembly. We identify this mitochondrial dysfunction, characterized by Drp1-mediated fission and ROS-induced ox-mtDNA release, as the upstream event that activates the IRF1-NLRC5 axis, thereby linking an external DAMP to the specific downstream innate immune machinery that ultimately executes PANoptosis.

Mitochondrial dysfunction is an important mechanism that exacerbates the inflammatory cascade in lung IRI and PGD. During IRI, dysregulated activation of Drp1 induces mitochondrial fission, resulting in structural fragmentation, loss of membrane potential, and the release of mitochondrial DAMPs (mtDAMPs), such as mtDNA into the cytoplasm [56–59]. Importantly, while Drp1-mediated fission is inherently a common and ubiquitous cellular injury response rather than a specialized cell death pathway, its severe hyperactivation in the context of lung IRI acts as a critical pathophysiological bridge. It translates extracellular stress into the abundant release of ox-mtDNA, which subsequently triggers the highly specialized IRF1-NLRC5 downstream cell death program. mtDNA is vulnerable to oxidative stress due to its proximity to the respiratory chain and limited repair capacity. This vulnerability results in the formation of ox-mtDNA, which is more immunostimulatory than its native form and can engage cytosolic sensors to amplify inflammation, such as by binding directly to NLRP3 to trigger inflammasome activation [60]. Consistent with this pathogenic role, our study found significantly elevated ox-mtDNA levels in PBMCs from patients with PGD.

The release of mtDAMPs links mitochondrial damage to the IRF1 and NLRC5 signaling pathways. Our findings establish a pathogenic cascade where ex-His trigger Drp1-mediated mitochondrial fragmentation, leading to the release of ox-mtDNA. This ox-mtDNA in turn activates the IRF1-NLRC5 axis, ultimately promoting PANoptosome assembly and driving PANoptosis in AMs during lung IRI. This mechanism illustrates how mitochondrial stress acts as a critical intermediary, translating extracellular danger signals into the activation of the specific IRF1-NLRC5-driven PANoptotic program, creating a pathogenic feed-forward loop that drives AM PANoptosis in PGD.

The release of these danger signals, notably extracellular histones and cytosolic DNA, plays a crucial role in activating a comprehensive and interconnected network of inflammatory pathways that extend beyond the IRF1-NLRC5 axis. As well-established DAMPs, histones effectively engage surface-level TLRs, particularly TLR2 and TLR4, thereby initiating NF-κB-dependent cytokine production [43, 61]. Simultaneously, the release of DNA, such as ox-mtDNA, acts as a potent activator for various intracellular sensors. Beyond its direct interaction with NLRs, such as NLRP3, to induce inflammasome activation, cytosolic ox-mtDNA serves as a canonical agonist for the cytosolic cyclic GMP-AMP synthase-stimulator of interferon genes (cGAS-STING) DNA-sensing pathway [62, 63]. Activation of the cGAS-STING axis not only elicits robust Type I interferon responses but also facilitates extensive crosstalk with TLRs and inflammasomes. Collectively, the concurrent engagement of TLRs, NLRs, and DNA sensors (cGAS, STING) by histones and DNA establishes a highly synergistic and redundant inflammatory network. This pattern recognition receptor (PRR)-mediated network amplifies the initial DAMP signaling and provides the essential inflammatory priming that ultimately culminates in the robust execution of macrophage PANoptosis [64–66].

While our initial work established this PANoptotic pathway in AMs, the heterogeneous *in vivo* tissue environment, with its influx of infiltrating immune cells, raised questions about the principal cell population driving this process during the peak of injury. Notably, while alveolar epithelial cells are undoubtedly among the most delicate components of the lung and are prime targets of injury, our integrated analysis identifies macrophages as the central orchestrators of this specific cell death program. As demonstrated in our single-cell profiling (Fig. 2C) and consistent with our previous findings [32], the IRF1-NLRC5 axis and the PANoptotic molecular signature are robustly and prominently executed in macrophages compared to epithelial cell clusters (AT1/AT2). Therefore, our findings indicate that the activation of the IRF1-NLRC5-PANoptosis axis is a major pathological event driven predominantly by macrophages during the early acute phase of lung IRI, which justifies our primary focus on this innate immune population. While structural lineages such as endothelial and epithelial cells are directly compromised by excessive extracellular histones and predominantly driven toward classical caspase-3-dependent apoptosis or passive necrosis [67, 68], macrophages utilize these same DAMPs to trigger specialized inflammatory cell death. Importantly, this mechanistic paradigm extends beyond lung transplantation and is highly generalizable; analogous pathways driving macrophage/monocyte pyroptosis or PANoptosis have been established as commonly conserved fundamental mechanisms propagating sterile inflammation in other acute critical illnesses, including sepsis-induced acute lung injury and acute kidney injury [69–71]. To further dissect the dynamic nature of this macrophage-driven response, our single-cell transcriptomic analysis reconceptualized macrophage PANoptosis from a static endpoint to a dynamic, programmable cell fate trajectory. By reconstructing a continuum that progresses from quiescence through intermediate activation to terminal execution, we establish PANoptosis as a stepwise biological program governed by an activation threshold.

This trajectory analysis revealed the temporal sequence of the pathogenic cascade, establishing a model where mitochondrial stress is the trigger and IRF1 is the central integrator. Our data show that transcriptional signatures of mitochondrial stress and fission (DNM1L, BAX) precede IRF1 activation. The early and sustained induction of IRF1 along the trajectory confirms its role as a principal transcriptional integrator, which in turn orchestrates the sequential activation of inflammasome (NLRP3), pyroptotic (CASP1, GSDMD), and necroptotic (MLKL) pathways. It is important to acknowledge that while our transcriptomic data (RNA-seq and scRNA-seq) indicate cellular priming and susceptibility to PANoptosis, actual biological execution relies on post-translational assembly. Therefore, morphological and biochemical validations, as performed in our study, remain indispensable for predicting precise PANoptosome activity.

Beyond the temporal sequence, our single-cell data also unraveled the cellular heterogeneity underpinning this process *in vivo*. We identified a distinct macrophage subpopulation (Synergy_High) characterized by co-activation of the IRF1-mediated inflammatory pathway and an elevated immune status. The emergence of this specialized cell state, primed to execute inflammatory cell death and activate the adaptive immune system, likely contributes to the tissue damage observed in IRI.

The clinical relevance of the histone-mitochondria-IRF1-NLRC5 axis is underscored by our validation in PGD patient samples, which revealed elevated levels of circulating histones, ox-mtDNA, and activated IRF1-NLRC5 signaling in PBMCs. These findings suggest their potential utility as early biomarkers for risk stratification. This clinical correlation validates the pathway as a potential therapeutic target. Consequently, therapeutic interventions targeting this cascade, such as neutralizing ex-His, inhibiting Drp1-mediated mitochondrial fission, or disrupting IRF1-NLRC5 signaling, may represent promising strategies to mitigate early graft injury. In this context, we specifically utilized N-acetylheparin as a histone-neutralizing agent. Distinct from native, unfractionated heparin, N-acetylheparin is a chemically modified, non-anticoagulant derivative that retains high affinity for cationic histones while avoiding systemic bleeding risks and profound eNOS-mediated hemodynamic alterations [72]. Our laboratory has consistently validated the efficacy and safety of N-acetylheparin in lung IRI models, confirming its role as a specialized tool for scavenging DAMPs rather than modulating systemic rheology [73]. While we recognize that systemic microcirculation plays a critical role in IRI, our finding that N-acetylheparin directly suppresses PANoptosis in isolated MH-S macrophages—independent of vascular and hemodynamic factors—confirms that its primary protective mechanism is mediated through the direct inhibition of the macrophage-intrinsic PANoptotic program. Nevertheless, we acknowledge that while N-acetylheparin circumvents systemic anticoagulant effects, it remains a polyanion that may non-specifically interact with other positively charged proteins *in vivo*. Therefore, to conclusively isolate histone-specific pathogenic mechanisms, future studies employing more selective approaches, such as specific neutralizing antibodies or histone-binding peptides, are ultimately warranted.

In addition to the previously discussed upstream interventions, directly targeting downstream transcription factors such as IRF1 presents significant clinical challenges due to their intracellular localization and the absence of conventional drug-binding sites. Consequently, future translational research should also consider exploring druggable parallel pathways that can amplify this signaling cascade. For example, IRF1 is known to upregulate Thrombospondin-1 (TSP1), which, upon secretion, interacts with the CD47 receptor. The TSP1-CD47 interaction has been demonstrated to exacerbate ischemia–reperfusion injury in various transplant models [74, 75] and to promote significant mitochondrial dysfunction [76]. This signaling axis likely facilitates further translocation of Drp1 to the mitochondria, establishing a pathogenic positive feedback loop (Drp1-IRF1-TSP1-CD47-Drp1) that perpetuates macrophage damage and PANoptosis. Given that small-molecule inhibitors and blocking antibodies targeting the TSP1-CD47 axis are currently under development and have shown efficacy in mitigating transplant-induced ischemia–reperfusion injury, targeting this specific interaction represents a highly promising therapeutic strategy to disrupt this pathogenic cycle in PGD.

Our research elucidates a fundamental pathogenic pathway but also underscores several limitations and areas for future investigation. Firstly, although our single-cell transcriptomic analysis and *in vitro* experiments indicate a prominent involvement of the IRF1-NLRC5-PANoptosis axis in macrophages, we observed that other immune cell populations, such as T cells and B cells, may also express these genes. Additionally, the pseudotime trajectory analysis offers inferential predictions of cell fate rather than direct temporal measurements. Furthermore, our current mechanistic assertions in vivo are predominantly based on pharmacological inhibition and siRNA-mediated knockdown. The absence of macrophage-specific conditional knockout mouse models (e.g., *Lyz2*-Cre) in this study precludes a direct demonstration of the absolute cell-specificity of this pathway *in vivo*. Future research employing such targeted genetic models is essential to definitively confirm the isolated contribution of macrophage-derived PANoptosis to lung pathology.

Several mechanistic and translational questions also remain. The precise mechanism by which ex-His are detected to induce mitochondrial distress remains to be fully elucidated. Additionally, although PBMCs offer a convenient proxy for assessing immune activation, they may not accurately represent the dynamics of tissue-resident AMs within the pulmonary environment. Furthermore, because our *in vivo* studies were exclusively conducted utilizing young male mice to minimize baseline biological variance, subsequent studies incorporating female and aged murine cohorts are needed to fully ascertain the pathophysiological generalizability of the IRF1-NLRC5 axis across different sex and age groups. Moreover, regarding the physiological assessment of the lung graft, we relied on gold-standard surrogate indicators such as the wet-to-dry (W/D) ratio and arterial oxygenation index. The inability to directly measure dynamic pulmonary mechanics (e.g., lung capacity and compliance) *in vivo* due to equipment constraints represents a technical limitation; future investigations employing specialized micro-ventilator systems will be highly valuable to comprehensively evaluate functional recovery. Lastly, the relatively small patient cohort in our study necessitates larger-scale validation to confirm the clinical utility of ox-mtDNA and the IRF1-NLRC5 pathway as predictive biomarkers.

## Conclusion

This study defines a mechanistic pathway that connects mitochondrial dysfunction to innate immune activation in PGD. Ex-His trigger Drp1-mediated mitochondrial fission, leading to the release of ox-mtDNA. This cytosolic DAMP subsequently activates the IRF1-NLRC5 axis, thereby promoting the assembly of the PANoptosome and inducing PANoptosis in AMs. The validation of this pathway in PGD patients underscores its potential as a therapeutic target and supports the development of circulating ox-mtDNA as a prognostic biomarker to guide clinical decision-making in LTx.

## Supplementary Information

Below is the link to the electronic supplementary material.

Supplementary Material 1.

Supplementary Material 2.

Supplementary Material 3.

Supplementary Material 4.

Supplementary Material 5.

Supplementary Material 6.

Supplementary Material 7.

## Data Availability

The data that support the findings of this study are not openly available and are available from the corresponding author upon reasonable request.

## Abbreviations

IRI: Ischemia-reperfusion injury
LTx: Lung transplantation
PGD: Primary graft dysfunction
DAMPs: Damage-associated molecular patterns
Drp1: Dynamin-related protein 1
ex-His: Extracellular histones
mtDNA: Mitochondrial DNA
ROS: Reactive oxygen species
ox-mtDNA: Oxidized mitochondrial DNA
IRF1: Interferon Regulatory Factor 1
PAMPs: Pathogen-associated molecular patterns
NLRC5: NOD-like receptor family, CARD domain containing 5
NLR: NOD-like receptor
PCD: Programmed cell death
AMs: Alveolar macrophages
siRNA: Small interfering RNA
PBMCs: Peripheral blood mononuclear cells
W/D: Wet-to-dry
BALF: Bronchoalveolar lavage fluid
FBS: Fetal bovine serum
DMSO: Dimethyl sulfoxide
H&E: Hematoxylin and eosin
IF: Immunofluorescence
MiNA: Mitochondrial Network Analysis
SDS-PAGE: Sodium dodecyl sulfate–polyacrylamide gel electrophoresis
PVDF: Polyvinylidene fluoride
ECL: Enhanced chemiluminescence
IL-6: Interleukin-6
IL-1β: Interleukin-1β
TNF-α: Tumor necrosis factor-alpha
ELISA: Enzyme-linked immunosorbent assay
qPCR: Quantitative real-time PCR
ΔΨm: Mitochondrial membrane potential
GEO: Gene Expression Omnibus
DEGs: Differentially expressed genes
GO: Enriched Gene Ontology
KEGG: Kyoto Encyclopedia of Genes and Genomes
GSEA: Gene Set Enrichment Analysis
GSVA: Gene Set Variation Analysis
PCA: Principal component analysis
UMAP: Uniform manifold approximation and projection
SD: Standard deviation
ANOVA: One-way analysis of variance
PaO₂: Partial pressure of oxygen
mtDAMPs: Mitochondrial damage-associated molecular patterns
CIT: Cold ischemia time
OXPHOS: Oxidative Phosphorylation
8-OHdG: 8-Hydroxy-2’-deoxyguanosine
NETs: Neutrophil extracellular traps
TLRs: Toll-like receptors
PRRs: Pattern recognition receptors
ALI: Acute lung injury
OPOs: Organ Procurement Organizations
COTRS: China Organ Transplant Response System
DBD: Donors after brain death
DCD: Donation after circulatory death
