# Supplementary material for: IRF1-mediated sensing of oxidized mitochondrial DNA drives macrophage PANoptosis in lung ischemia–reperfusion injury
Source: Apoptosis. 2026 Jul 25;31(8):198. doi: 10.1007/s10495-026-02401-3 (PMC13401555; doi:10.1007/s10495-026-02401-3)

Source data for Fig. 1G

Pro-CASP8:

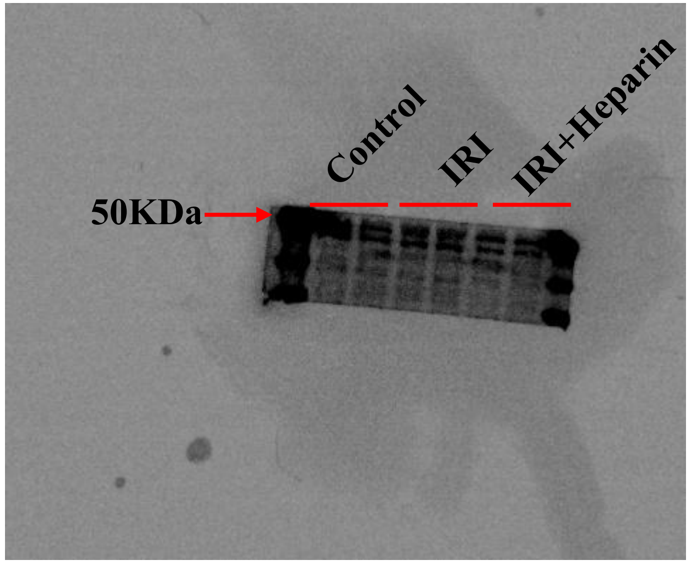

Pro-CASP3:

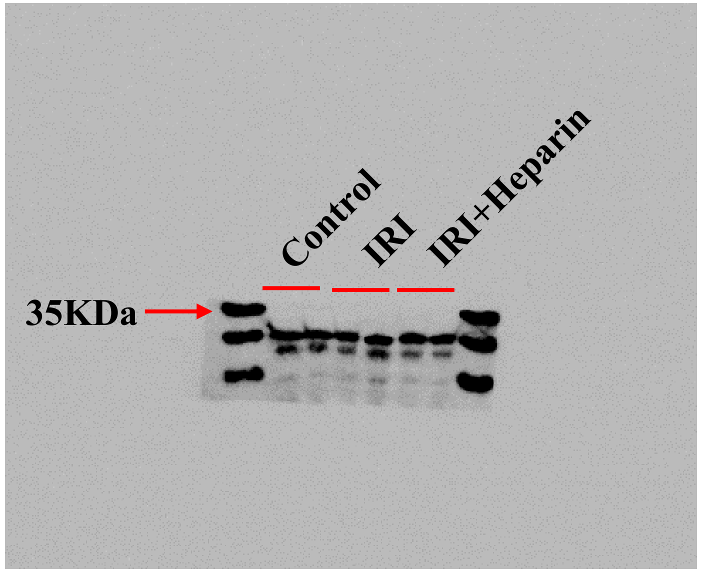

GSDMD:

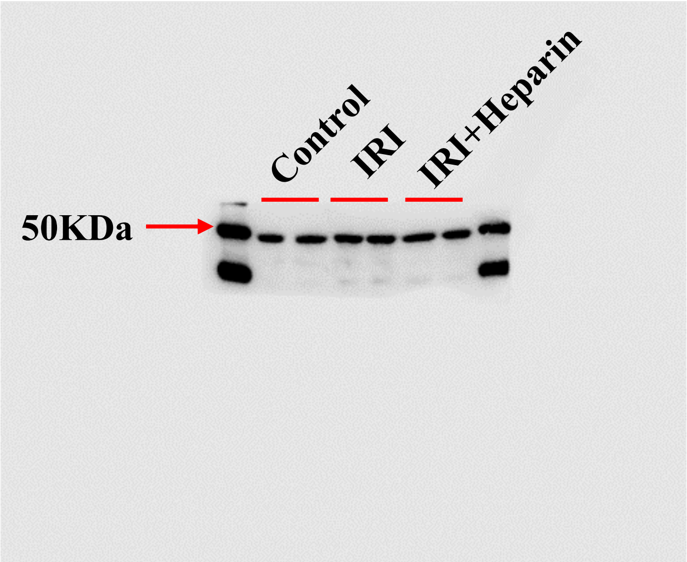

Cleaved-CASP8:

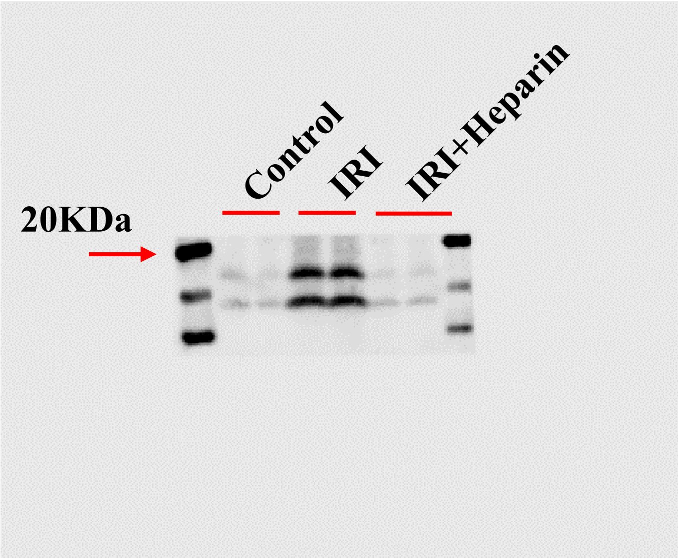

Cleaved-CASP3:

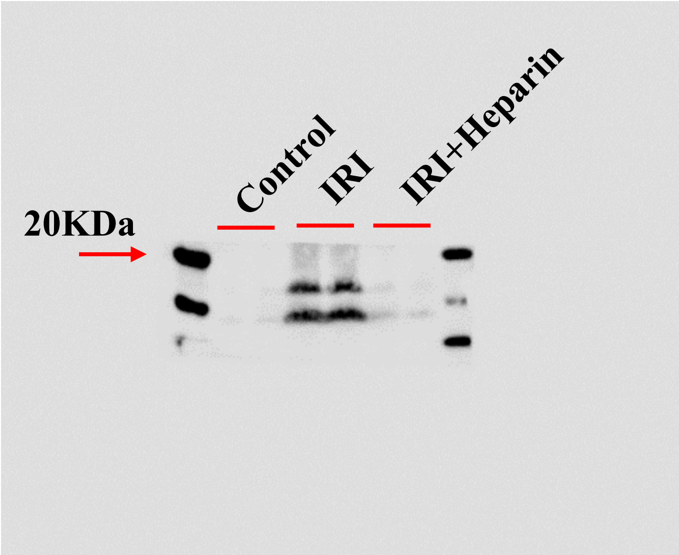

N-GSDMD:

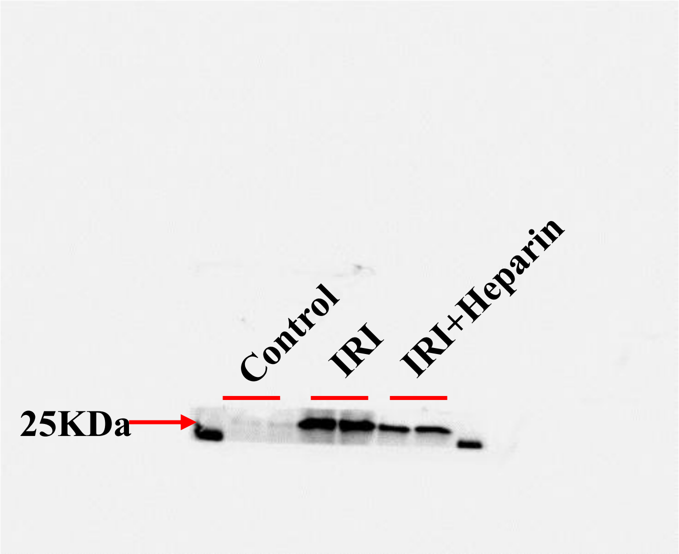

Source data for Fig. 1G

MLKL:

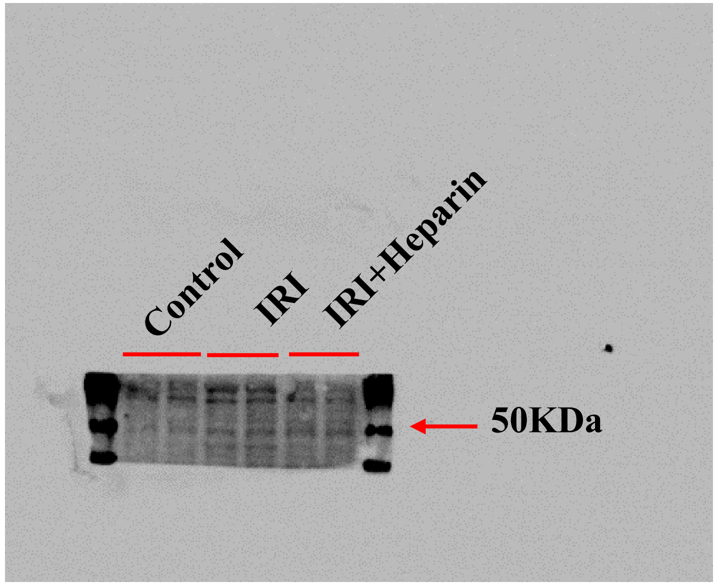

β-actin:

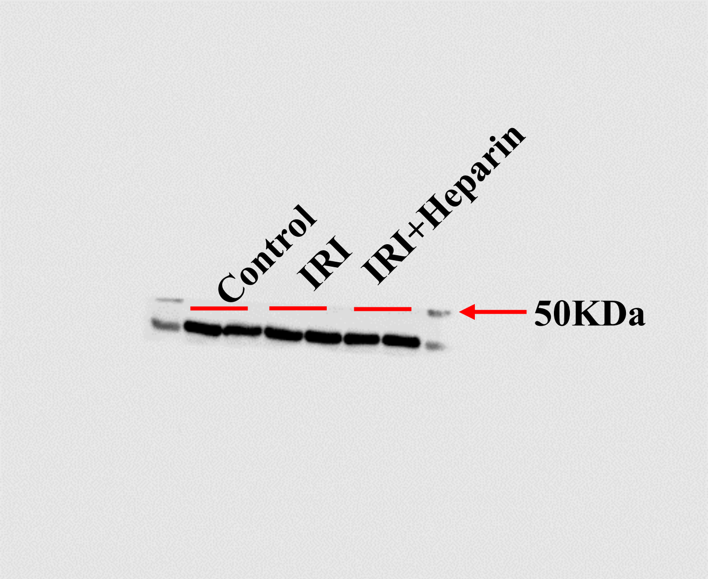

p-MLKL:

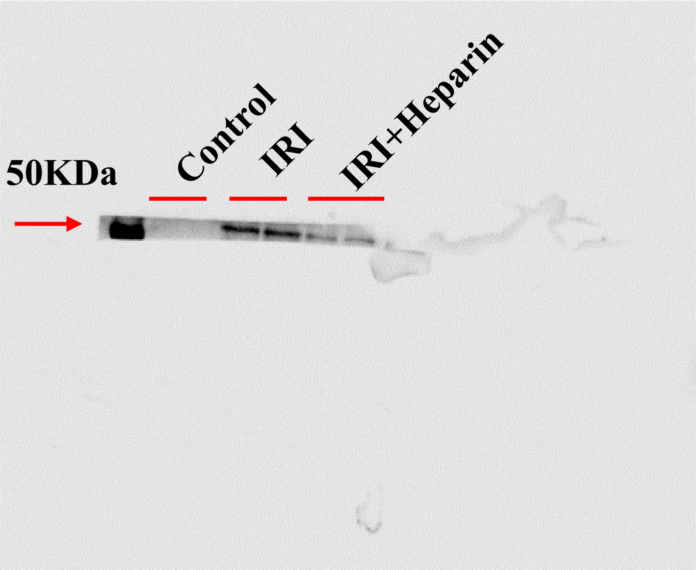

Source data for Fig. 2E

Pro-CASP8:

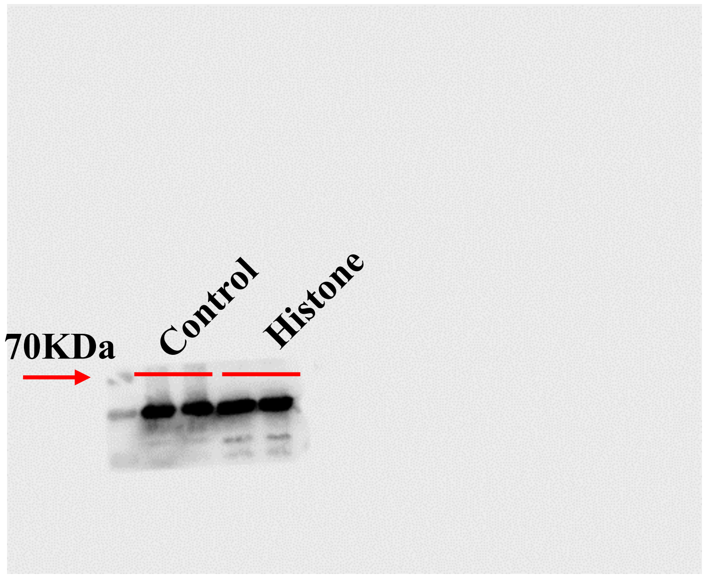

Pro-CASP3:

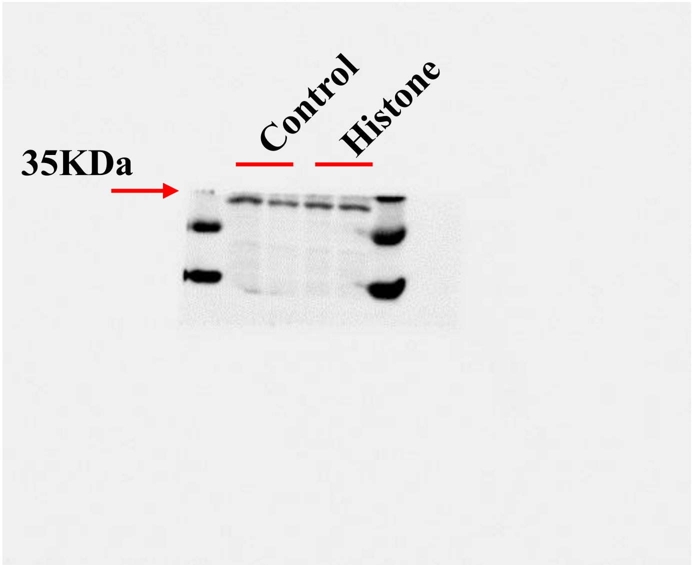

GSDMD:

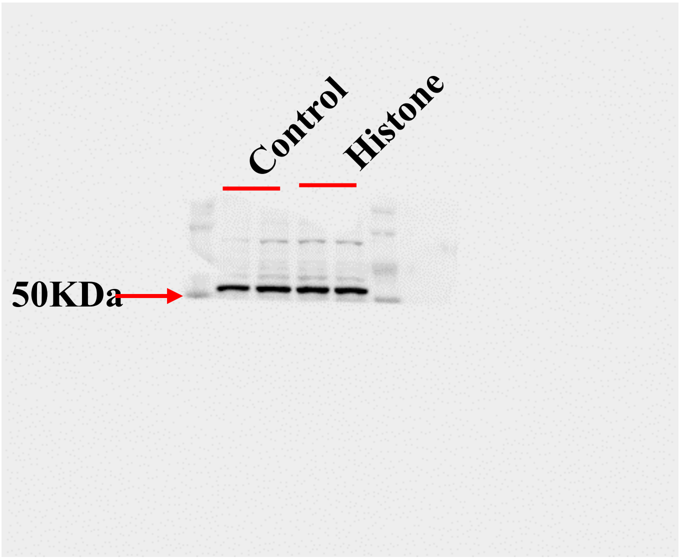

Cleaved-CASP8:

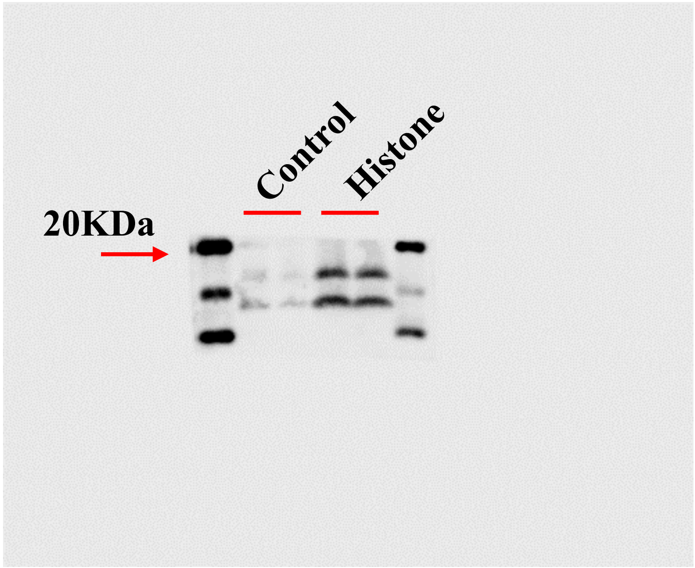

Cleaved-CASP3:

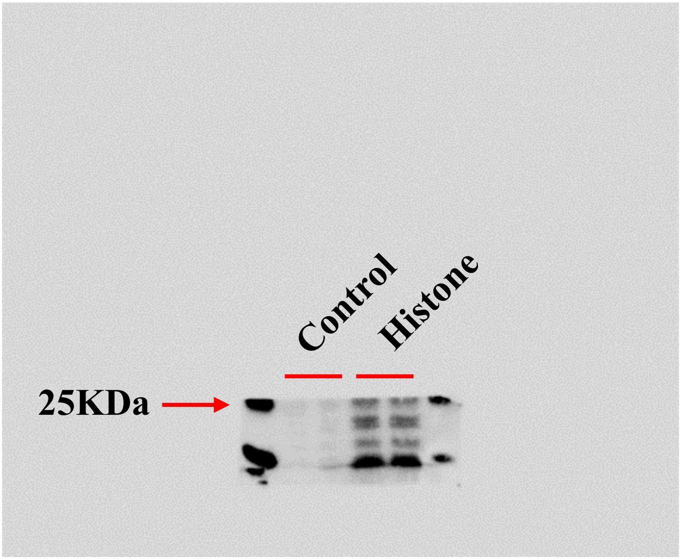

N-GSDMD:

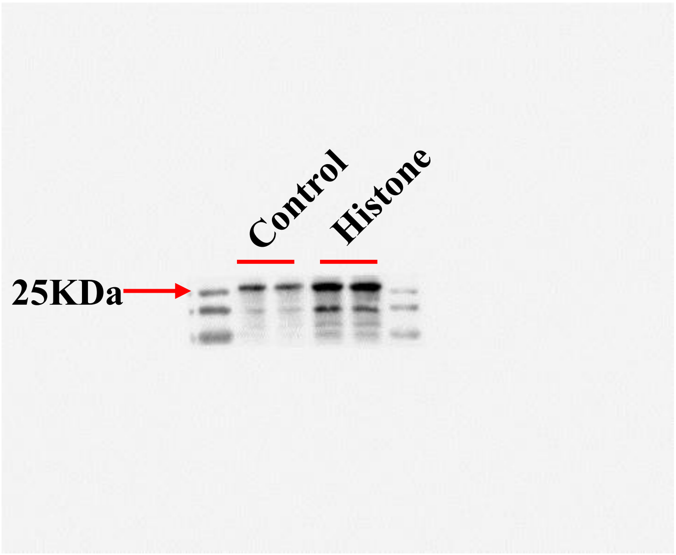

Source data for Fig. 2E

MLKL:

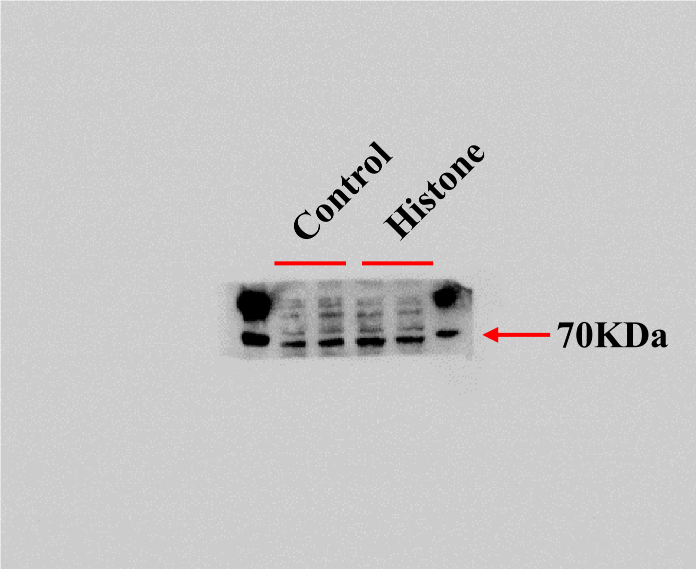

β-actin:

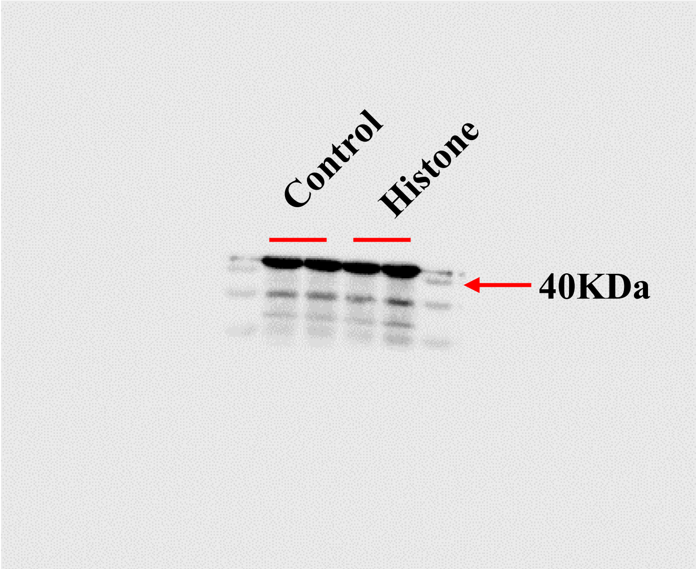

p-MLKL:

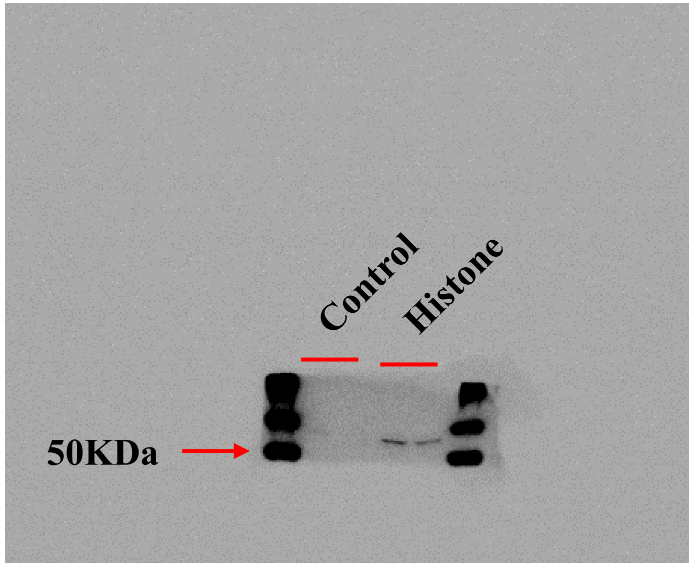

Source data for Fig. 2F

IRF1:

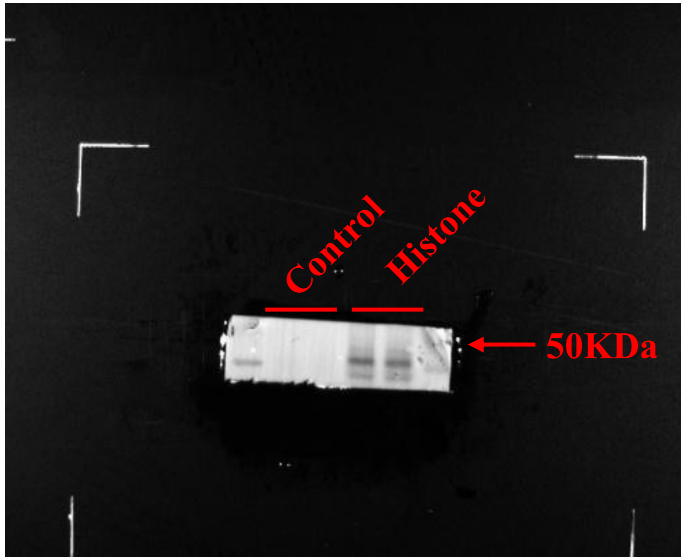

β-actin:

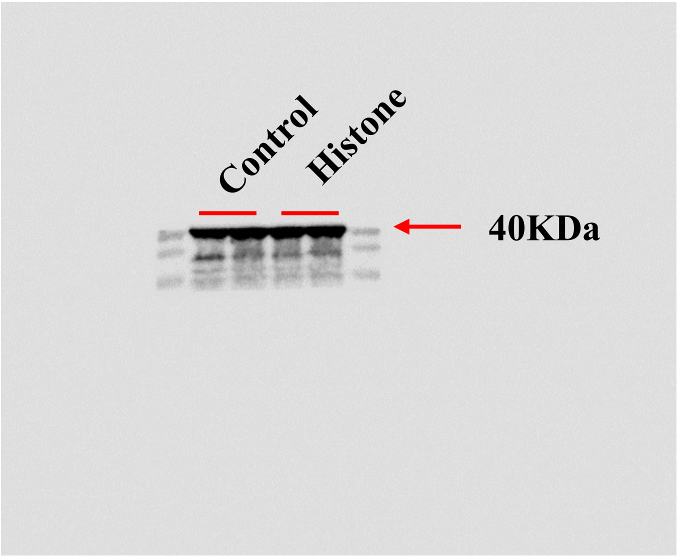

Source data for Fig. 2G

NLRC5:

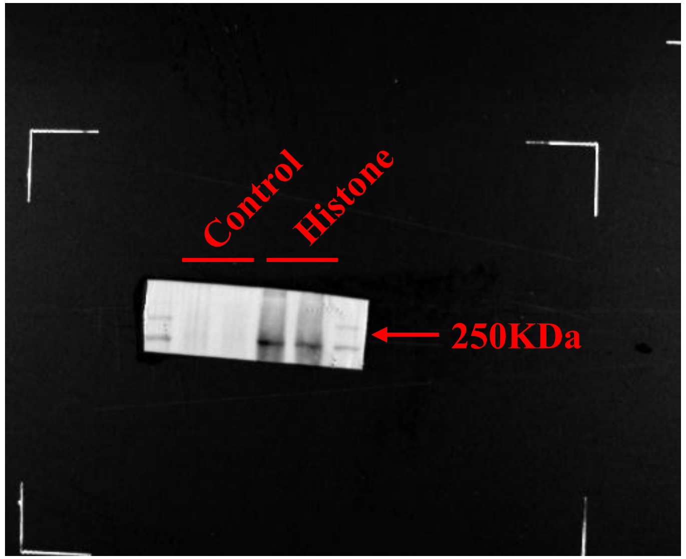

β-actin:

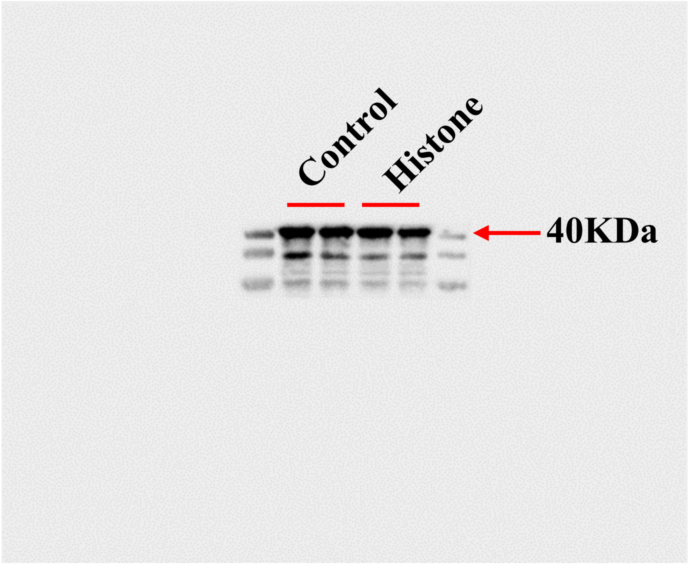

Source data for Supplementary Fig. S2C

IRF1:

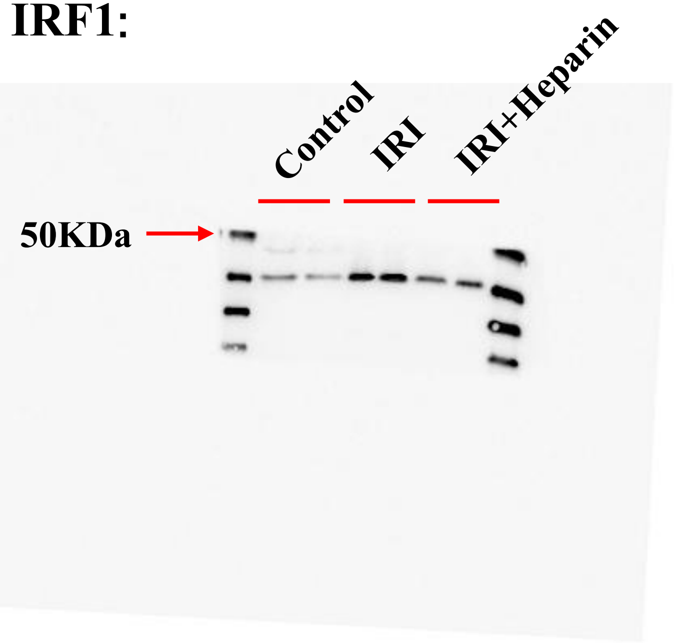

β-actin:

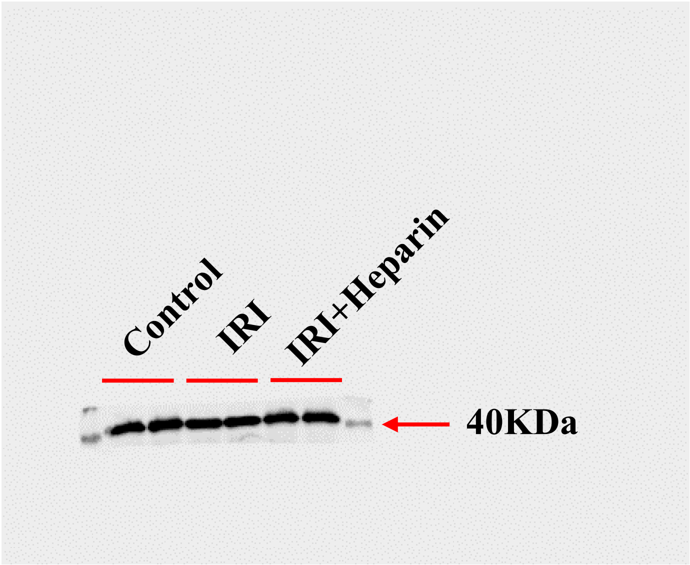

Source data for Supplementary Fig. S2D

NLRC5:

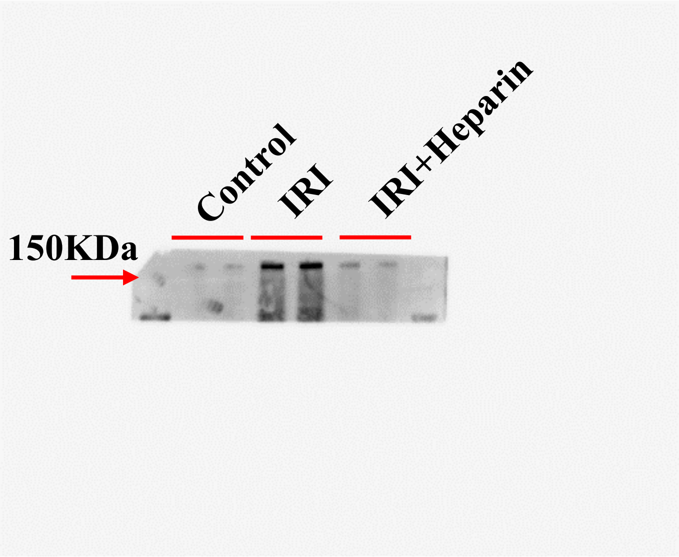

β-actin:

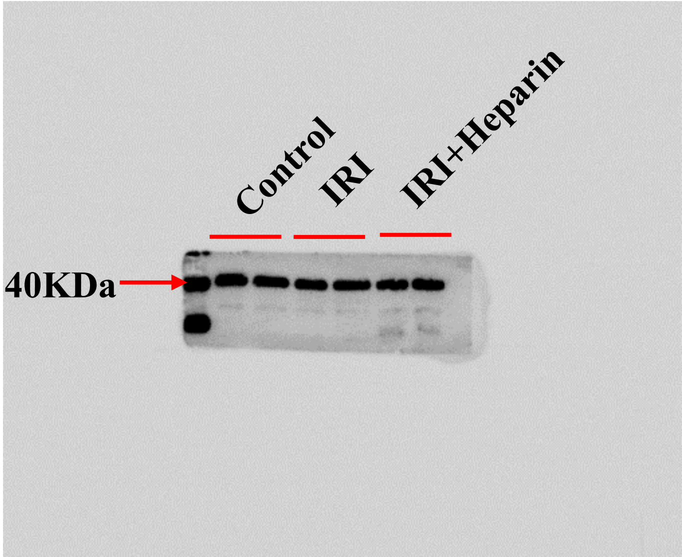

Source data for Fig. 3D

IRF1:

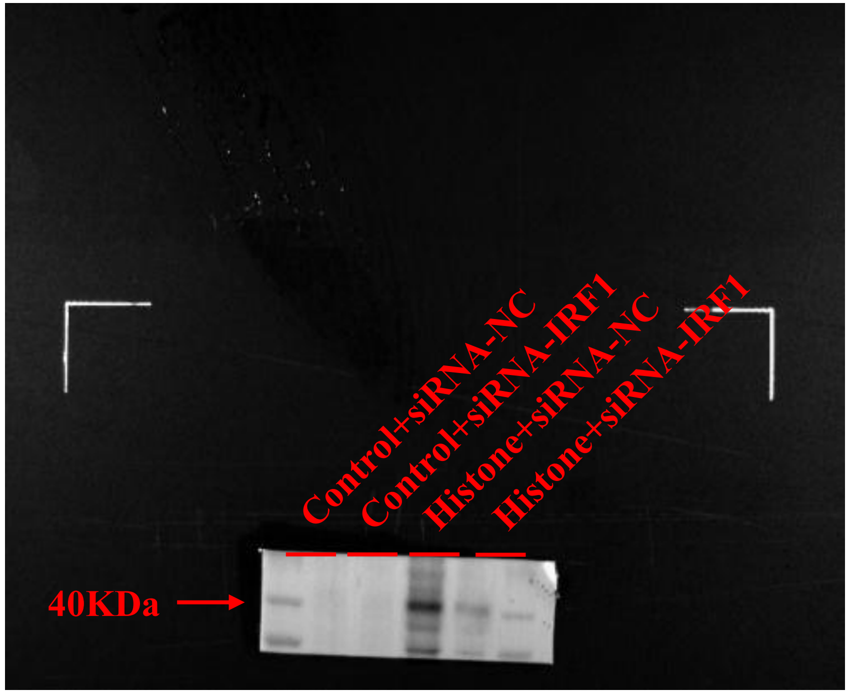

NLRC5:

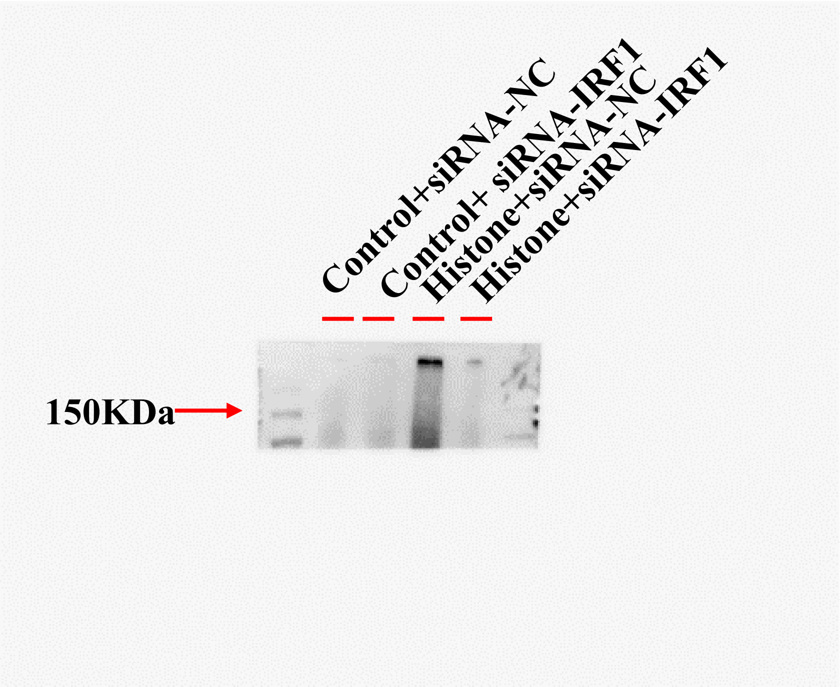

β-actin:

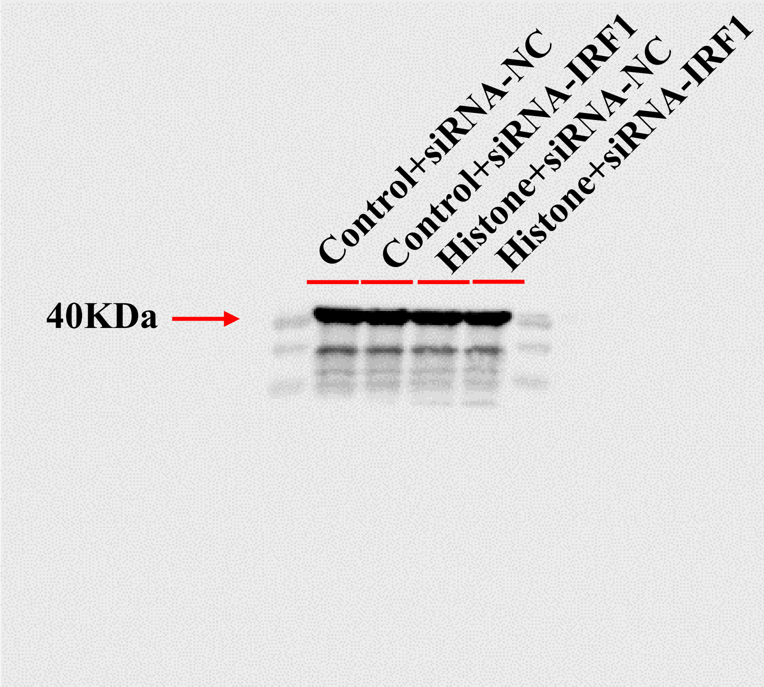

Source data for Fig. 3F

NLRC5:

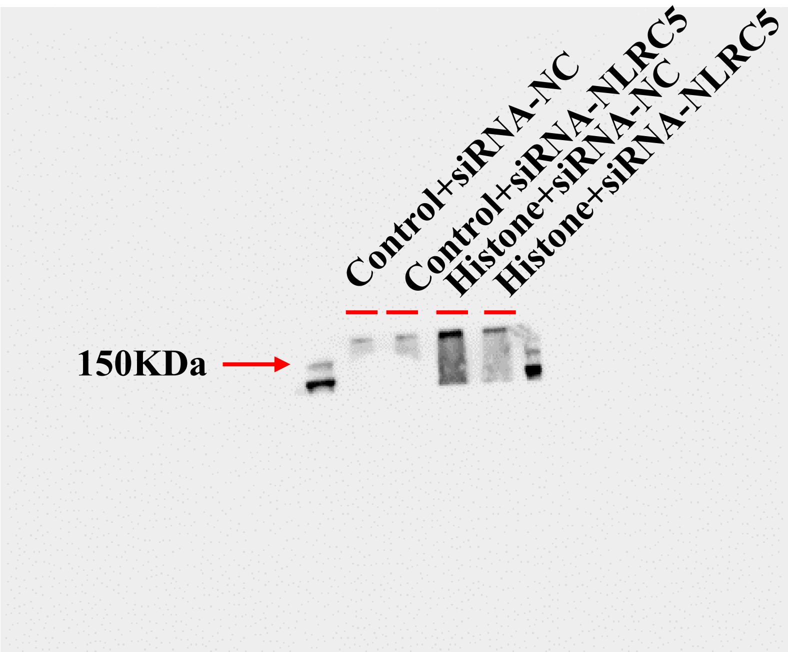

Pro-CASP8:

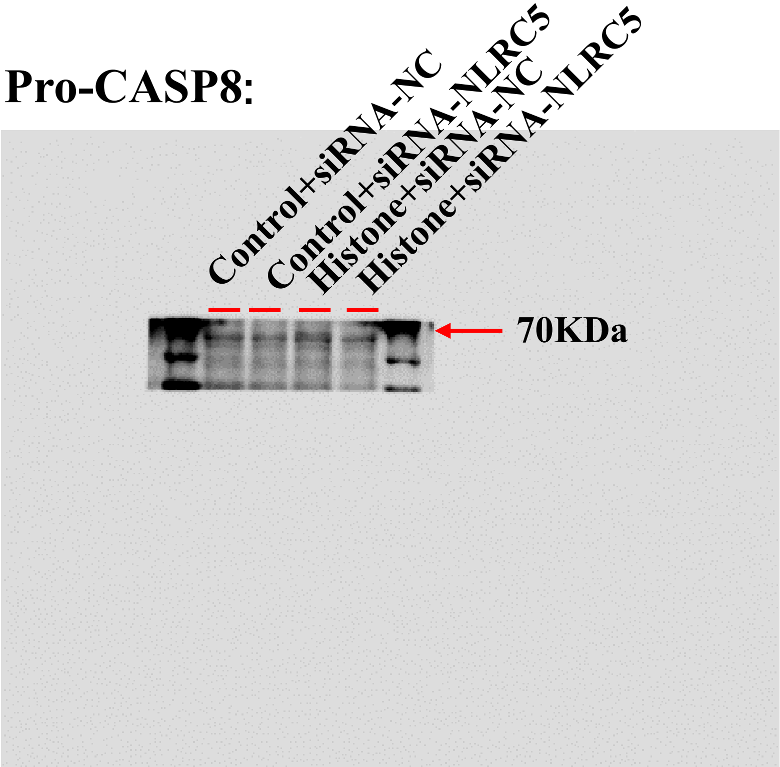

Cleaved-CASP8:

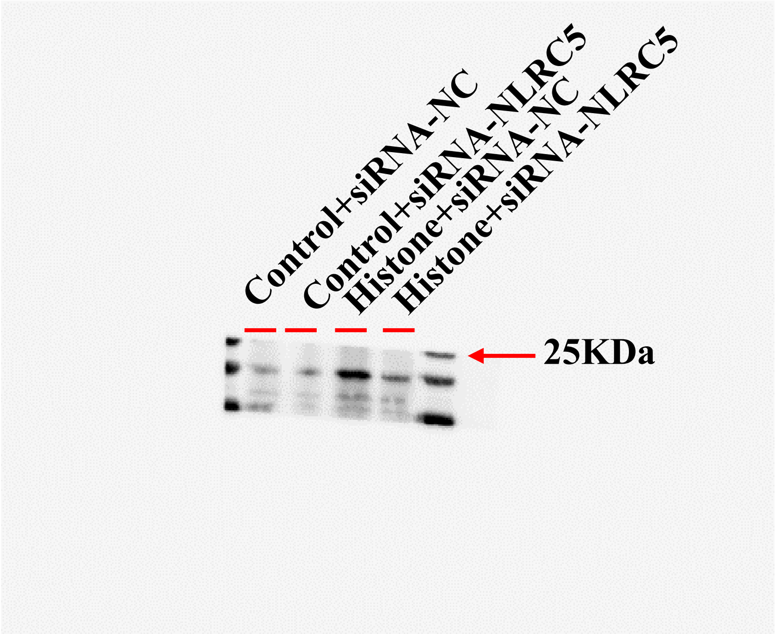

Source data for Fig. 3F

Pro-CASP3:

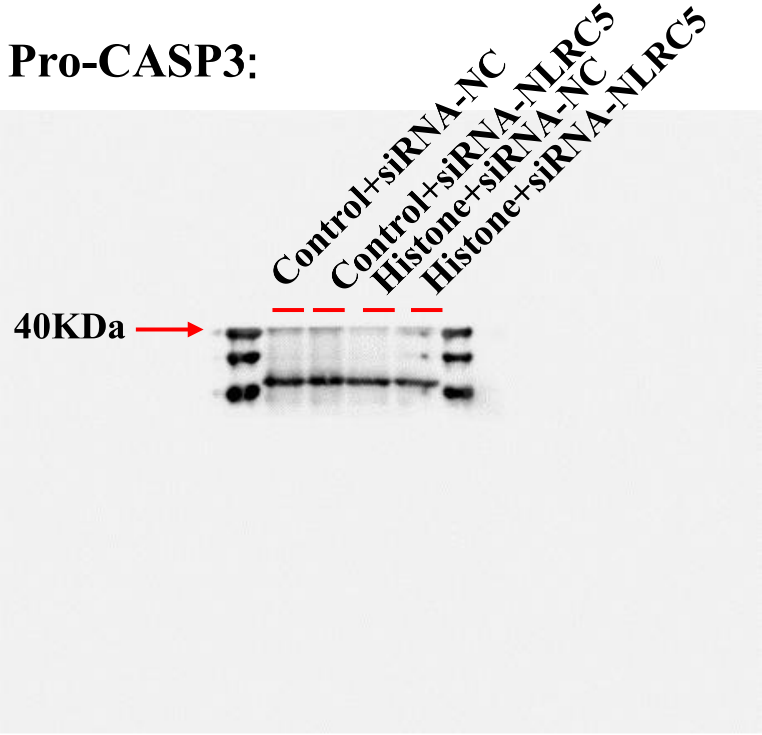

Cleaved-CASP3:

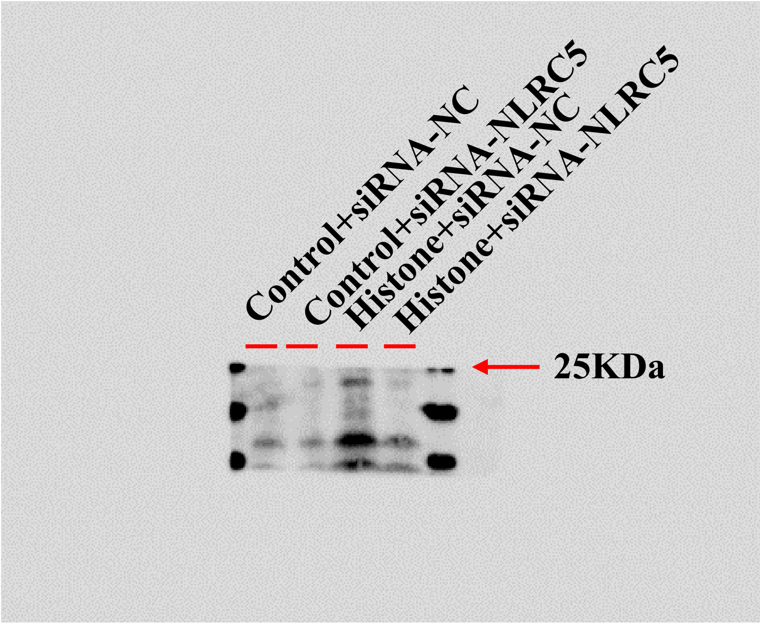

GSDMD:

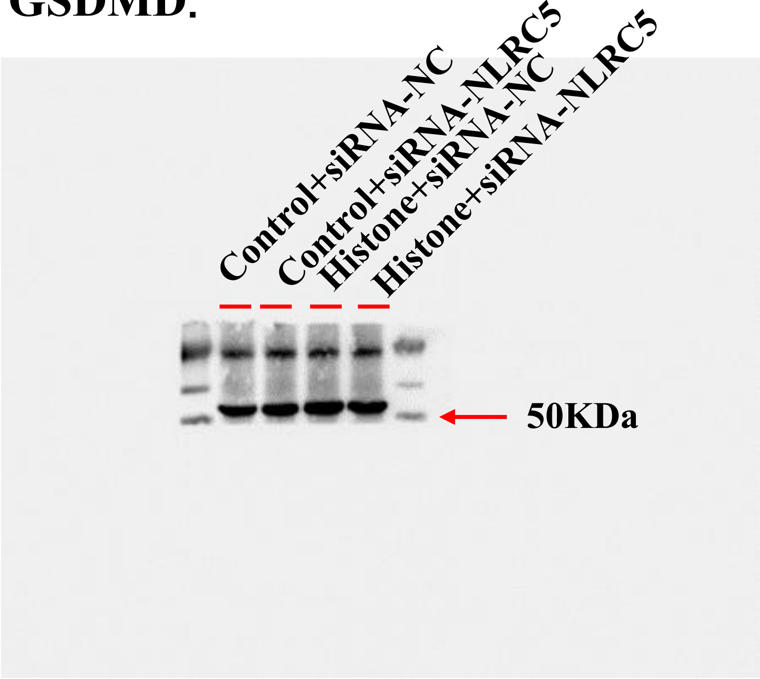

Source data for Fig. 3F

N-GSDMD:

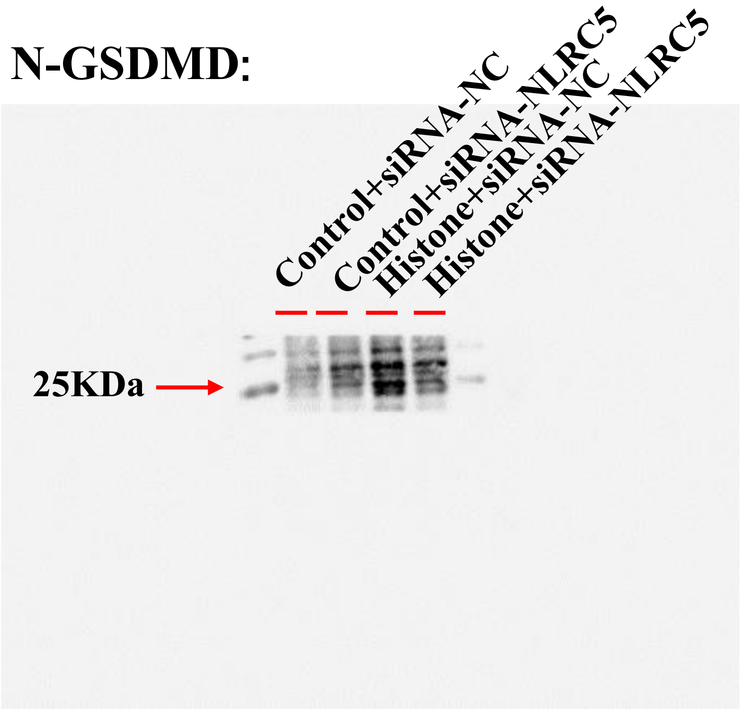

MLKL:

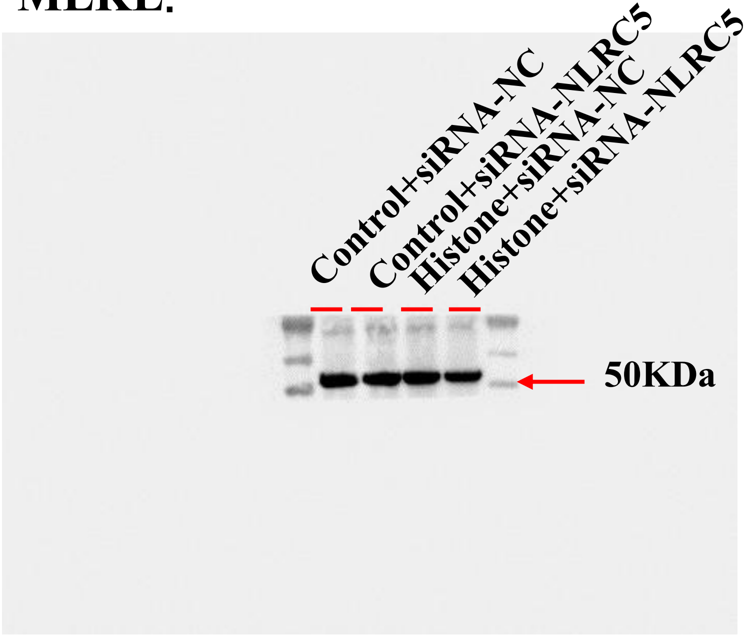

p-MLKL:

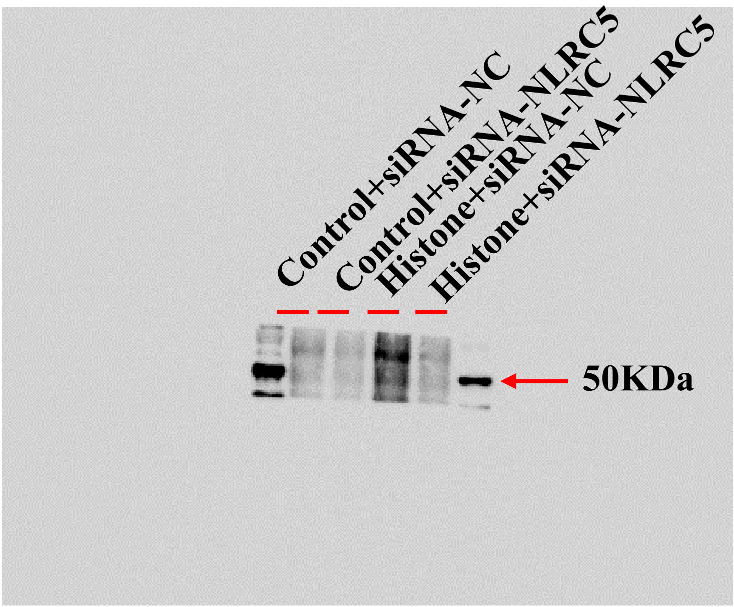

Source data for Fig. 3F

β-actin:

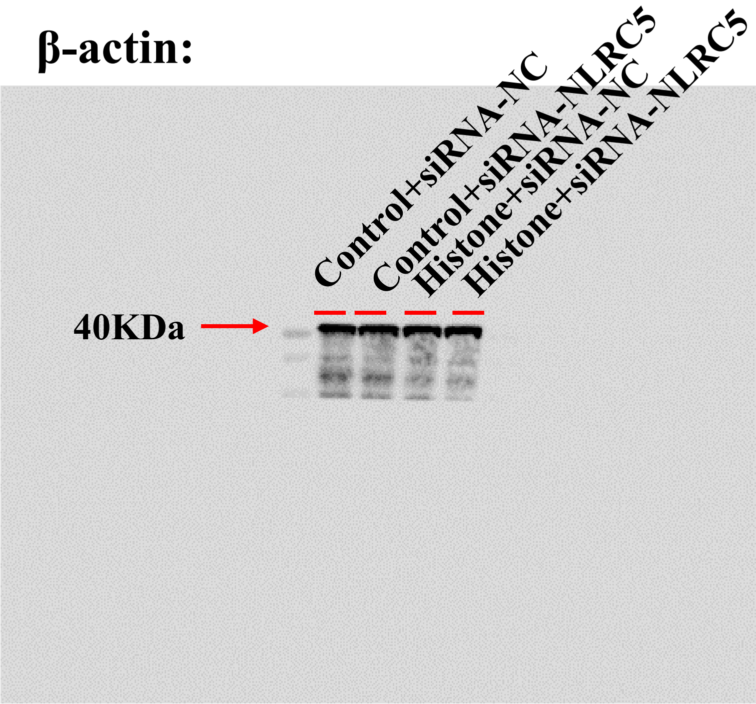

Source data for Fig. 3G

Pro-CASP8:

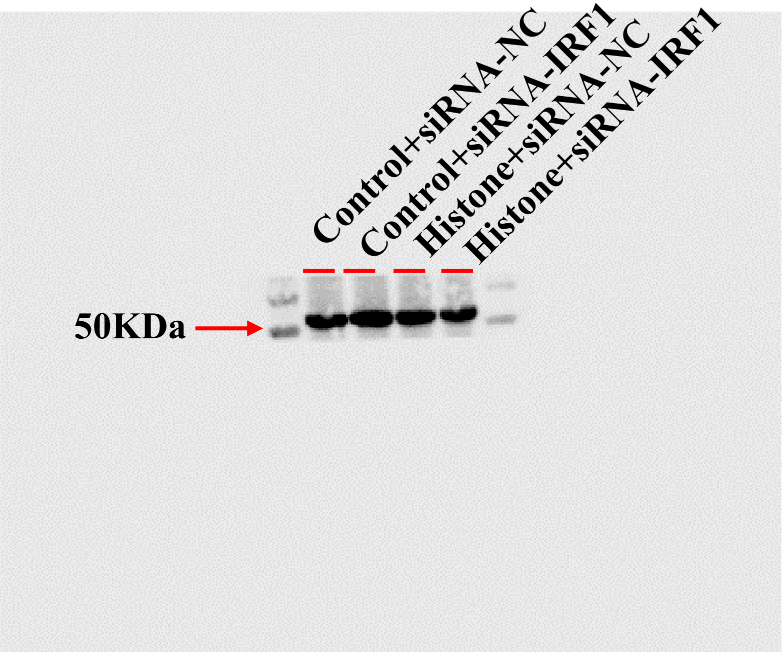

Cleaved-CASP8:

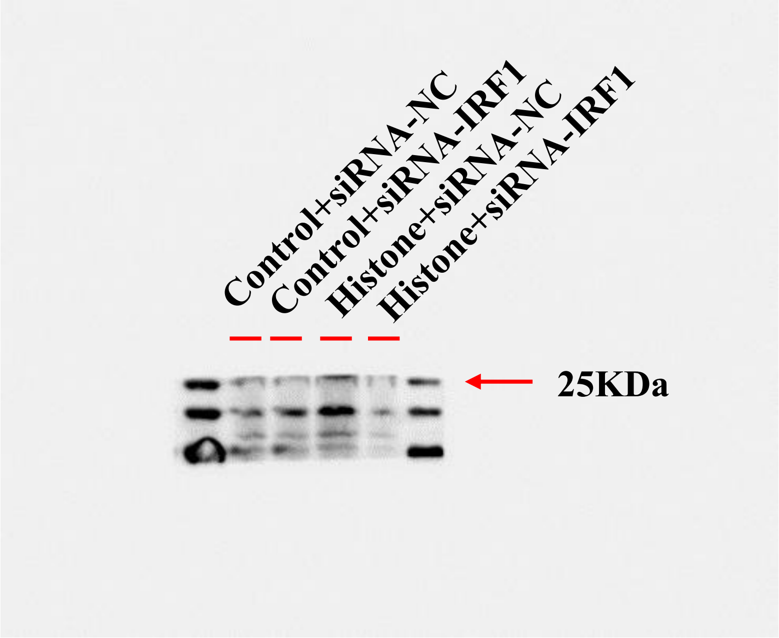

Pro-CASP3:

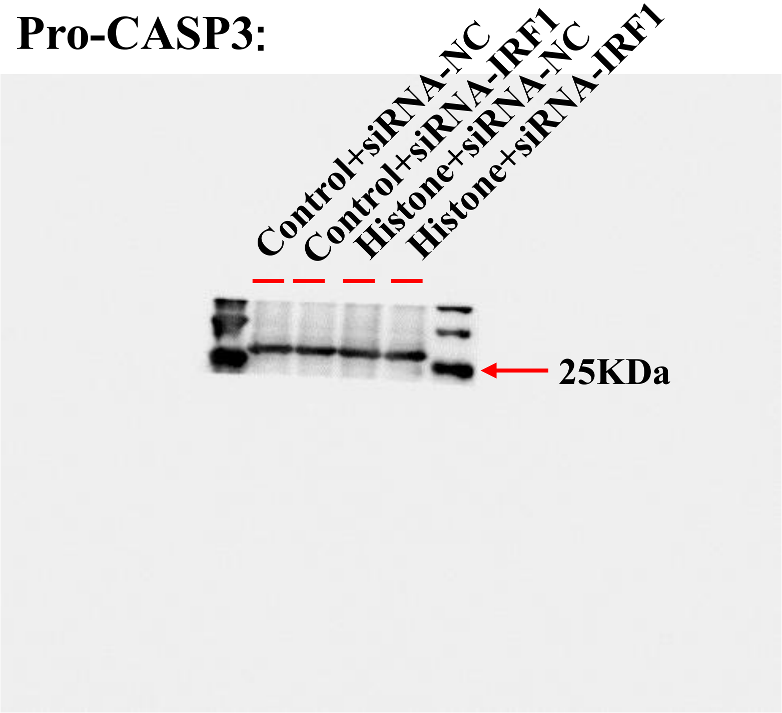

Source data for Fig. 3G

Cleaved-CASP3:

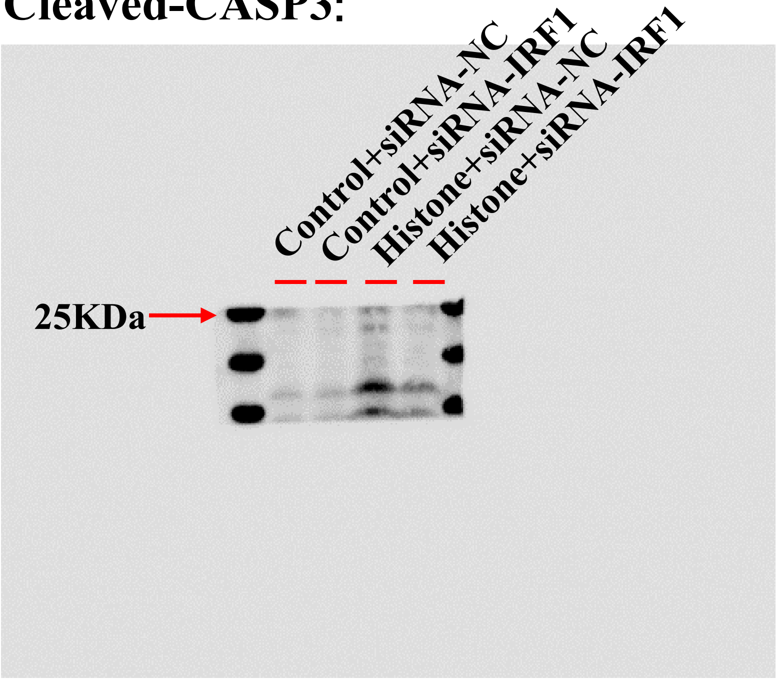

GSDMD:

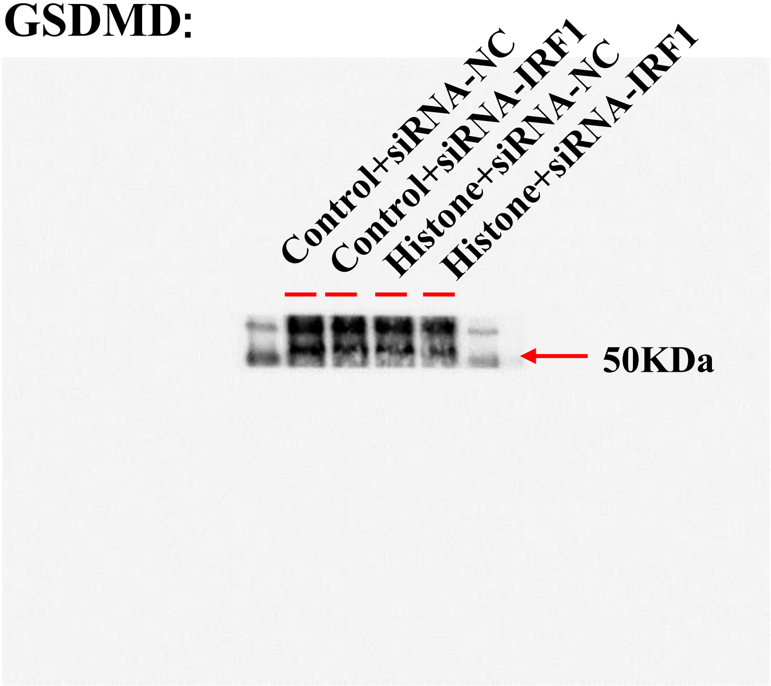

N-GSDMD:

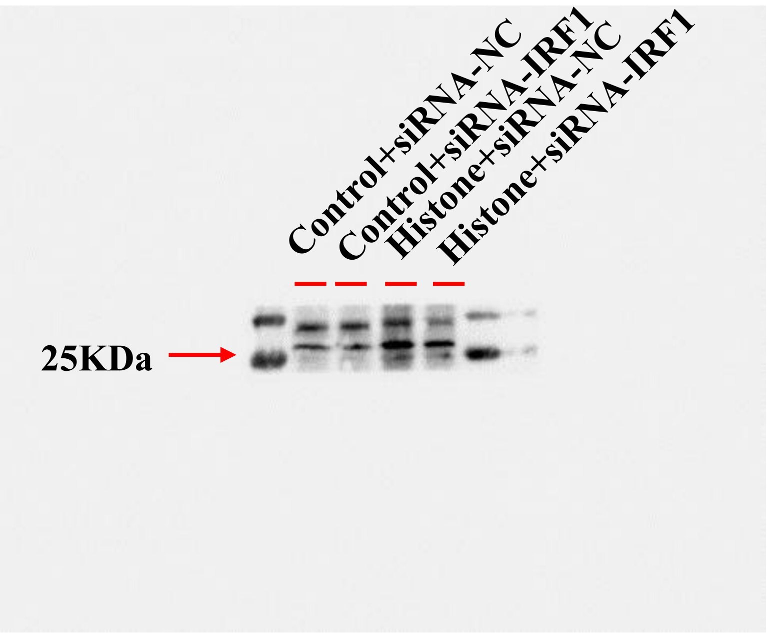

Source data for Fig. 3G

MLKL:

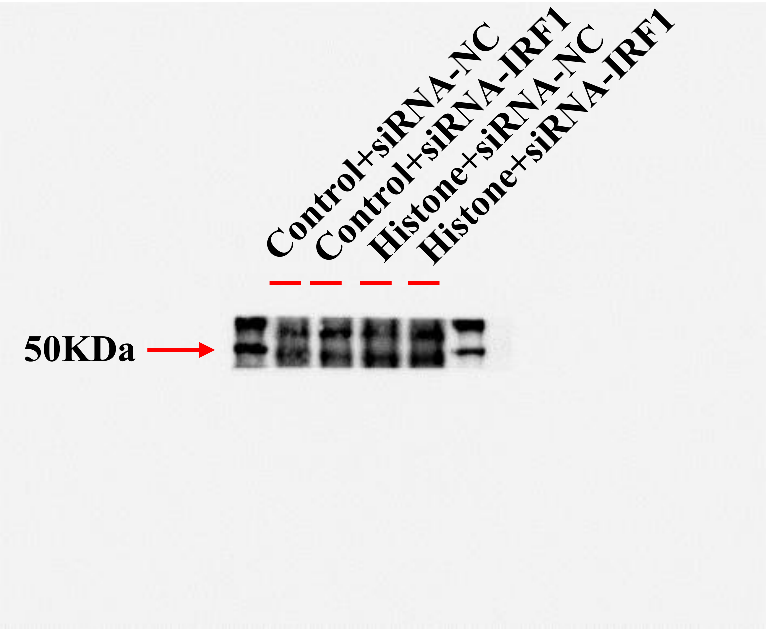

p-MLKL:

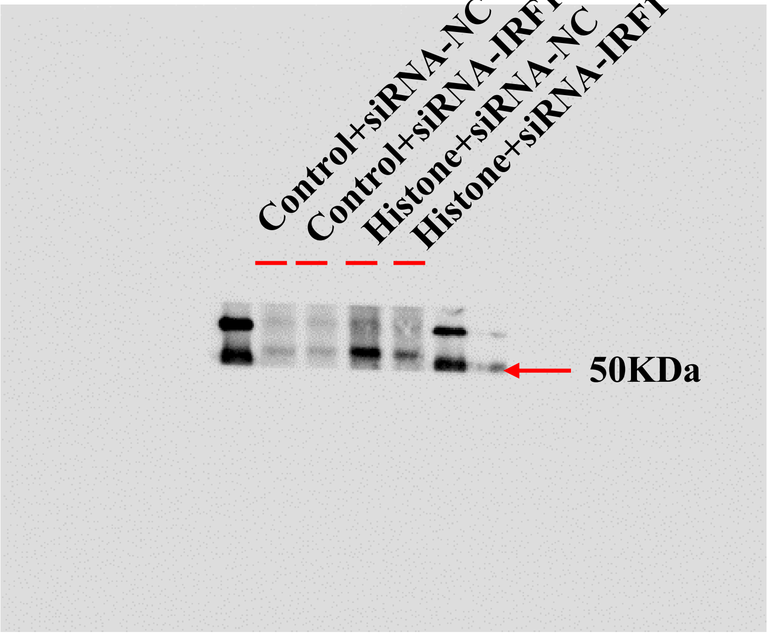

β-actin:

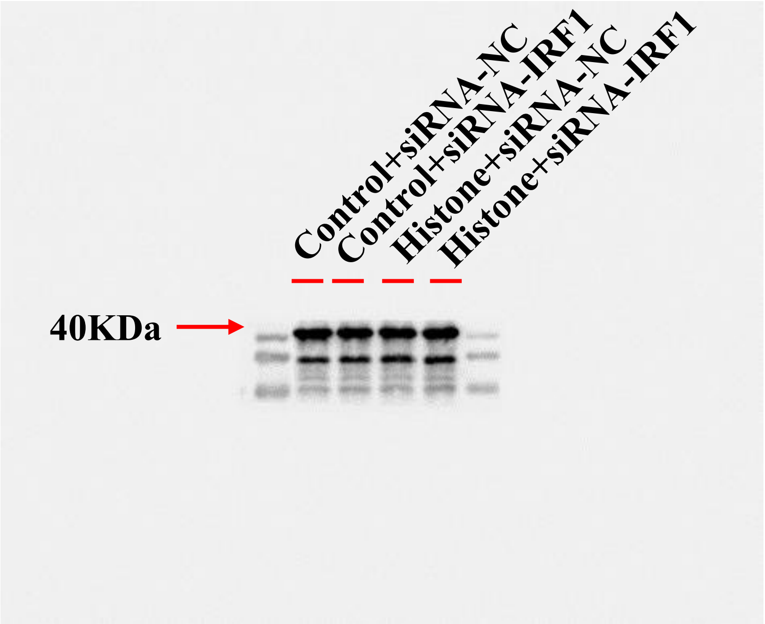

Source data for Fig. 3H

IRF1:

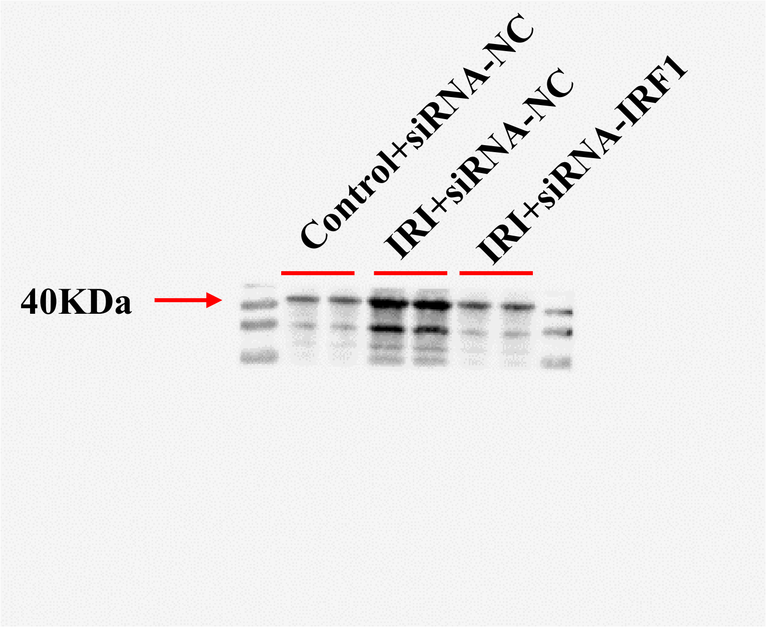

Pro-CASP8:

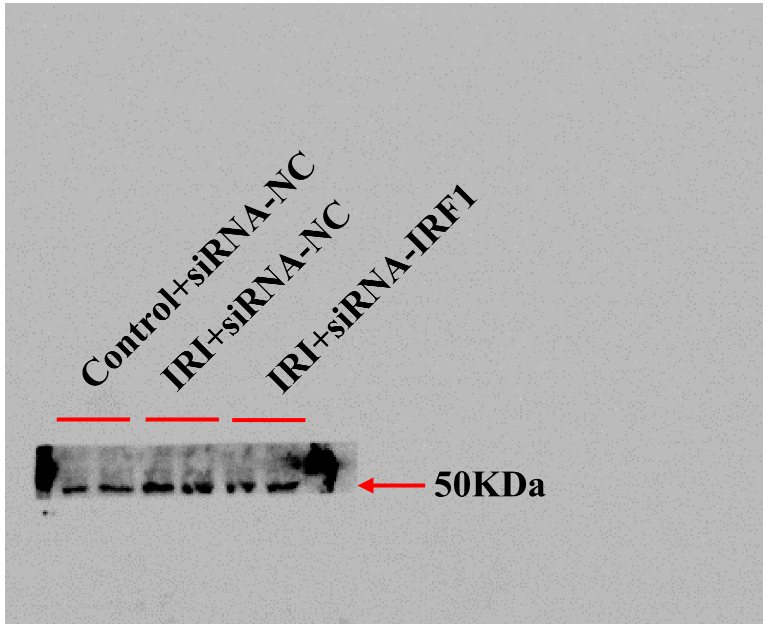

Cleaved-CASP8:

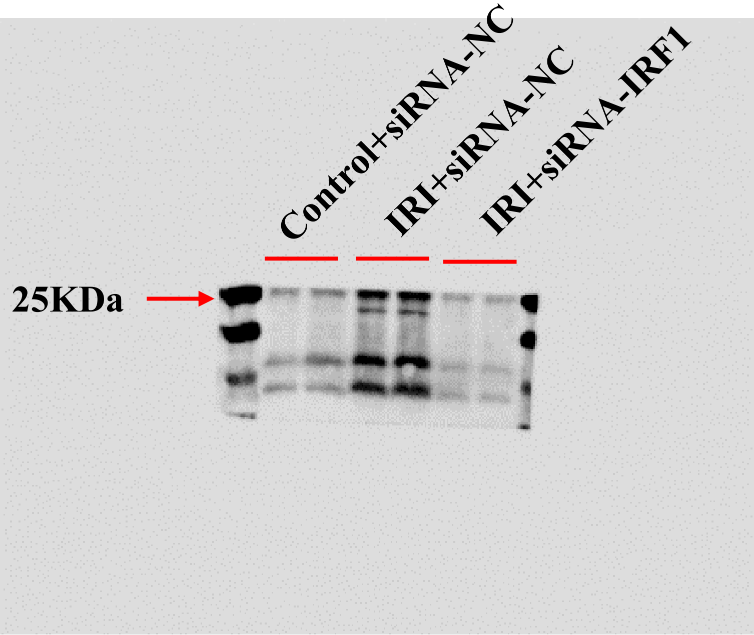

Source data for Fig. 3H

Pro-CASP3:

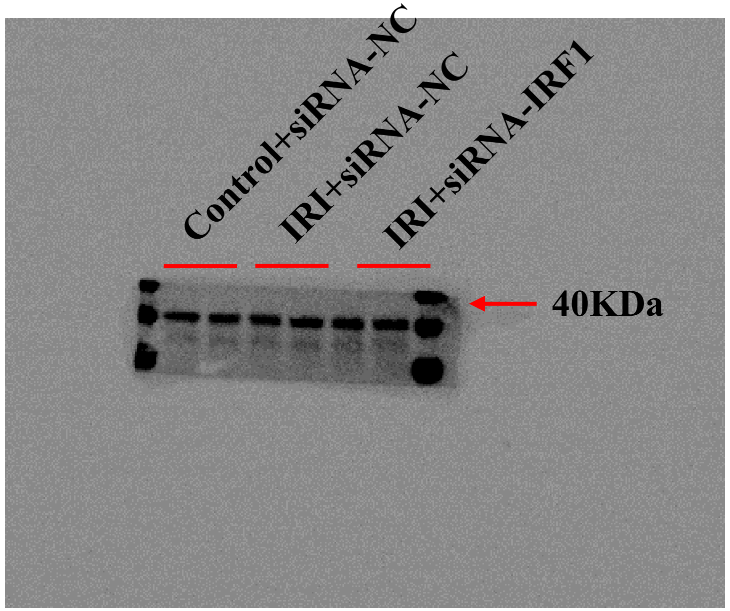

Cleaved-CASP3:

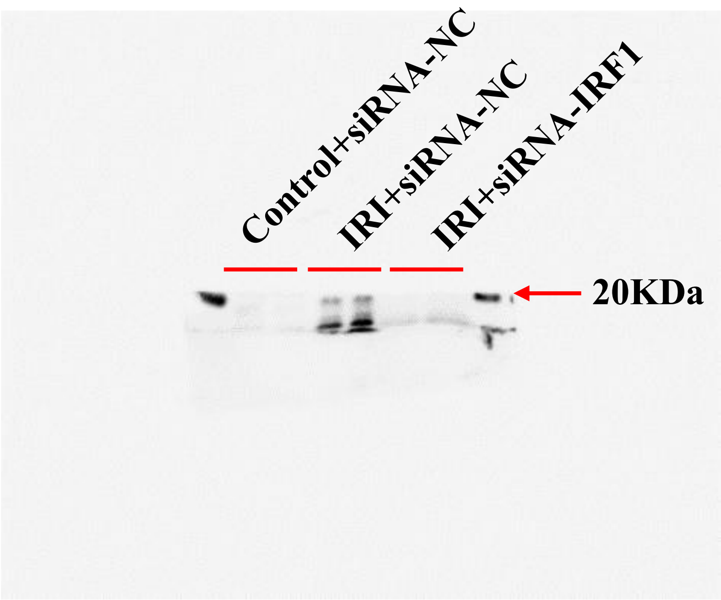

GSDMD:

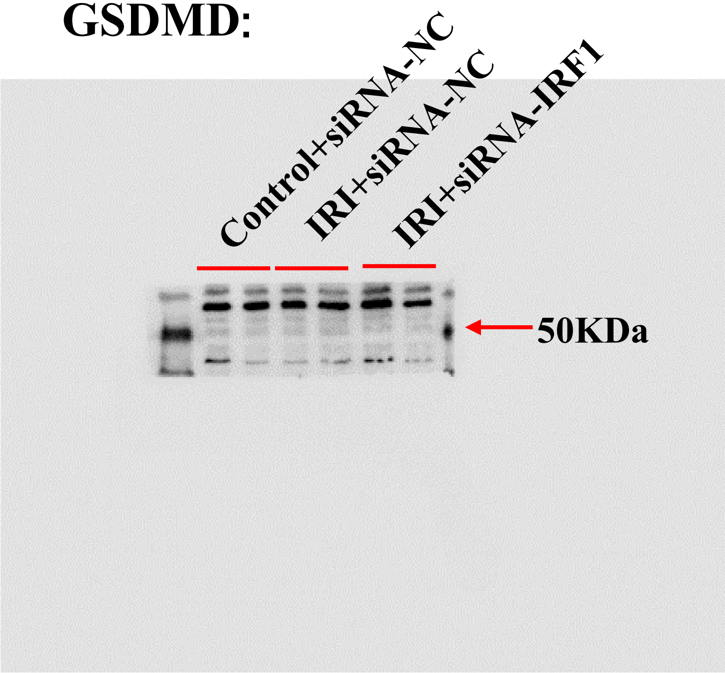

Source data for Fig. 3H

N-GSDMD:

MLKL:

p-MLKL:

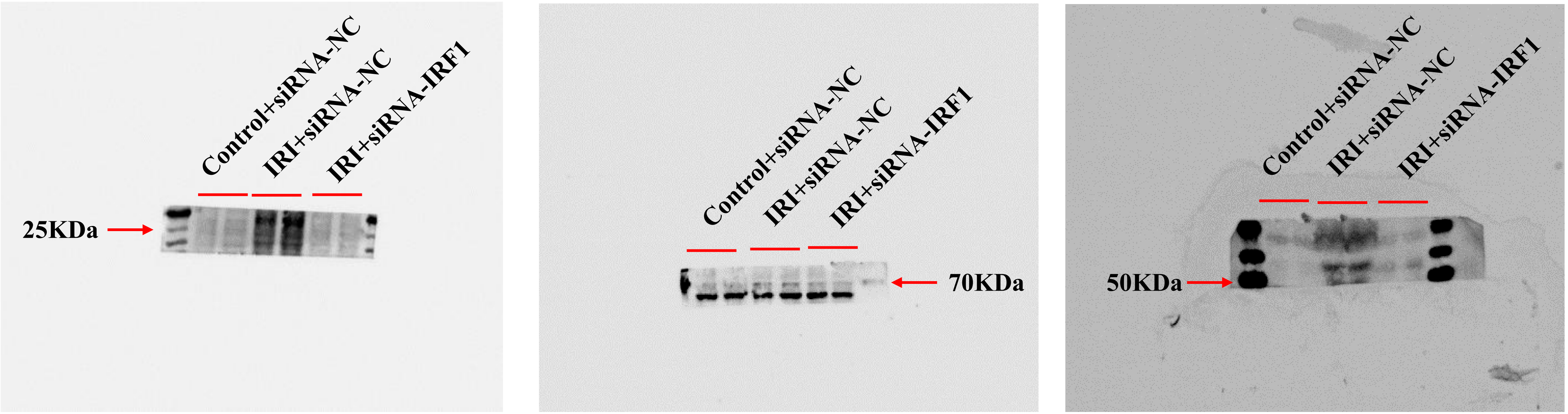

Source data for Fig. 3H

β-actin:

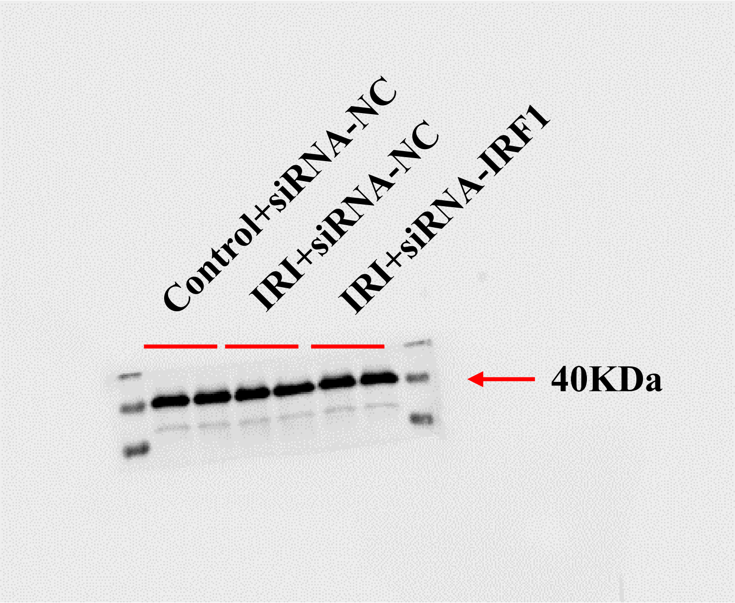

Source data for Fig. 5Q

IRF1:

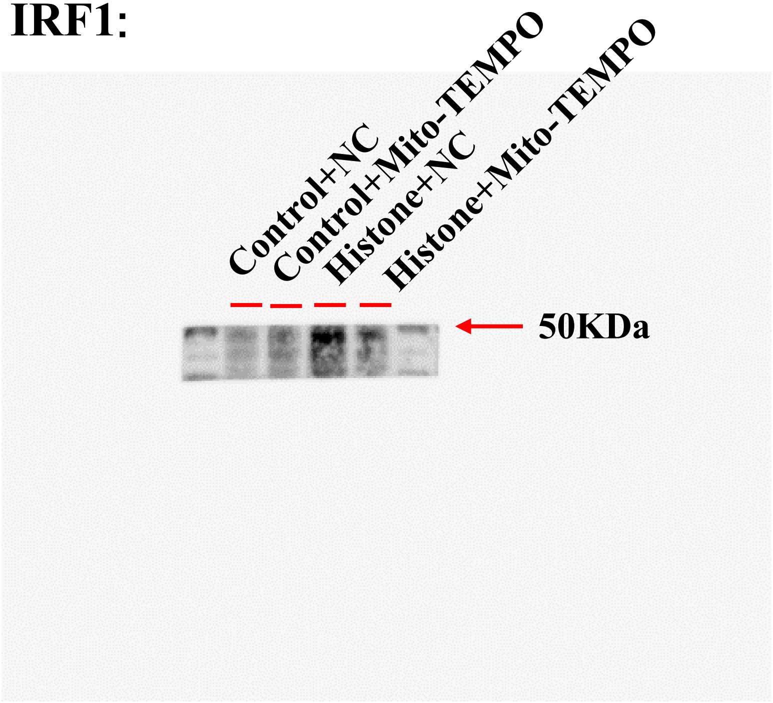

NLRC5:

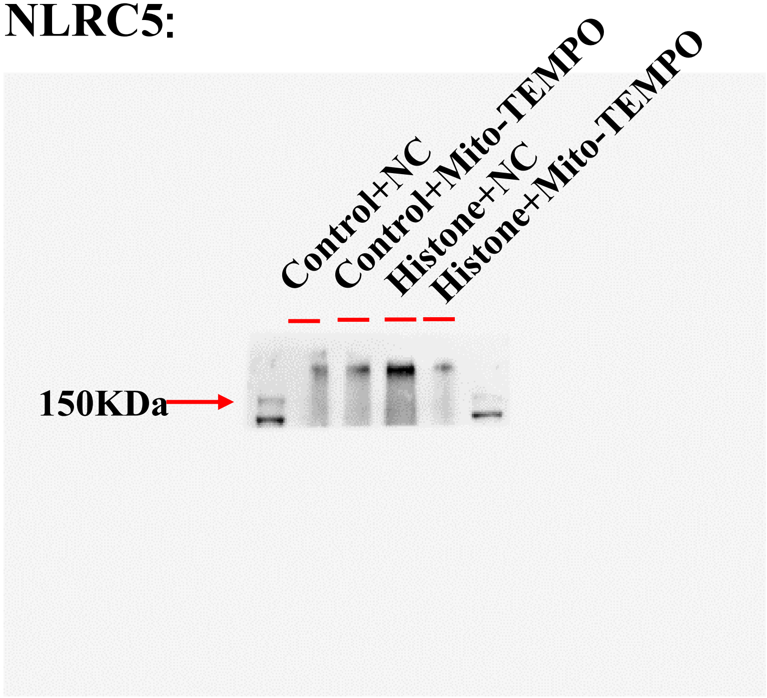

β-actin:

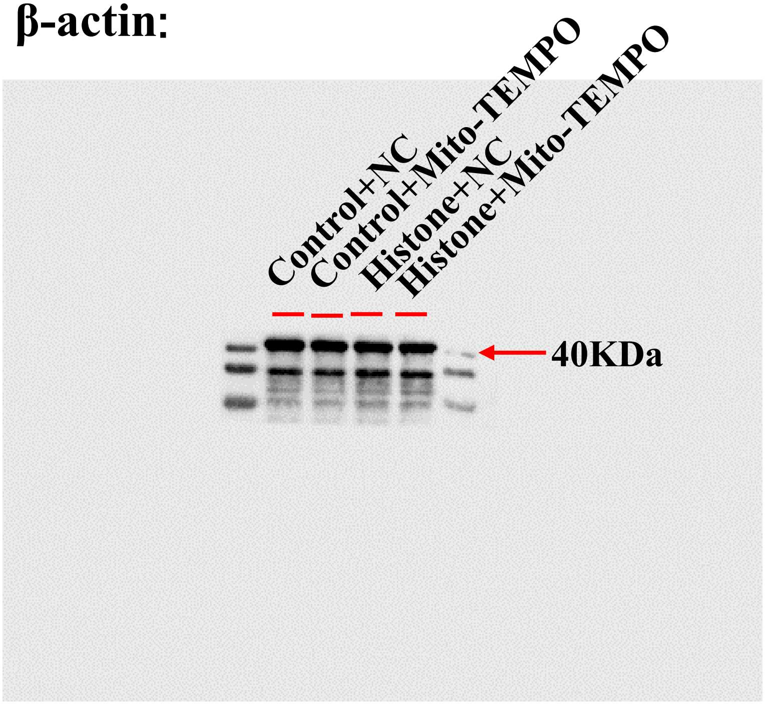

Source data for Fig. 6A

Drp1:

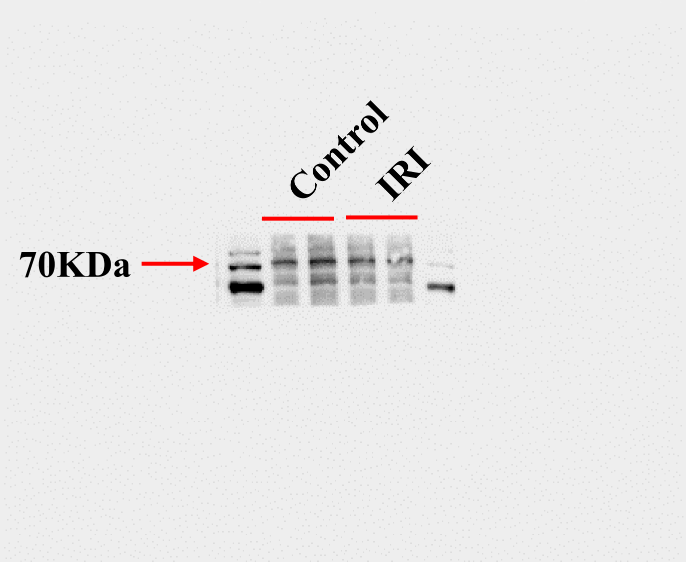

p-Drp1(Ser616):

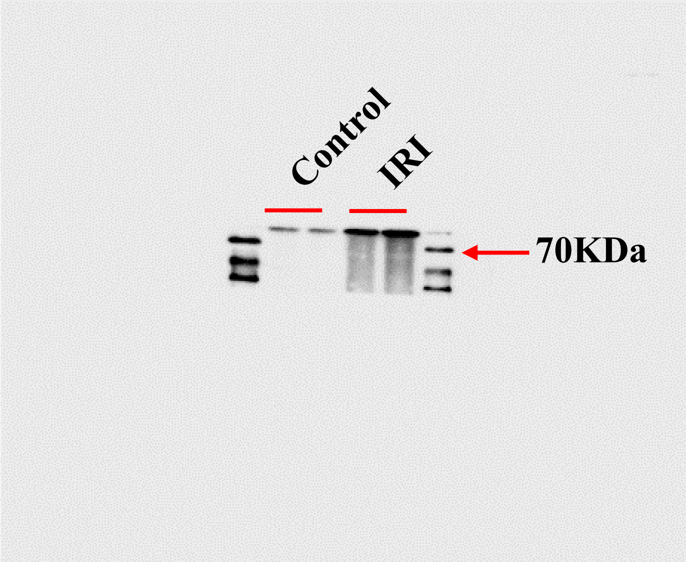

Fis1:

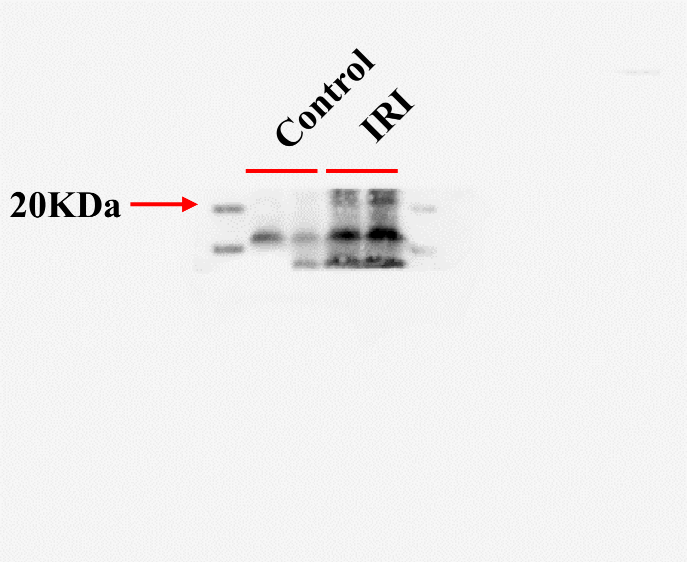

β-actin:

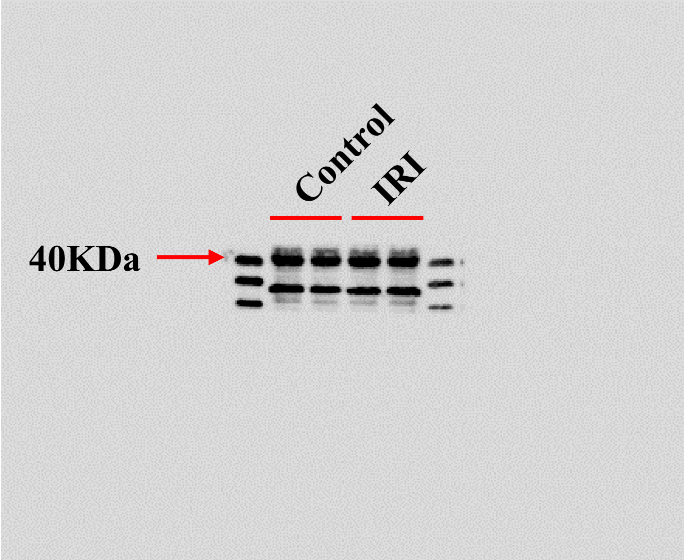

Source data for Fig. 6D

IRF1:

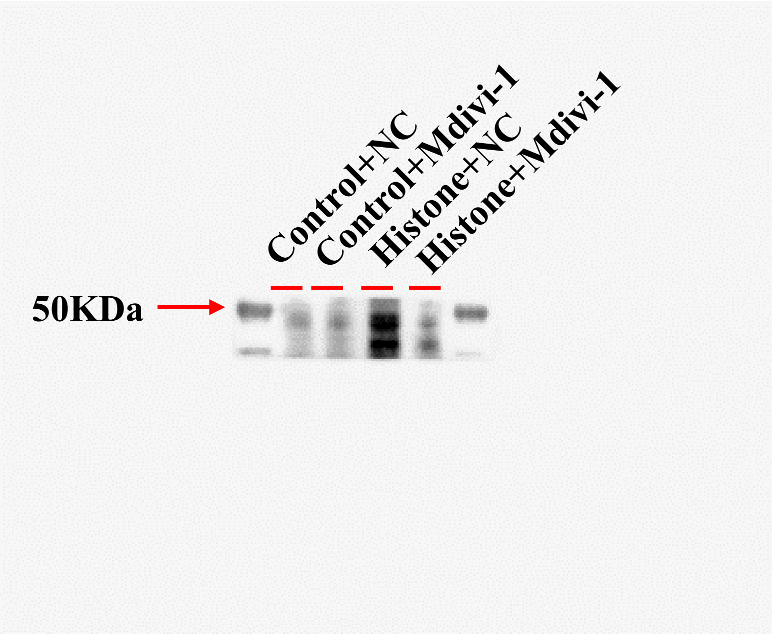

NLRC5:

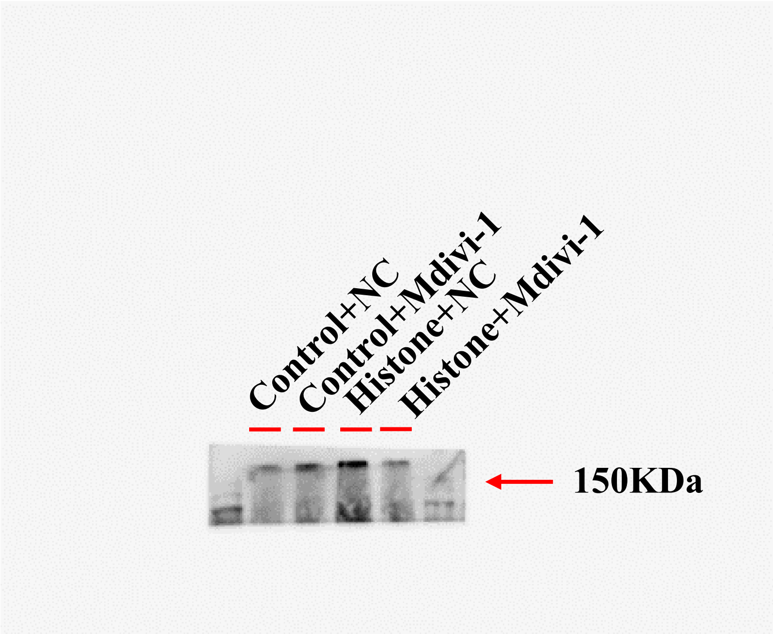

β-actin:

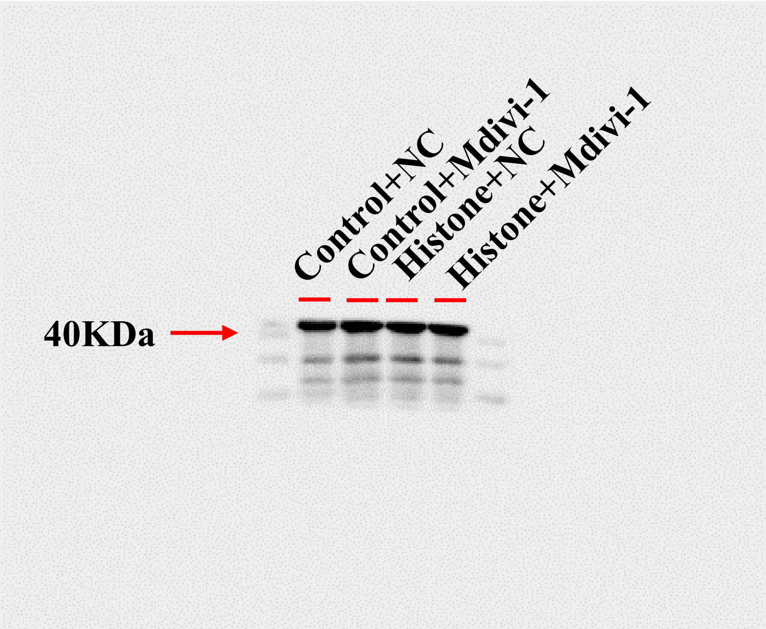

Source data for Fig. 6K

Pro-CASP8:

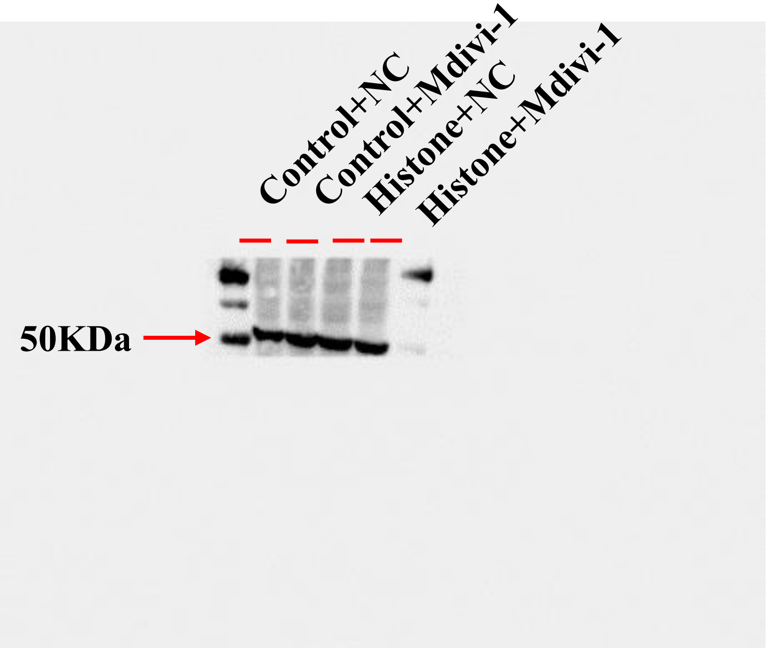

Cleaved-CASP8:

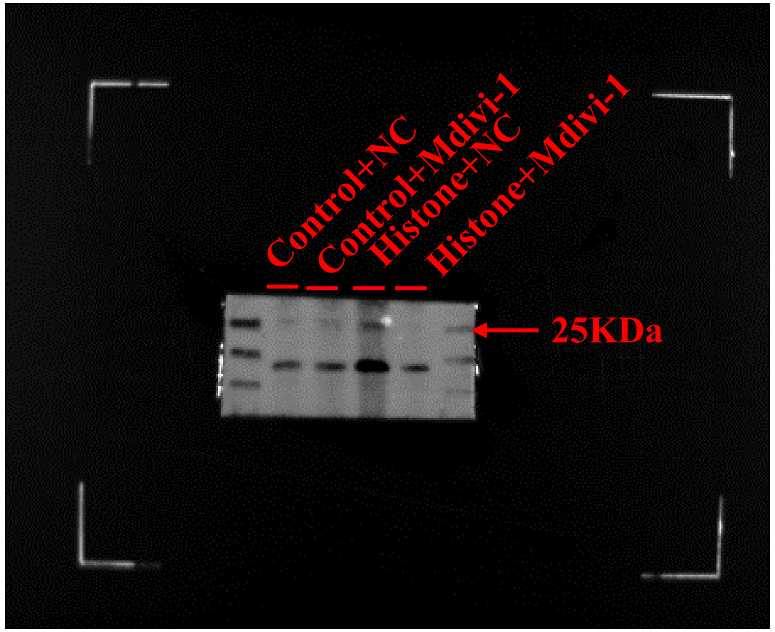

Pro-CASP3:

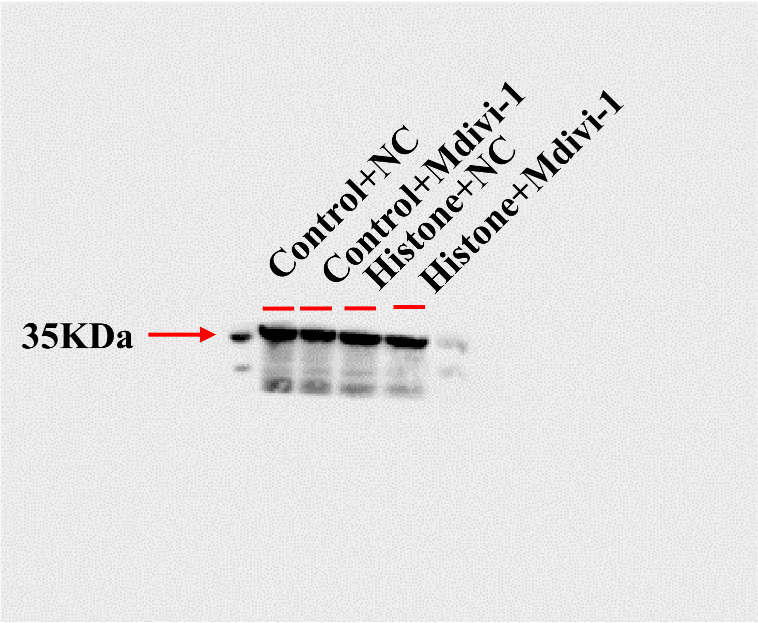

Source data for Fig. 6K

Cleaved-CASP3:

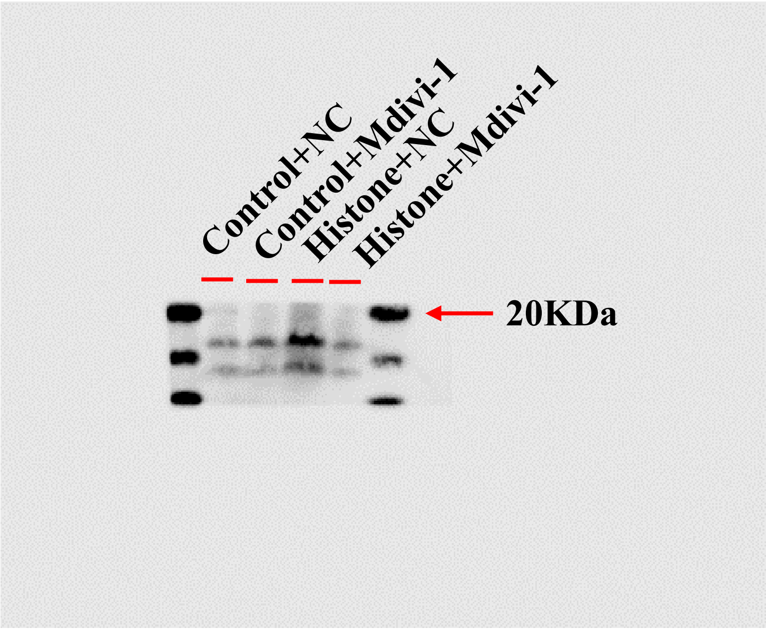

GSDMD:

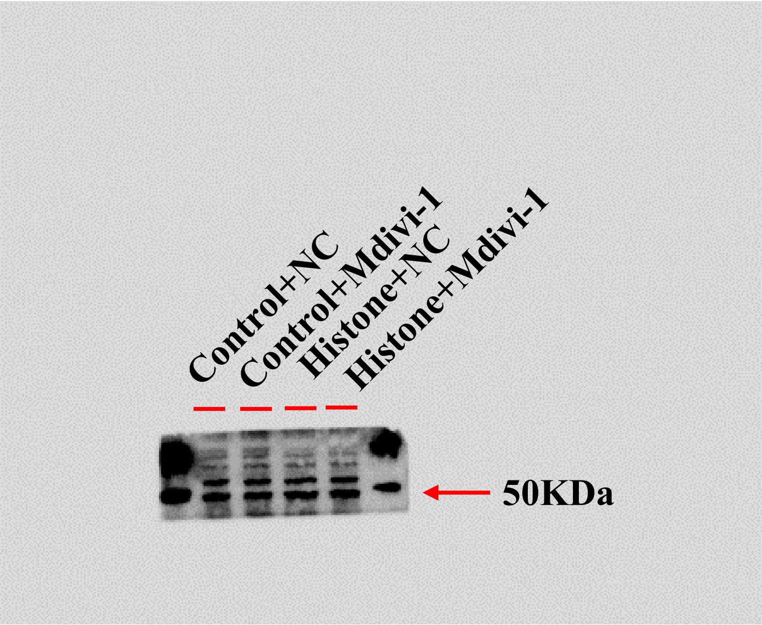

N-GSDMD:

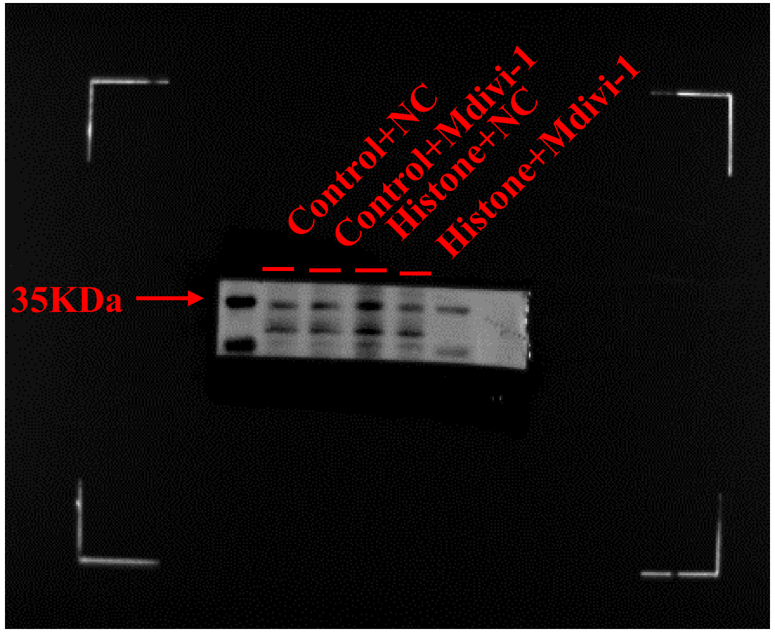

Source data for Fig. 6K

MLKL:

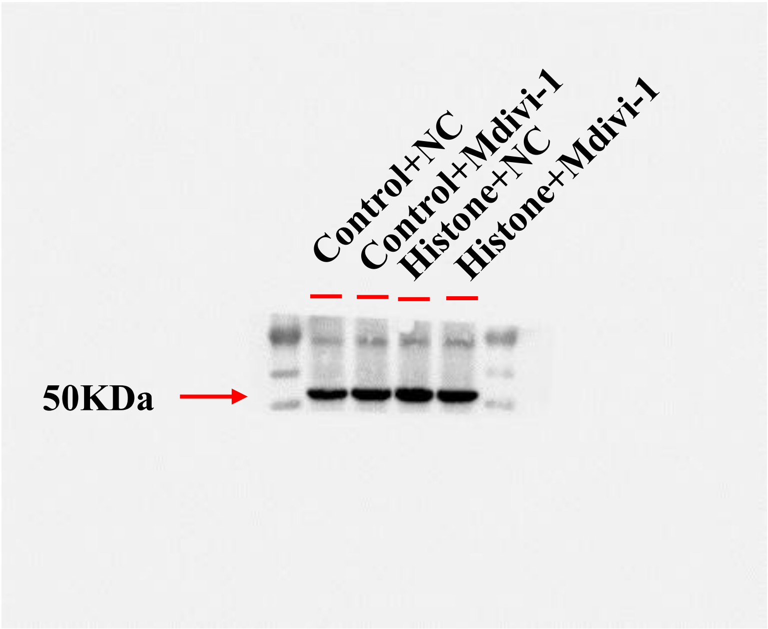

p-MLKL:

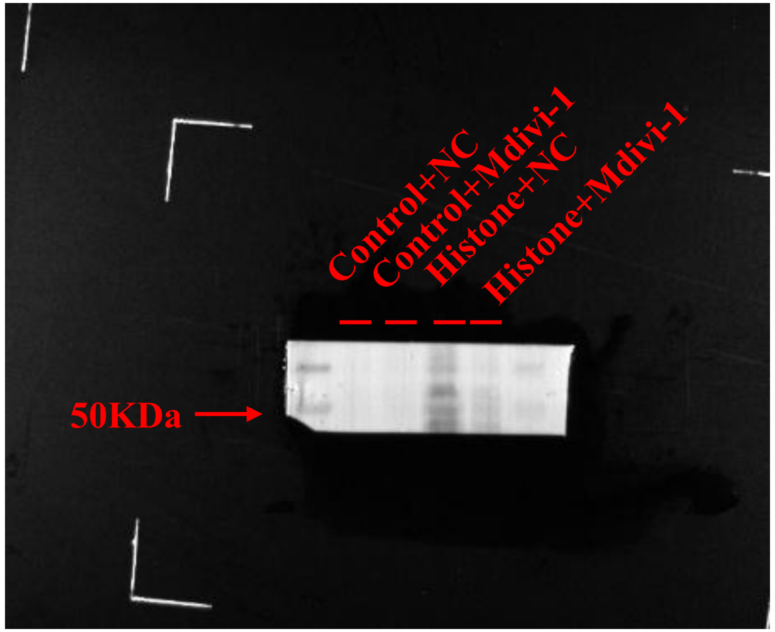

β-actin:

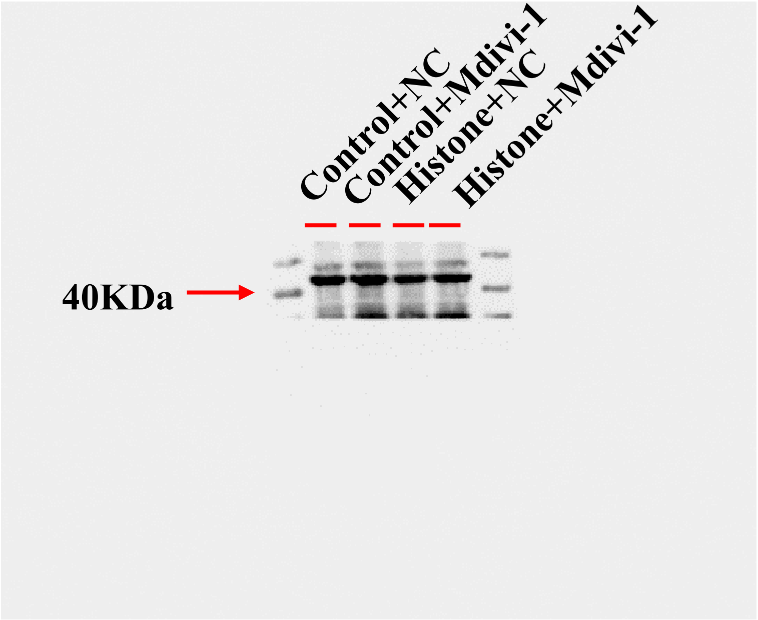

Source data for Fig. 8C

Drp1:

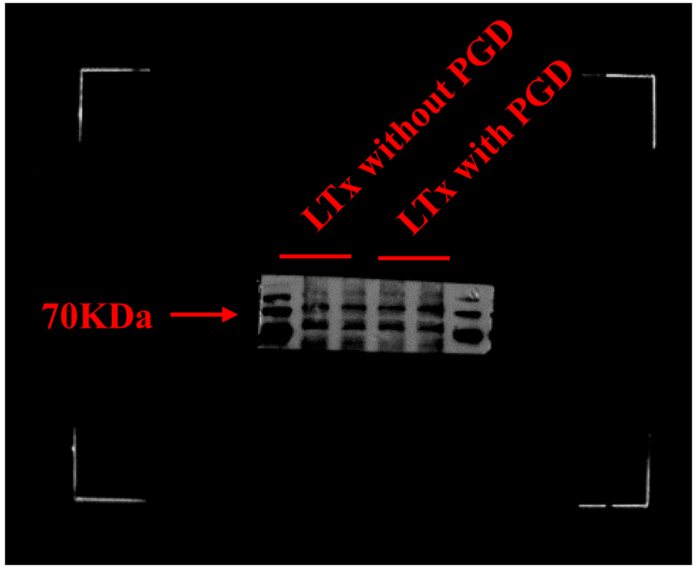

p-Drp1(Ser616):

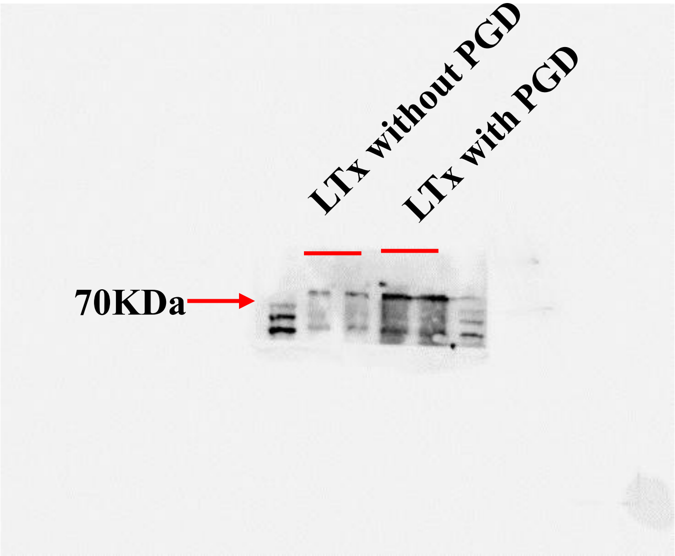

NLRC5:

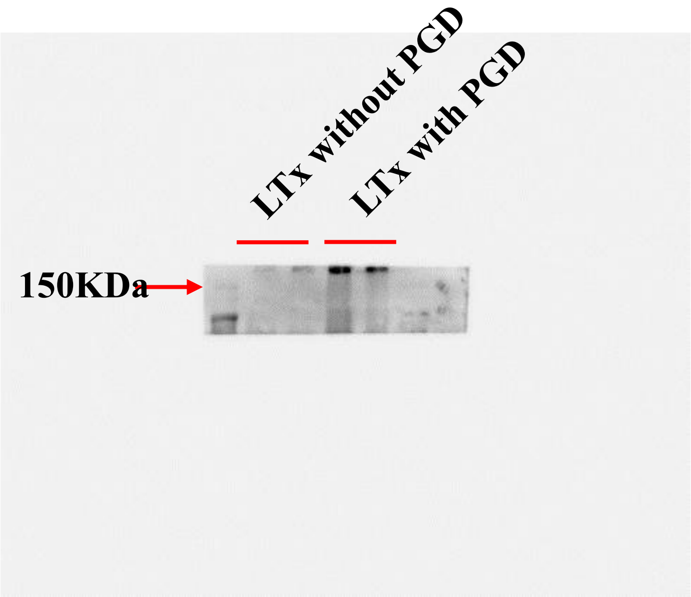

β-actin:

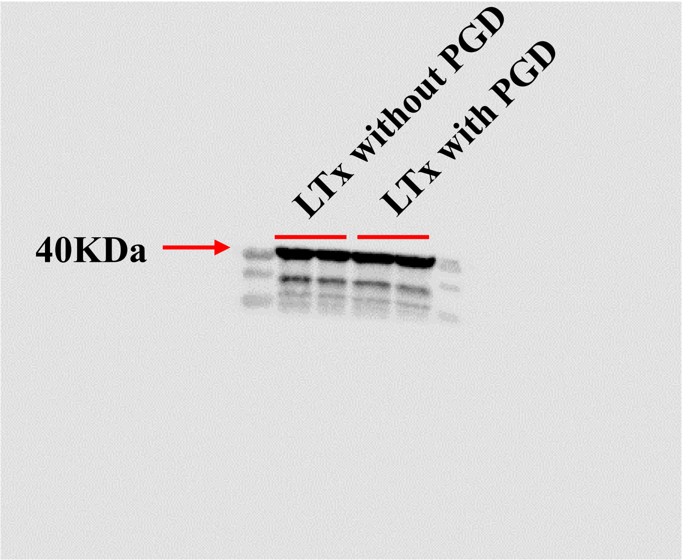

Supplement: Supplementary file 1 — Supplementary Material 1. [file 10495_2026_2401_MOESM1_ESM.pdf]
