# Supplementary figures and images for: IRF1-mediated sensing of oxidized mitochondrial DNA drives macrophage PANoptosis in lung ischemia–reperfusion injury
Source: Apoptosis. 2026 Jul 25;31(8):198. doi: 10.1007/s10495-026-02401-3 (PMC13401555; doi:10.1007/s10495-026-02401-3)

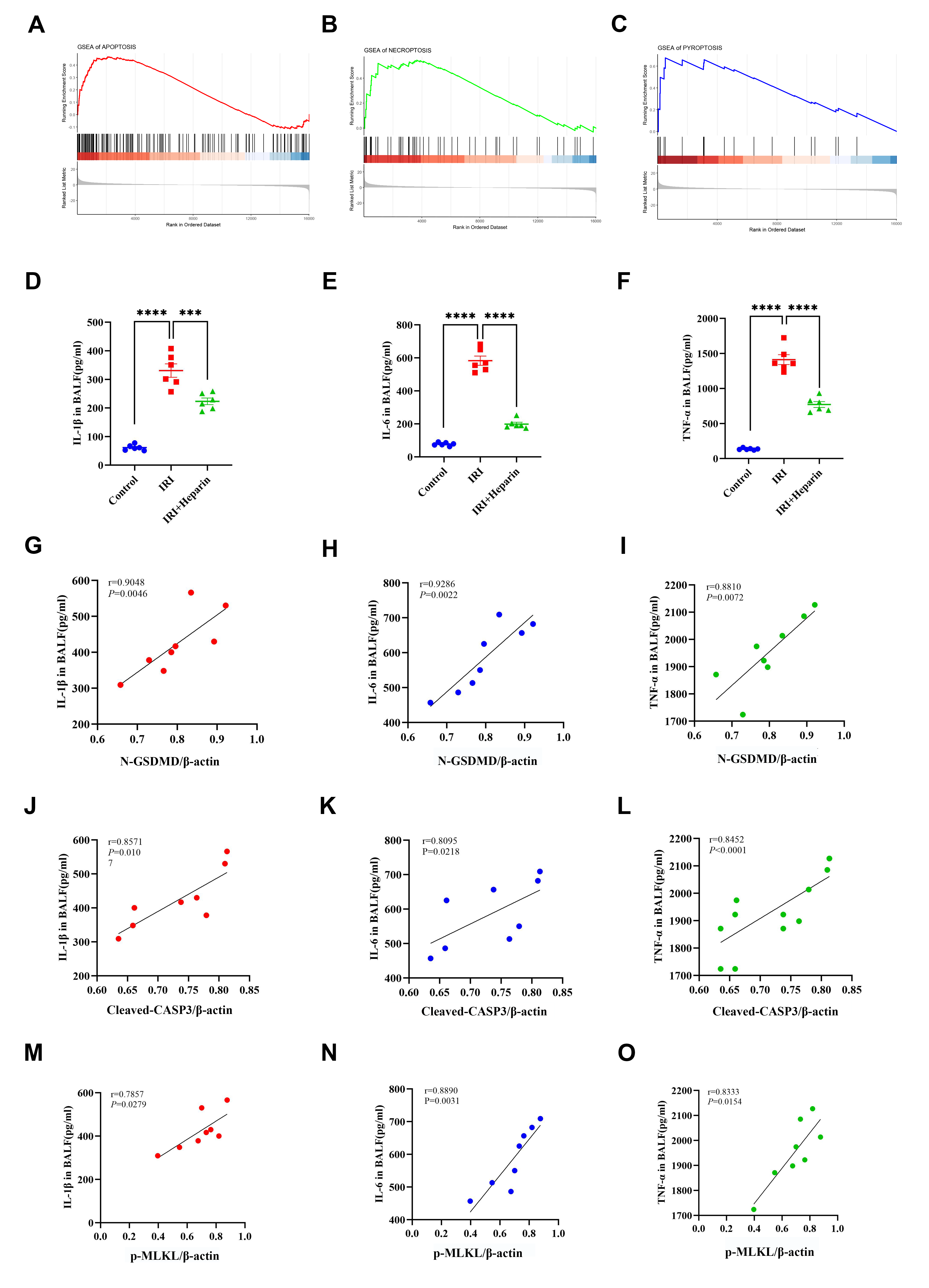

Supplement: Supplementary file 2 — Supplementary Material 2. [file 10495_2026_2401_MOESM2_ESM.tif]

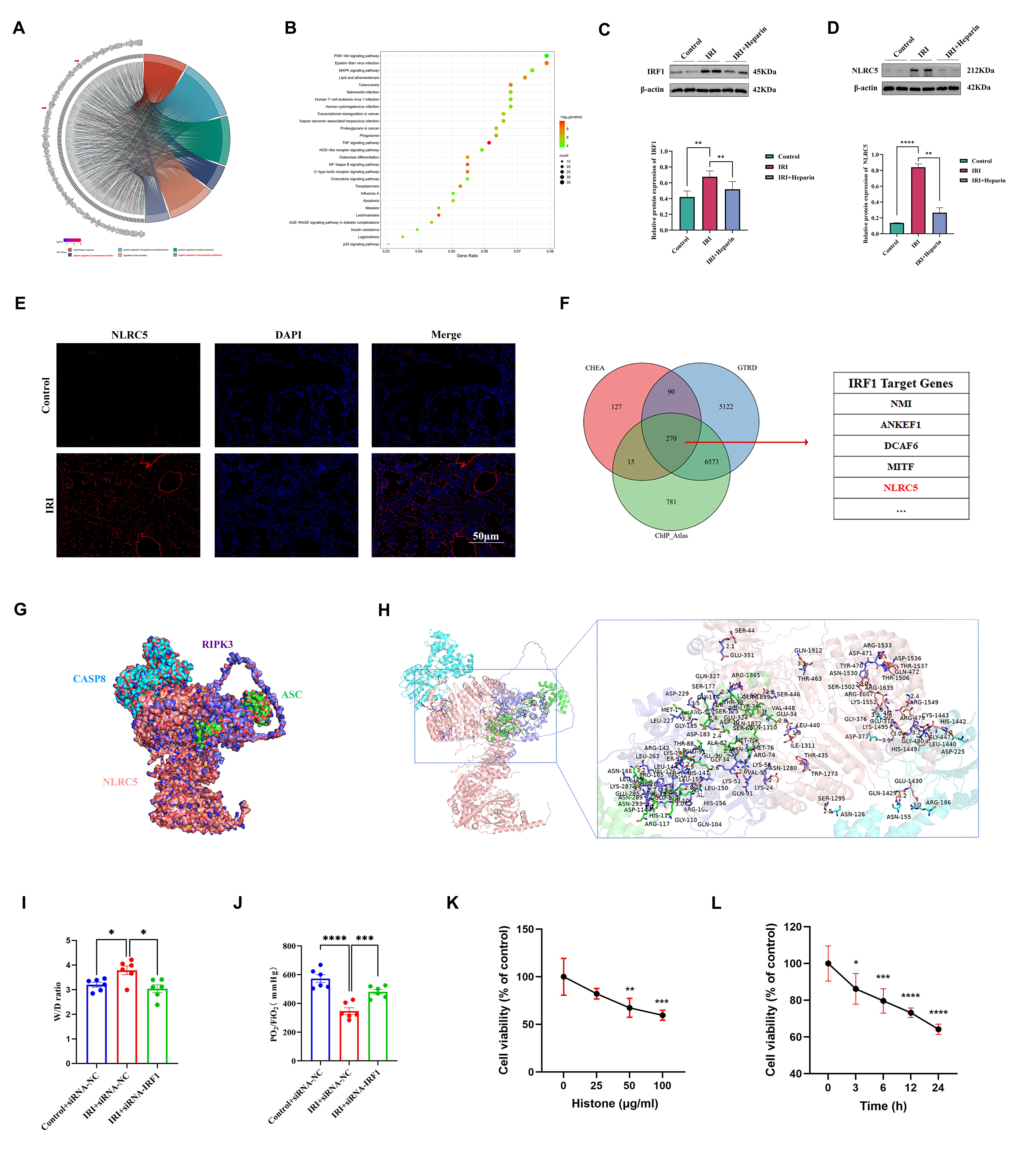

Supplement: Supplementary file 3 — Supplementary Material 3. [file 10495_2026_2401_MOESM3_ESM.tif]

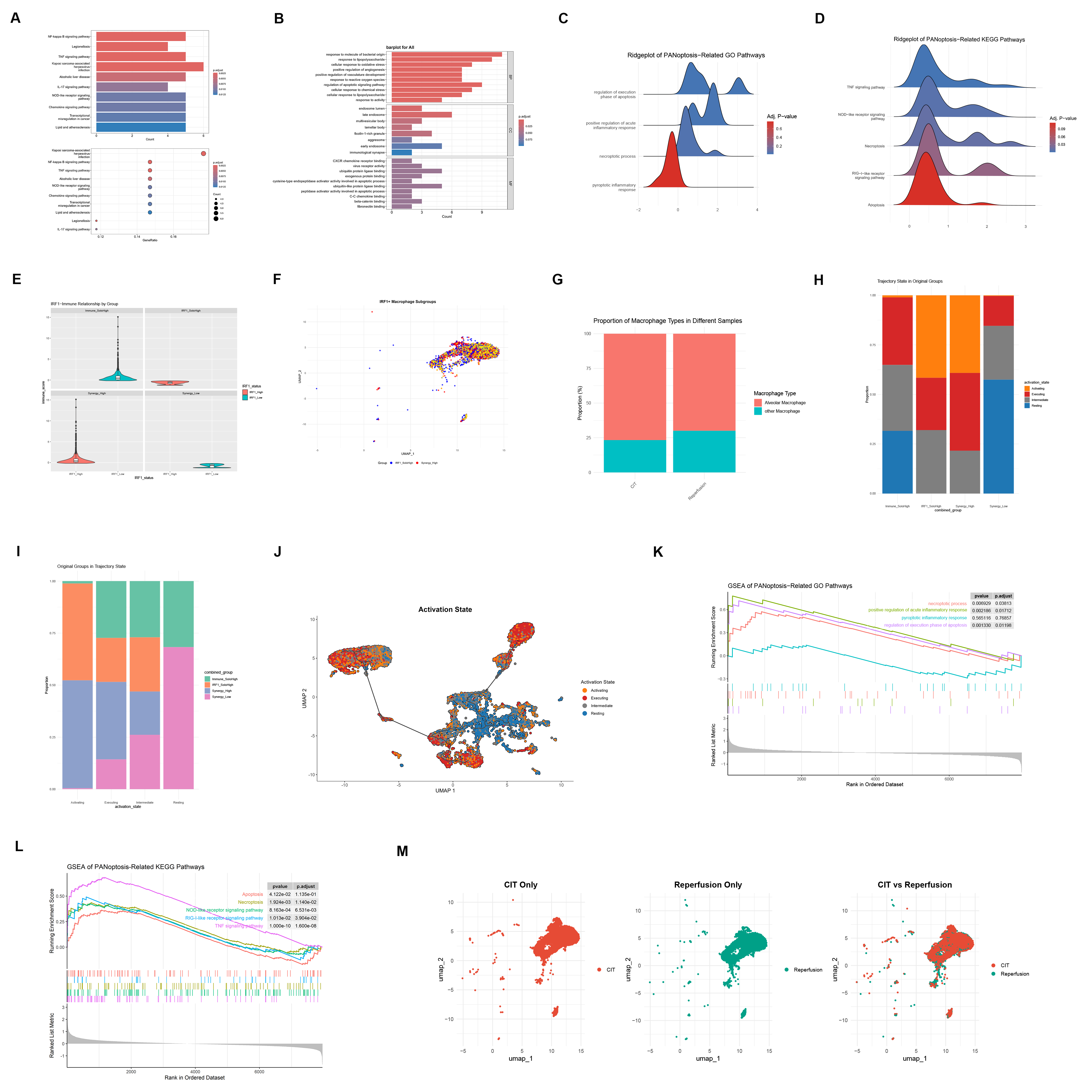

Supplement: Supplementary file 4 — Supplementary Material 4. [file 10495_2026_2401_MOESM4_ESM.tif]

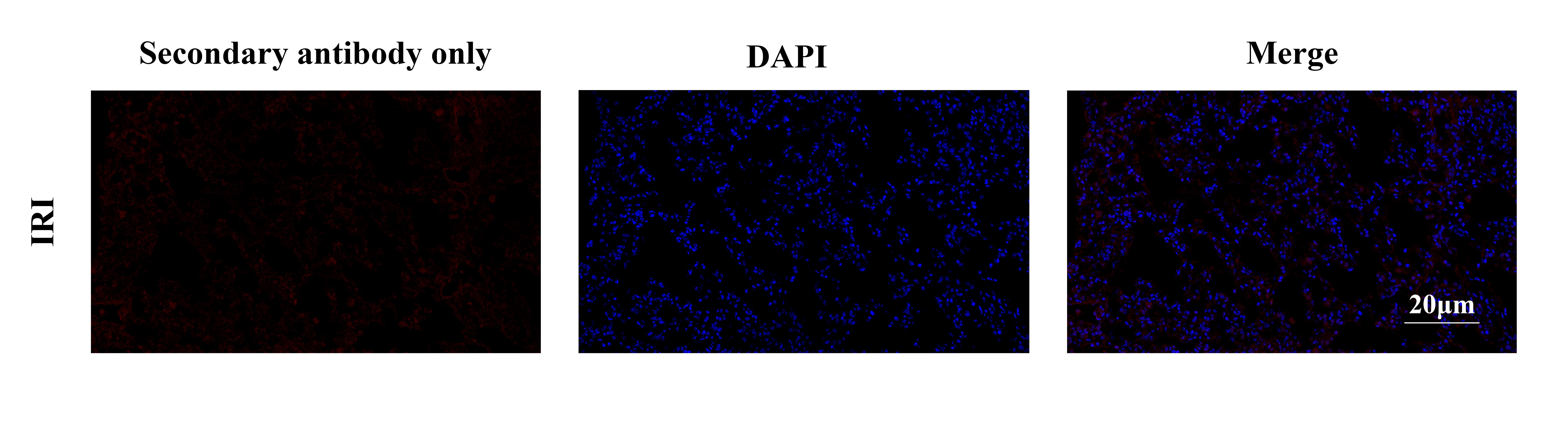

Supplement: Supplementary file 5 — Supplementary Material 5. [file 10495_2026_2401_MOESM5_ESM.tif]

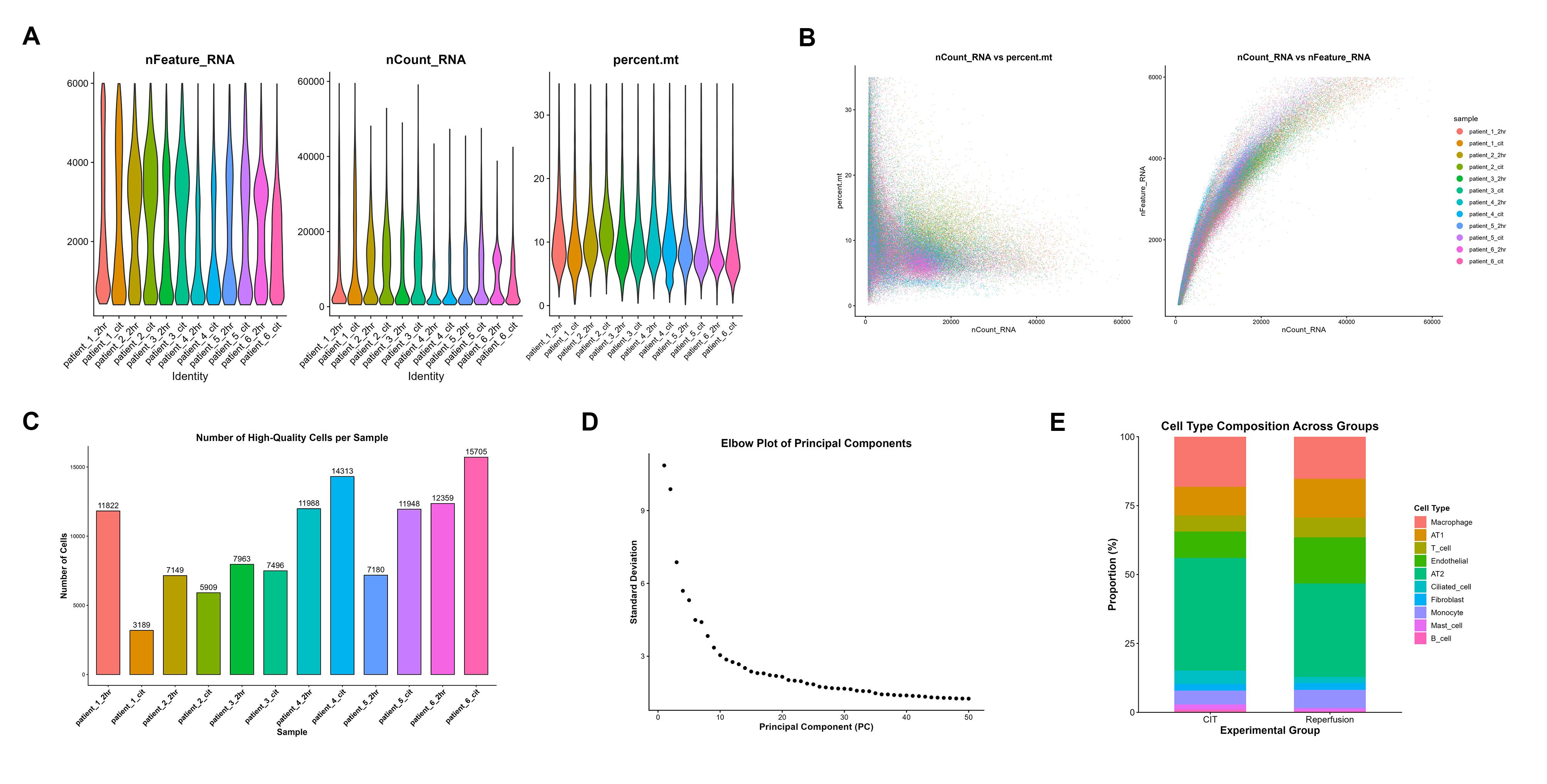

Supplement: Supplementary file 6 — Supplementary Material 6. [file 10495_2026_2401_MOESM6_ESM.tif]
