# Supplementary material for: IRF1-mediated sensing of oxidized mitochondrial DNA drives macrophage PANoptosis in lung ischemia–reperfusion injury
Source: Apoptosis. 2026 Jul 25;31(8):198. doi: 10.1007/s10495-026-02401-3 (PMC13401555; doi:10.1007/s10495-026-02401-3)
Supplement: Supplementary file 7 — Supplementary Material 7. [file 10495_2026_2401_MOESM7_ESM.pdf]

# IRF1-mediated sensing of oxidized mitochondrial DNA drives macrophage PANoptosis in lung ischemia-reperfusion injury

Nan Zhang[1,*], Zhiyuan Zhang[1,2,*], Jing Yu[3,*], Yu Fu[1], Jiameng Gao[1], Xuemei Jiang[1], Yang Jin[1], Chang Chen[4,**], Zongmei Wen[1,**]

[1] *Department of Anesthesiology, Shanghai Pulmonary Hospital, School of Medicine, Tongji University, Shanghai, China*

[2] *Department of Anesthesiology and Critical care, Shanghai First Maternity and Infant Hospital, School of Medicine, Tongji University, Shanghai, China*

[3] *Department of Anesthesiology, Shanxi Bethune Hospital, Shanxi Academy of Medical Sciences, Third Hospital of Shanxi Medical University, Tongji Shanxi Hospital, Taiyuan, China*

[4] *Department of Thoracic Surgery, Shanghai Pulmonary Hospital, School of Medicine, Tongji University, Shanghai, China*

[*] These authors have contributed equally to this work.

**Corresponding author. Zhengmin Road, Yangpu District, Shanghai, 200433, China. chenthoracic@163.com (C. Chen)

**Corresponding author. Zhengmin Road, Yangpu District, Shanghai, 200433, China. wzm1103@126.com, +86 13761635280 (Z. Wen)

**Supplementary Table S1. Basic characteristics of patients with LTx**

| **Variables** | **LTx patients (n = 30)** |
|---|---|
| Demographics & Biometrics | |
| Age, y | 58.0 (52.8-62.5) |
| Male, n (%) | 23 (76.7%) |
| BMI, $kg/m^2$ | 25.88 ± 2.32 |
| Primary disease, n (%) | |
| ILD | 20 (66.7%) |
| COPD | 7 (23.3%) |
| Other | 3 (10.0%) |
| Comorbidities, n (%) | |
| Hypertension | 9 (30.0%) |
| Cardiovascular disease | 2 (6.7%) |
| Diabetes | 5 (16.7%) |
| Perioperative Data | |
| Intraoperative PRBC transfusion, U | 3.5 (2-8) |
| Intraoperative Plasma transfusion, mL | 1600 (1000-2000) |
| Post-operative PRBC transfusion (within 72 h), U | 2 (1-4) |
| Post-operative Plasma transfusion (within 72 h), mL | 200 (200-400) |
| Cold ischemia time (CIT), min | 413 ± 8.82 |
| Warm ischemia time (WIT), min | 42.58 ± 2.83 |
| Procedure type, n (%) | |
| SLT | 18 (60%) |
| BLT | 12 (40%) |
| Standardized Immunosuppressive Regimen, n (%) | |
| Basiliximab (Induction) | 30 (100%) |
| Tacrolimus + MMF + Corticosteroids (Maintenance) | 30 (100%) |
| Donor Characteristics (Organ Data) | |
| Donor Age, y | 41 (33-51) |
| Donor Sex (Male), n % | 25 (83.3%) |
| Outcomes | |
| PGD, n (%) | 6 (20%) |

Note: Continuous variables are expressed as mean ± SD or median (P25, P75); categorical variables are expressed as n (%). To control for variables impacting systemic inflammation, all patients were managed under a strict, standardized perioperative fluid management and restrictive transfusion protocol. Intraoperative blood product usage was minimized and recorded. All patients received a uniform immune-induction and triple-drug maintenance immunosuppressive regimen. Abbreviations: LTx, lung transplantation; BMI, body mass index; ILD, interstitial lung disease; COPD, chronic obstructive pulmonary disease; PRBC, packed red blood cells; SLT, single lung transplant; BLT, double lung transplant; MMF, mycophenolate mofetil; CIT, cold ischemia time; WIT, warm ischemia time (anastomosis time); PGD, primary graft dysfunction.

**Supplementary Table S2. List of primary antibodies used for immunofluorescence**

| Target Protein | Application | Dilution | Catalog Number | Manufacturer |
|---|---|---|---|---|
| NLRC5 | Cells, Tissue | 1:500 | sc-515668 | Santa Cruz, USA |
| ASC | Cells, Tissue | 1:500 | sc-514414 | Santa Cruz, USA |
| RIPK3 | Cells, Tissue | 1:500 | sc-374639 | Santa Cruz, USA |
| CASP8 | Cells, Tissue | 1:500 | 13423-1-AP | Proteintech, China |
| Tom20 | Cells | 1:500 | sc-17764 | Santa Cruz, USA |
| 8-OHdG | Cells | 1:500 | sc-393871 | Santa Cruz, USA |
| phospho-Drp1 (Ser616) | Tissue | 1:3200 | #4494 | Cell Signaling Technology, USA |

**Supplementary Table S3. List of primary antibodies used for Western blot analysis**

| Target Protein | Dilution | Catalog Number | Manufacturer |
|---|---|---|---|
| IRF1 | 1:1000 | 11335-1-AP | Proteintech, China |
| GSDMD | 1:1000 | ab209845 | Abcam, China |
| CASP3(Pro and Cleaved) | 1:1000 | 19677-1-AP | Proteintech, China |
| CASP8(Pro and Cleaved) | 1:500 | 13423-1-AP | Proteintech, China |
| p-MLKL | 1:1000 | #37333 | Cell Signaling Technology, USA |
| MLKL | 1:1000 | AP14272b | Abgent, USA |
| NLRC5 | 1:500 | sc-515668 | Santa Cruz, USA |
| Drp1 | 1:1000 | #8570 | Cell Signaling Technology, USA |
| phospho-Drp1 (Ser616) | 1:3200 | #4494 | Cell Signaling Technology, USA |
| β-actin | 1:100000 | AC026 | Abclonal, USA |

**Supplementary Table S4. Sequences of primers for qRT-PCR**

| Species | Genes | Forward Primers (5’→3’) | Reverse Primers (5’→3’) |
|---|---|---|---|
| Mouse | mt-ND1 | TATCTCAACCCTAGCAGAAA | TAACGCGAATGGGCCGGCTG |
| Mouse | D-loop | AATCTACCATCCTCCGTGAAACC | TCAGTTTAGCTACCCCCAAGTTTAA |
| Mouse | Cytb | GCTTTCCACTTCATCTTACCATTTA | TGTTGGGTTGTTTGATCCTG |
| Mouse | Tert | CTAGCTCATGTGTCAAGACCCTCTT | GCCAGCACGTTTCTCTCGTT |
| Human | mt-ND1 | ATACCCATGGCCAACCTCCT | GGGCCTTTGCGTAGTTGTAT |
| Human | D-loop | CTAAATAGCCCACACGTTCC | TAGGATGAGGCAGGAATCAA |
| Human | Cytb | GCCTGCCTGATCCTCCAAAT | AAGGTAGCGGATGATTCAGCC |
| Human | Tert | CTAGCTCATGTGTCAAGACCCTCTT | GCCAGCACGTTTCTCTCGTT |

**Supplementary Table S5. Clinical and demographic characteristics of patients in the scRNA-seq cohort (GSE220797)**

| Characteristics | Case 1 | Case 2 | Case 3 | Case 4 | Case 5 | Case 6 |
|---|---|---|---|---|---|---|
| Donor Profile | | | | | | |
| Age (years) / Sex | 28 / M | 19 / M | 66 / M | 37 / M | 36 / M | 51 / M |
| Smoking history | Yes | No | Yes | Yes | No | Yes |
| Mean PAP (mmHg) | 39 | 43 | 38 | 19 | 28 | 25 |
| Cold Ischemia Time (h) | 6 | 5 | 8 | 3 | 7 | 6 |
| Recipient Profile | | | | | | |
| Age (years) / Sex | 55 / F | 43 / F | 65 / M | 64 / M | 67 / M | 71 / M |
| BMI (kg/m²) | 24.4 | 26.2 | 29.3 | 16.5 | 26.3 | 23.4 |
| Primary Diagnosis | COPD | ILD | ILD | COPD | COPD | ILD |
| Clinical Outcomes | | | | | | |
| S/F ratio (72 h) | 247 | 395 | 466 | 245 | 194 | 339 |
| PGD Grade (72 h) | 2 | 1 | 0 | 2 | 3 | 1 |

Note: Data adapted from Wong et al. (*Am J Transplant*, 2024). All patients underwent bilateral lung transplantation. PGD grading was performed 72 hours post-transplantation according to ISHLT criteria. Abbreviations: PAP, pulmonary arterial pressure; BMI, body mass index; COPD, chronic obstructive pulmonary disease; ILD, interstitial lung disease; S/F ratio, $SpO_2/FiO_2$ ratio; PGD, primary graft dysfunction.

**Supplementary Fig. S1 PANoptosis Pathways are Enriched in Lung IRI and Correlate with Proinflammatory Cytokines.**

(A–C) GSEA enrichment plots of bulk RNA-seq data (GSE203238) from lung tissues for apoptosis (A), necroptosis (B), and pyroptosis (C) gene sets in the IRI group.

(D–F) Concentrations of proinflammatory cytokines IL-1β (D), IL-6 (E), and TNF-α (F) in BALF, quantified by ELISA. Levels were significantly increased in IRI mice and reduced by heparin.

(G–O) Pearson correlation analyses of the protein levels of key PANoptosis effectors (normalized to β-actin) with the concentrations of proinflammatory cytokines in BALF. Strong positive associations are shown for N-GSDMD (G-I), Cleaved-CASP3 (J-L), and p-MLKL (M-O) with IL-1β, IL-6, and TNF-α.

Data in D-F are presented as mean ± SD. Statistical significance was determined by one-way ANOVA with Tukey's post-hoc test. For G-O, the Pearson correlation coefficient $R$ and $P$ value are indicated. *$P < 0.05$, **$P < 0.01$, ***$P < 0.001$, ****$P < 0.0001$.

**Supplementary Fig. S2 Bioinformatic and *In Vivo* Validation of the IRF1-NLRC5 Axis in Lung IRI.**

(A, B) Functional enrichment analyses of DEGs from bulk RNA-seq data. (A) Gene Ontology (GO) enrichment analysis visualized as a Circos plot, highlighting pathways related to inflammation and programmed cell death. (B) Dot plot of enriched Kyoto Encyclopedia of Genes and Genomes (KEGG) pathways.

(C, D) Heparin treatment attenuated the IRI-induced upregulation of IRF1 (C) and NLRC5 (D) protein levels in lung tissues from IRI mice, as shown by Western blot analysis and quantification.

(E) Increased expression of NLRC5 (red) in lung sections from IRI mice, shown by immunofluorescence. Cell nuclei are counterstained with DAPI (blue). Scale bar, 50 μm.

(F) Venn diagram illustrating the intersection of predicted IRF1 target genes from three independent transcription factor databases (ChEA, GTRD, ChIP-Atlas), with NLRC5 identified as a commonly predicted target.

(G, H) Molecular docking simulations predicting the structure of the NLRC5-containing PANoptosome complex (G) and a detailed view of the protein-protein interaction interfaces (H).

(I, J) *In vivo* physiological validation of IRF1 silencing. Administration of IRF1-targeting siRNA to IRI mice significantly mitigated macroscopic lung injury, as evidenced by alleviated pulmonary edema (reduced lung wet-to-dry weight ratio) (I) and restored respiratory gas exchange (improved $PaO_2/FiO_2$ ratio) (J).

(K, L) Quantitative cell viability analysis of MH-S macrophages exposed to ex-His to establish optimal treatment parameters. (K) Dose-response curve showing macrophage viability following treatment with various concentrations of ex-His (0-100 μg/mL) for 24 h. (L) Time-course curve showing macrophage viability over 24 h following treatment with 50 μg/mL ex-His. Viability was measured via CCK-8 assay.

Data in C, D are presented as mean ± SD (n = 3). Data in K, L are presented as mean ± SD (n = 5). Data in I, J are presented as mean ± SD (n = 6). Statistical significance was

determined by one-way ANOVA followed by Tukey's post-hoc test (C, D, I, J) or Dunnett's post-hoc test for comparisons against the untreated control group (K, L). $^*P < 0.05$, $^{**}P < 0.01$, $^{***}P < 0.001$, $^{****}P < 0.0001$.

**Supplementary Fig. S3 Single-Cell Profiling Reveals Macrophage Reprogramming toward a Proinflammatory and PANoptotic State.**

(A, B) Enriched Gene Ontology (GO) (A) and Kyoto Encyclopedia of Genes and Genomes (KEGG) (B) pathways in macrophages, based on DEGs identified between the Reperfusion and CIT groups. Note the significant enrichment of pathways related to inflammation and cell death post-reperfusion.

(C, D) Ridgeplots visualizing the enrichment of specific GO biological processes (C) and KEGG pathways (D) related to PANoptosis (e.g., apoptosis, necroptosis, NOD-like receptor signaling) in macrophages after reperfusion.

(E, F) Functional stratification of macrophage subgroups based on IRF1 expression. (E) Violin plots showing the expression of IRF1 and a composite immune activity score across four distinct macrophage subgroups. (F) UMAP visualization of the spatial distribution of these IRF1-defined macrophage subgroups, highlighting the clustering of proinflammatory subsets.

(G) Bar plot illustrating the shift in macrophage subpopulation composition between CIT and Reperfusion conditions.

(H–J) Characterization of macrophage activation states derived from pseudotime analysis. (H) UMAP projection of single-cell transcriptomes with cells colored according to their assigned states (Resting, Intermediate, Activating, Executing). (I, J) Stacked bar plots showing the proportion and distribution of these functional states across different experimental groups and along the inferred trajectory.

(K, L) GSEA of GO biological processes (K) and KEGG signaling pathways (L) in macrophages, comparing the Reperfusion and CIT samples.

(M) UMAP plot illustrating the overall transcriptional divergence of macrophages between CIT and Reperfusion conditions, highlighting the emergence of a distinct cell state post-reperfusion.

**Supplementary Fig. S4 Negative Control for Tissue Immunofluorescence.**

To validate the specificity of the immunofluorescence staining, representative lung sections from the IRI group were incubated with the secondary antibody alone (Secondary Ab only, omitting the primary antibody) and counterstained with DAPI. The absence of specific fluorescent signals in the red channel demonstrates the lack of non-specific secondary antibody binding, thereby confirming the reliability of the target protein signals (e.g., in Fig. 6C). Scale bar, 20 μm.

**Supplementary Fig. S5 Single-cell RNA-sequencing Quality Control and Cell Composition Analysis.**

(A) Violin plots displaying the distribution of key quality control parameters for each individual sample after quality filtering. The metrics include the number of detected genes per cell (nFeature_RNA, left), total unique molecular identifiers (nCount_RNA, middle), and the percentage of mitochondrial gene expression (percent.mt, right). The consistent distributions demonstrate robust data quality and uniformity across all patient samples.

(B) Scatter plots illustrating the correlation between sequencing depth (nCount_RNA) and mitochondrial percentage (percent.mt, left), as well as sequencing depth and the number of detected genes (nFeature_RNA, right). Cells are colored by sample. The expected robust correlation and the uniform mixing of colors indicate the absence of significant technical artifacts (such as multiplets or empty droplets) and minimal batch effects during the sequencing of these lung tissues.

(C) Bar plot summarizing the final number of high-quality cells retained for downstream analysis in each sample. The precise cell count is indicated above each bar, ensuring sufficient statistical power for subsequent clustering and downstream analyses in this lung transplantation cohort.

(D) Elbow plot displaying the standard deviation of each principal component (PC). A clear inflection point (elbow) is observed, justifying the optimal number of dimensions selected to capture the majority of true biological variance for downstream dimensionality reduction (e.g., UMAP) and cell clustering.

(E) Stacked bar plot showing the relative proportions of major cell types (Macrophages, AT1, AT2, Endothelial cells, etc.) in the CIT and Reperfusion groups.
